# Synthetic ion channel inhibitors enhance plant drought tolerance

**DOI:** 10.1038/s41467-026-75894-w

**Published:** 2026-07-27

**Authors:** Kanane Sato, Kyota Suzuki, Shunya Saito, Taishin Kakei, Megumi Kato, Masana Yazaki, Yasutaka Kawai, Mieko Arisawa, Nobuhisa Isaka, Toshio Yamaguchi, Matteo Grenzi, Laura Luoni, Masaru Kono, Yuki Hayashi, Toshinori Kinoshita, Farhan Aziz, Khurram Bashir, Motoaki Seki, Asuka Kamimura, Takumi Higaki, Jun Takeuchi, Yasushi Todoroki, Huifei Yin, Francisco Rubio, Jörg Kudla, Shintaro Munemasa, Yoshiyuki Murata, Masaru Tsujii, Yasuhiro Ishimaru, Alex Costa, Nobuyuki Uozumi

**Affiliations:** 1 Department of Biomolecular Engineering, Graduate School of Engineering, Tohoku University, Sendai, Japan; 2 Department of Bioscience & Biotechnology, Graduate School of Bioresource & Bioenviromental Sciences, Kyushu University, Fukuoka, Japan; 3 Department of Applied Life Sciences, Niigata University of Pharmacy and Medical and Life Sciences, Niigata, Japan; 4 Faculty of Pharmacy, Niigata University of Pharmacy and Medical and Life Sciences, Niigata, Japan; 5 Department of Biosciences, University of Milan, Milan, Italy; 6 Astrobiology Center, Mitaka, Japan; 7 Graduate School of Science, Nagoya University, Nagoya, Japan; 8 Institute of Transformative Bio-Molecules (WPI-ITbM), Nagoya University, Nagoya, Japan; 9 Department of Life Sciences, SBA School of Science and Engineering, Lahore University of Management Sciences, Lahore, Pakistan; 10 Plant Genomic Network Research Team, RIKEN Center for Sustainable Resource Science, Yokohama, Japan; 11 Plant Epigenome Regulation Laboratory, RIKEN Cluster for Pioneering Research, Wako, Japan; 12 Kihara Institute for Biological Research, Yokohama City University, Yokohama, Japan; 13 Graduate School of Science and Engineering, Saitama University, Saitama, Japan; 14 Graduate School of Science and Technology, Kumamoto University, Kumamoto, Japan; 15 Faculty of Agriculture, Shizuoka University, Shizuoka, Japan; 16 Research Institute of Green Science and Technology, Shizuoka University, Shizuoka, Japan; 17 Graduate School of Environmental and Life Science, Okayama University, Tsushima, Okayama, Japan; 18 Departamento de Nutrición Vegetal, Centro de Edafología y Biología Aplicada del Segura, Consejo Superior de Investigaciones Científicas, Campus de Espinardo, Murcia, Spain; 19 Institut für Biologie und Biotechnologie der Pflanzen (IBBP), Universität Münster, Münster, Germany; 20 Institute of Biophysics, National Research Council of Italy (CNR), Milan, Italy; 21 Present Address: Department of Global Agricultural Sciences, Graduate School of Agricultural and Life Sciences, The University of Tokyo, Bunkyo-ku, Japan

**Keywords:** Molecular engineering in plants, Ion channels, Permeation and transport, Plant cell biology, Transporters

## Abstract

Drought stress significantly threatens global food security. According to the FAO2024, agriculture absorbs up to 80% of drought impacts. Stomata are vital pores for gas exchange and transpiration in plants. Stomatal closure, which is crucial for drought tolerance, is regulated by ion transport systems. Here, we identify two inhibitors of plasma membrane voltage-dependent potassium (K^+^) channels, NS5806 and UA49, that induce stomatal closure, reduce guard cell K^+^ levels, and increase drought resistance in Arabidopsis plants. This chemical-induced stomatal closure pathway is distinct from the abscisic acid (ABA). Notably, K^+^ channel inhibition led to increased cytosolic Ca^2+^, which was absent in K^+^ inward channel mutants, highlighting the link between K^+^ channel activity and cytosolic Ca^2+^ elevation. These findings suggest that the chemical regulation of K^+^ channels represents a strategy to induce stomatal closure, potentially improving plant drought tolerance through targeted interventions.

## Abstract (web summary)

Drought stress significantly threatens global food security by limiting plant growth. Here, authors demonstrate that chemical inhibition of potassium channels induces stomatal closure and enhances drought resistance, offering a crop protection strategy.

## Introduction

Stomata are vital structures for plant survival because they facilitate gas exchange and transpiration, which are essential for nutrient uptake from the air and the soil. Drought poses a serious global threat to food production, prompting various studies aimed at enhancing drought tolerance by focusing on the regulation of stomata^1^. Several pathways are involved in stomatal closure; of those, the ABA signaling pathway is the most well-studied at the molecular level. ABA binding to PYR/PYL/RCAR receptors initiates the activation of Ca^2+^-independent OST1/SnRK2.6 kinase, resulting in the activation of anion efflux channels, such as SLAC1^2,3^ and concomitantly K^+^ efflux channels, such as GORK. In parallel, as a Ca^2+^-dependent pathway, the influx of Ca^2+^ into guard cells occurs through plasma membrane Ca^2+^ permeable (*I*_Ca_) channels activated by the ABA-induced inhibition of the phosphatase ABI2, a process mediated by reactive oxygen species and the hyperpolarization of the plasma membrane^4–6^, this is followed by Ca^2+^-induced Ca^2+^ release from internal membrane stores, leading to the activation of Ca^2+^-binding proteins which further enhances the activity of SLAC1 and GORK, eventually leading to stomatal closure^7–9^. Changing the plasma membrane potential or artificially generating Ca^2+^ signals in guard cells can induce stomatal closure^10–13^.

To protect plants from drying, film-forming antitranspirants are sometimes used to seal stomata, and metabolic compounds, such as the drought stress hormones ABA and chitosan, are applied to promote growth in cultivated plants^14,15^. However, these methods have unintended consequences beyond improving drought tolerance. ABA agonists, which are compounds that induce stomatal closure, and H^+^-ATPase inhibitors that promote stomatal opening, also increase plant drought tolerance^16–18^. As a link to the external space, ion transport systems, such as voltage-dependent K^+^ channels, anion channels and Ca^2+^ channels, play crucial roles in optimizing the response of plant cells to various environmental changes. Therefore, ion channel modulators are powerful tools for gaining insights into plant molecular signal transduction mechanisms. These compounds could also be useful in alleviating drought stress through stomata. Unfortunately, effective inhibitors of ion transporters in guard cells are currently lacking.

When ion channels are targeted to induce stomatal closure, controlling the cytosolic Ca^2+^ concentration may be an effective approach. While several plasma membrane Ca^2+^ channels have been implicated in stomatal closure, their precise identities and regulatory mechanisms are still being fully elucidated^19–22^. Obtaining chemical activators for K^+^ efflux systems (such as GORK) or Cl^−^ efflux systems (such as SLAC1) seems to be more challenging than obtaining inhibitors. K^+^ channels in the plasma membrane involved in stomatal movement have been well-characterized: the inactivation of the inward-rectifying KAT1, which functions in the plasma membrane of guard cells, reduces the stomatal aperture in the osmotically driven process, and a dominant-negative *KAT1* mutation suppresses light-induced stomatal opening^23–25^. On the basis of these findings, we develop two voltage-dependent K^+^ channel inhibitors. The application of these inhibitors enhanced drought resistance in plants. Furthermore, these compounds increased cytosolic Ca^2+^ levels in guard cells. This increase was not detected in mutants in which the expression of K^+^ inward channels was expressed, indicating a relationship between the regulation of K^+^ channel activity and Ca^2+^ concentration dynamics. These results led to the development of stomatal closure inducers, highlighting the potential of biostimulants to increase plant survival under stress conditions and revealing a stomatal closure pathway distinct from ABA-mediated pathways.

## Results

### NS5806 and its analog UA49 inhibit voltage-dependent K^+^ channels

**Fig. 1:**
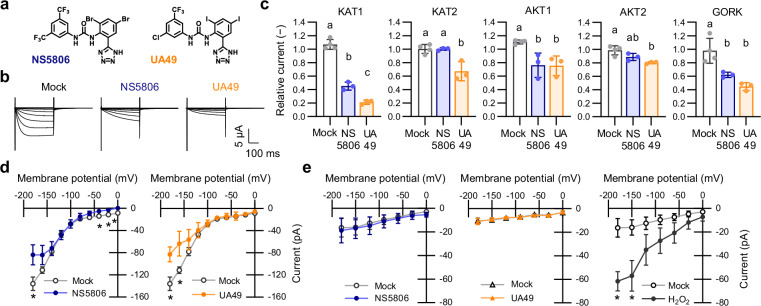
UA49 is designed to compound as a more potent inhibition of the K_in_ channel from NS5806. **a** Chemical structure of NS5806 and UA49. **b** Representative current traces from *Xenopus laevis* oocyte expressing KAT1 with or without 30 µM NS5806 or UA49 in the external buffer. **c** Effect of NS5806 or UA49 on KAT1, KAT2, AKT1, AKT2, and GORK. Two-electrode voltage clamp was performed using *Xenopus laevis* oocytes for each channel with or without 30 µM NS5806 or UA49 in the bath solution, and relative currents were plotted (mock: *n* = 4; NS5806: *n* = 3; UA49: *n* = 3, biological replicates, mean ± SD). Zero point two percent DMSO was used as a mock. The current value was collected at the end of the pulse at −170 mV for KAT1, KAT2, and AKT1, −155 mV for AKT2 and +50 mV for GORK. Bars marked with different letters are significantly different (*p* < 0.05) by one-way ANOVA with Tukey–Kramer test. **d** Patch clamp recording of K_in_ channels. Current–voltage relationships of K_in_ currents with or without 10 μM NS5806 (left) or UA49 (right). Mock (0.2% DMSO) data is the same for NS5806 and UA49. Error bars represent SEM. Asterisks indicate significant differences between Mock and NS5806 or UA49 at different time points (Mock: *n* = 5; NS5806: *n* = 3; UA49: *n* = 3, biological replicates, * *p* < 0.05; two-tailed Student’s *t* test). **e** Patch clamp recording of *I*_Ca_ channels. Current–voltage relationships of *I*_Ca_ currents with or without 10 μM NS5806 (left), UA49 (center), or 1 mM H_2_O_2_ (right). Mock (0.2% DMSO) data is the same for H_2_O_2_ and NS5806. Error bars represent SEM. Asterisks indicate significant differences between Mock and NS5806 or UA49 at different time points (Mock: *n* = 4; NS5806: *n* = 4; UA49: *n* = 3, biological replicates, *p < 0.05; two-tailed Student’s *t* test).

We conducted a chemical screen of an in-house collection of 135 commercially available compounds for inhibitors of KAT1, a dominant voltage-dependent inward K^+^ channel in Arabidopsis guard cells, by using the two-electrode voltage-clamp technique with *Xenopus* oocytes. Among the tested chemicals, NS5806 inhibited KAT1 activity by 55% (Fig. 1a–c). The other K^+^ channels in guard cells, KAT2, AKT1, AKT2, and GORK, were inhibited by less than 50% by NS5806 (Fig. 1c). To develop more effective KAT1 inhibitors from NS5806, we tested NS5806 analogs with different functional groups at the three R_1_–R_3_ sites in NS5806 (Supplementary Fig. 1a–d). On the basis of our measurements of KAT1 inhibition, we designed UA49, where one trifluoromethyl residue was replaced by a chloride residue at the R_1_ site and where two bromine residues were replaced by iodine residues at the R_2_ site, increasing its electron-withdrawing properties (Fig. 1a and Supplementary Fig. 1a–d). We found that the substitution of a trifluoromethyl or chloride residue at the R_1_ site or a halogen residue at the R_2_ site did not alter the inhibition of GORK (Supplementary Fig. 1e). UA49 inhibited KAT1 activity by 79% (Fig. 1a–c). UA49 inhibited KAT2 more strongly than NS5806 did (Fig. 1c). NS5806 and UA49 inhibited KAT1 in a dose-dependent manner (Supplementary Fig. 2), and the shifts in the tail current *I–V* relationships suggest a potential effect on channel gating (Supplementary Fig. 3). Through a strategy grounded in rational design, we successfully modified NS5806 to create a potent inhibitor of Arabidopsis voltage-dependent K^+^ channels. To investigate the ion channel regulatory activity of NS5806/UA49 in guard cells, we performed whole-cell patch clamp recordings with Arabidopsis guard cell protoplasts. NS5806/UA49 inhibited inward K^+^ currents at the plasma membrane (Fig. 1d). Additionally, we examined whether NS5806/UA49 directly affects Ca^2+^ permeable cation (*I*_Ca_) channels (Fig. 1e). Patch clamp recordings of the plasma membrane in guard cells revealed no effect on *I*_Ca_ channels, whereas H_2_O_2_ clearly activated them. These data show that NS5806/UA49 negatively regulates voltage-dependent K^+^ channel activity in guard cells.

### NS5806/UA49 are effective chemical compounds for inducing drought tolerance in plants

**Fig. 2:**
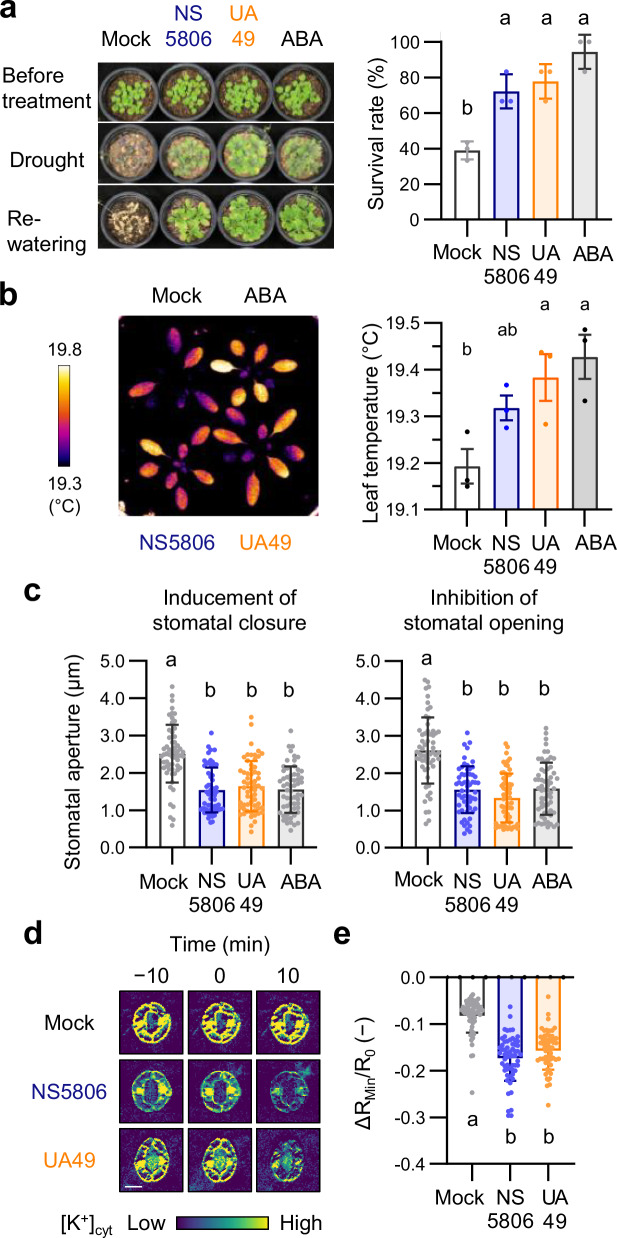
NS5806 and UA49 induce stomatal closure and enhance drought stress tolerance. **a** Drought stress tolerance test for *A. thaliana* with 0.1% DMSO (mock), 100 µM NS5806, UA49, or 10 µM ABA. Representative image are shown in left panel, and analysis of the survival rate are shown in right panel. Data are mean ± SD, the experiments comprised three replicants (5 plants per pot, 6 pots per replication). Bars marked with different letters are significantly different (*p* < 0.05) by one-way ANOVA with Tukey–Kramer test. **b** Representative false-color infrared image of plants treated with 0.1% DMSO (Mock), 100 µM NS5806, UA49, or 10 µM ABA. The infrared thermography was taken 3 h after the treatment. Three independent experiments were performed to calculate the average temperature. Bars marked with different letters are significantly different (*p* < 0.05) by one-way ANOVA with Tukey–Kramer test (mean ± SEM). **c** The stomatal aperture of *A. thaliana* leaves in response to light. For the light-to-light condition (test for inhibition of stomatal closure, left), epidermal strips were pre-incubated for 2 h under light to induce stomatal opening and followed by a 2 h incubation under light with 0.2% DMSO (Mock), 10 µM NS5806, UA49, or ABA. For the dark-to-light condition (test for inhibition of stomatal opening, right), epidermal strips were pre-incubated for overnight under dark to induce stomatal closure and followed by 3 h incubation under light with 0.2% DMSO (mock), 10 µM NS5806, UA49, or ABA. Data are shown as mean ± SD (*n* = 59–60). Bars marked with different letters are significantly different (*p* < 0.05) by one-way ANOVA with Tukey–Kramer test. **d** False-color images illustrating cpVenus/CFP ratios in guard cells expressing lc-LysM GEPII 1.0 for measuring [K^+^]_cyt_ at the indicated time and time course data of representative normalized cpVenus/CFP ratios. The leaf epidermal strips were treated with 0.2% DMSO (Mock), 10 μM NS5806, or UA49 at 0 min. To compose the panel, images were cropped. Low cpVenus/CFP ratios indicating low [K^+^]_cyt_ are shown in black, and high cpVenus/CFP ratios indicating high [K^+^]_cyt_ are shown in white. Scale bar = 10 μm. **e** Peak cpVenus/CFP ratios as Δ*R*/*R*_0_ minimum decrease after mock, NS5806, or UA49 treatment. Data are shown as mean ± SD. Bars marked with different letters are significantly different (*p* < 0.05) by one-way ANOVA with Tukey–Kramer test (*n* = 60, biological replicates). Data were obtained in the same experimental method as in (**d**). The representative time course data are shown in Supplementary Fig. 4a.

To assess the agricultural utility of NS5806/UA49, we investigated whether NS5806/UA49 confers drought tolerance to plants (Fig. 2a). The adaxial leaf surfaces of 17-day-old wild-type *Arabidopsis thaliana* (Col-0) plants were sprayed twice with NS5806/UA49 at 2-day intervals. Subsequently, the plants were subjected to 13 days of drought stress by withholding water. Rehydration and the examination of plant survival rates revealed that NS5806/UA49 protected plants to a similar extent as ABA did. Additionally, thermal imaging revealed that UA49 treatment induced an increase in leaf temperature, similar to ABA, compared with untreated controls (mock). Although no statistically significant difference was observed between mock and NS5806, a trend toward higher leaf temperature was also detected in NS5806-treated plants, suggesting that both UA49 and NS5806 may promote stomatal closure (Fig. 2b). The treatment of plants with NS5806/UA49 under light-to-light or dark-to-light conditions led to stomatal closure (Fig. 2c). These findings showed that NS5806/UA49 can both promote stomatal closure and inhibit stomatal opening. We analyzed the effect of NS5806/UA49 on the K^+^ concentration in guard cells via the K^+^ sensor plant lc-LysM GEPII 1.0, which is a FRET-based K^+^ sensor with a *K*_d_ of 27 mM^26,27^ (Fig. 2d, e, and Supplementary Fig. 4a). NS5806/UA49 treatment resulted in a decrease in the cytosolic K^+^ concentration in guard cells, indicating either the induction of K^+^ efflux or the inhibition of K^+^ influx, which normally occurs during stomatal closure. Next, we assessed the impact of NS5806/UA49 on primary root elongation and seed germination. NS5806/UA49 did not adversely affect either of these processes, in contrast to treatment with ABA (Supplementary Fig. 4b, c). Treatment with NS5806/UA49 did not cause apparent cytotoxicity. Taken together, NS5806/UA49 specifically acted on guard cells to increase drought tolerance, with no side effects compared with those observed with ABA.

### The stomatal closure induced by NS5806/UA49 has limited involvement in the ABA pathway

**Fig. 3:**
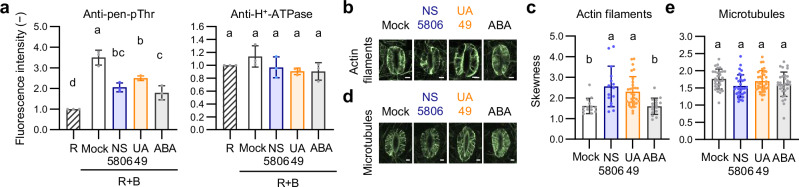
NS5806 and UA49 induce stomatal closure through a different mechanism from ABA. **a** The effect of NS5806 and UA49 on the phosphorylation of PM H^+^-ATPase in guard cells. Leaf epidermal tissues treated with 0.1% DMSO (mock), 10 μM NS5806, UA49, or ABA were illuminated with red light (R) or blue light superimposed on red light (R+B). The phosphorylated PM H^+^-ATPase (left) or PM H^+^-ATPase (right) was detected by immunohistochemical staining with anti-pen-pThr or anti-H^+^-ATPase antibodies. Relative fluorescence intensities are shown with relative to R set to 1.0. Data are shown as mean ± SD (*n* = 3). Bars marked with different letters are significantly different (*p* < 0.05) by one-way ANOVA with Tukey–Kramer test. **b**, **c** The effect of NS5806 and UA49 for guard cell actin cytoskeleton. Cotyledon of 14-day-old plants were treated with 0.1% DMSO (mock), 10 μM NS5806, UA49, or ABA for 5 min, then the cotyledons were observed using the confocal microscope. The representative images are shown in (**b**). Scale bars = 5 μm. Data are shown as mean ± SD. Bars marked with different letters are significantly different (*p* < 0.05) by one-way ANOVA with Tukey–Kramer test (Mock: *n* = 14; NS5806: *n* = 15; UA49: *n* = 30; ABA: *n* = 16, biological replicates). **d**, **e** The effect of NS5806 and UA49 for guard cell microtubules. Cotyledon of 14-day-old plants were treated with 0.1% DMSO (Mock), 10 μM NS5806, UA49, or ABA for 5 min, then the cotyledons were observed using the confocal microscope. The representative images are shown in **d**. Scale bars = 5 μm. Data are shown as mean ± SD. Bars marked with different letters are significantly different (*p* < 0.05) by one-way ANOVA with Tukey–Kramer test (Mock: *n* = 30; NS5806: *n* = 31; UA49: *n* = 33; ABA: *n* = 31, biological replicates).

Phosphorylation of the plasma membrane H^+^-ATPase is required for light-induced stomatal opening and is inhibited in ABA-induced stomatal closure^28^. NS5806/UA49 suppressed the phosphorylation level of H^+^-ATPase, similar to ABA (Fig. 3a). The cytoskeleton, which consists of actin filaments and microtubules, is involved in stomatal movement^29,30^. While NS5806/UA49 increased actin filament skewness within 5 min, indicating that bundling occurred, ABA did not (Fig. 3b, c). On the other hand, none of the treatments had any effect on microtubule formation (Fig. 3d, e). To reveal differences in the pathways leading to stomatal closure after treatment with NS5806/UA49 or ABA, we evaluated gene expression levels 3 h after the application of the compounds to the abaxial surface of the leaves (Supplementary Fig. 5). The upregulated genes outnumbered the downregulated genes after the application of ABA, whereas after the application of NS5806/UA49, this pattern was reversed. The number of upregulated genes was significantly lower for NS5806 (266) and UA49 (188) than for ABA (1133). Sixty-six percent of the gene upregulated by NS5806 (107 + 68) and 78% of the genes upregulated by UA49 (107 + 39) were also upregulated by ABA. On the other hand, the number of downregulated genes was more similar for treatment with the NS5806, UA49, and ABA. Thirty-seven percent of the genes downregulated by NS5806 (130 + 109) and 36% of genes down-regulated by UA49 (130 + 28) were also downregulated by ABA. Gene ontology (GO) analysis revealed that the expression of ABA-responsive genes was markedly upregulated after ABA treatment, whereas only a subset of ABA-responsive genes exhibited altered expression levels following NS5806/UA49 treatment (Supplementary Figs. 6 and 7). The number of genes altered by NS5806/UA49 was lower than that altered by ABA.

**Fig. 4:**
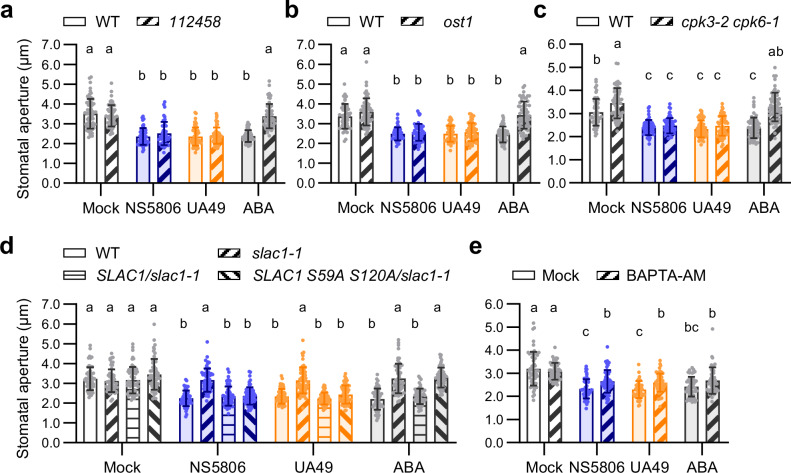
Stomatal responses by NS5806 and UA49 treatments under light-to-light condition (test for inducement of stomatal closure). The stomatal aperture was measured using *A. thaliana* mutants. Epidermal strips were pre-incubated for 2 h under light to induce stomatal opening and followed by a 2 h incubation under light with 0.2% DMSO (mock), 10 µM NS5806, UA49, or ABA. **a**, **b** Characteristic genes in the ABA pathway; *pyr1 pyl1 ply2 pyl4 pyl5 pyl8* (*112458*) (WT mock: *n* = 60; NS5806: *n* = 60; UA49: *n* = 60; ABA: *n* = 59, *112458* mock: *n* = 59; NS5806: *n* = 58; UA49: *n* = 60; ABA: *n* = 58, biological replicates) and *ost1* (**b** WT mock: *n* = 60; NS5806: *n* = 60; UA49: *n* = 60; ABA: *n* = 60, *ost1* Mock: *n* = 59; NS5806: *n* = 60; UA49: *n* = 60; ABA: *n* = 60, biological replicates). **c** Ca^2+^-dependent protein kinases; *cpk3-2 cpk6-1* (WT mock: *n* = 60; NS5806: *n* = 60; UA49: *n* = 60; ABA: *n* = 60, *cpk3-2 cpk6-1* mock: *n* = 60; NS5806: *n* = 60; UA49: *n* = 60; ABA: *n* = 60, biological replicates). **d** The anion channel required for stomatal closure; *slac1*, *SLAC1/slac1-1* and *SLAC1 S59A S120A/slac1-1* (WT mock: *n* = 60; NS5806: *n* = 58; UA49: *n* = 60; ABA: *n* = 59, *slac1-1* mock: *n* = 57; NS5806: *n* = 60; UA49: *n* = 58; ABA: *n* = 60, *SLAC1/slac1-1* mock: *n* = 60; NS5806: *n* = 60; UA49: *n* = 59; ABA: *n* = 59, *SLAC1 S59A S120A/slac1-1* mock: *n* = 60; NS5806: *n* = 60; UA49: *n* = 57; ABA: *n* = 60 biological replicates). **e** Requirement of cytosolic Ca^2+^ in stomatal closure; WT with BAPTA-AM (mock: *n* = 60; NS5806: *n* = 60; UA49: *n* = 59; ABA: *n* = 60, BAPTA-AM mock: *n* = 60; NS5806: *n* = 60; UA49: *n* = 58; ABA: *n* = 59, biological replicates). Data are shown as mean ± SD. Bars marked with different letters are significantly different (*p* < 0.05) by one-way ANOVA with Tukey–Kramer test.

To explore which stomatal closure pathways were affected by NS5806/UA49, we examined stomatal aperture in Arabidopsis mutants under light-to-light conditions (Fig. 4). NS5806/UA49 induced stomatal closure in mutants lacking genes known to be involved in the ABA pathway, major ABA receptors (*pyr1/pyl1/pyl2/pyl4/pyl5/pyl8*)^31^ or *ost1*^32^ (Fig. 4a, b). We confirmed that NS5806 did not bind to ABA receptors (Supplementary Fig. 8). NS5806/UA49 also induced stomatal closure in the *cpk3-2 cpk6-1* mutant lacking two Ca^2+^-dependent protein kinases that function in ABA-dependent stomatal closure^7^ (Fig. 4c). Furthermore, NS5806/UA49 induced stomatal closure in the pathogen response pathway mutants, *cas-1*^33^ and *eds5-1*^33,34^ (Supplementary Fig. 9a). In contrast, NS5806/UA49 did not cause stomatal closure in *slac1-1*, a mutant lacking an anion channel required for stomatal closure^35^ (Fig. 4d). Interestingly, the induction of stomatal closure by NS5806/UA49 could be recovered when *slac1-1* was complemented with a version of SLAC1 in which the Ser at positions 59 and 120 was replaced with Ala. Both of these residues are phosphorylated during ABA-induced stomatal closure^36^. Therefore, NS5806/UA49 likely activated SLAC1 through different kinases than ABA did. Moreover, the chelation of cytoplasmic Ca^2+^ with BAPTA-AM reduced stomatal closure caused by NS5806/UA49 (Fig. 4e). These results indicate that stomatal closure by NS5806/UA49 requires SLAC1 and is mediated, in part, by Ca^2+^ signals, which are not identical to those of the ABA and pathogen response pathways. Similar results were confirmed by a dark-to-light test, which assessed the NS5806/UA49-mediated inhibition of stomatal opening (Supplementary Fig. 9). These data confirmed that NS5806/UA49 can both induce stomatal closure and suppress stomatal opening.

### NS5806/UA49 causes changes in the cytosolic Ca^2+^ concentration in guard cells via K^+^ inward channels

**Fig. 5:**
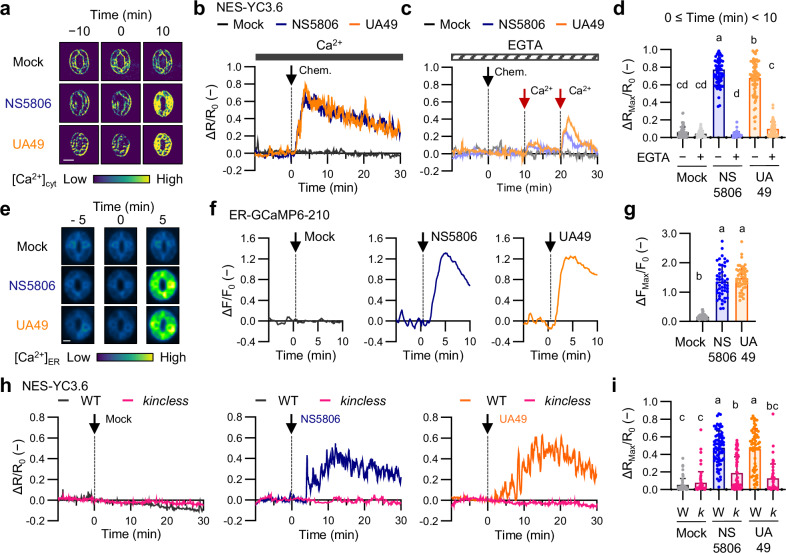
NS5806 and UA49 induce a sustained cytosolic Ca^2+^ increase via K^+^ inward channels. **a**, **b** The effects of NS5806 and UA49 on cytosolic Ca^2+^ concentration ([Ca^2+^]_cyt_) in guard cells. False-color images illustrating cpVenus/CFP ratios in guard cells expressing NES-YC3.6 for measuring [Ca^2+^]_cyt_ at the indicated time are shown in (**a**). The epidermal strips were incubated for 3 h in a buffer solution (5 mM KCl, 10 mM MES, and 50 µM CaCl_2_, pH 6.15 adjusted with Tris-base) in the growth chamber under light and treated with 0.2% DMSO (mock), 10 μM NS5806, or UA49 at 0 min. To compose the panel, images were cropped. Low cpVenus/CFP ratios indicating low [Ca^2+^]_cyt_ are shown in black, and high cpVenus/CFP ratios indicating high [Ca^2+^]_cyt_ are shown in white. Scale bar = 10 μm. A representative set of replicates of a complete time-series each is shown in Supplementary Movies 1–3. Time course data of representative normalized cpVenus/CFP ratios are shown in (**b**). **c**, **d** The effect of Ca^2+^ chelator EGTA on NS5806/UA49 related Ca^2+^ behavior. Epidermal strips were fully chelated for apoplastic Ca^2+^ by EGTA for 25 min prior to adding chemical solutions. Half of the solution filling the chamber was replaced with fresh NS5806/UA49 solution without EGTA at 10 min and 20 min after the initial addition of NS5806/UA49. Representative time course data are shown in (**c**). Peak cpVenus/CFP ratios as Δ*R*/*R*_0_ maximum increase with or without EGTA (0 ≤ time (min) < 10) are shown in (**d**). Data are plotted as mean ± SD from 0 min to 10 min (*n* = 72). **e**–**g**, Ca^2+^ imaging was performed with Arabidopsis ER-GCaMP6-210 expressing line. Guard cells were treated with 10 μM NS5806/UA49 at 0 min as indicated in the graph. The first 300 seconds were removed to wait for the stabilization of the signal. **e** False-color shows ER-GCaMP6-210 fluorescence intensity at the indicated time in guard cells. **f** The representative time course data of normalized GCaMP6 fluorescence intensity. **g** The peak normalized fluorescence intensity as Δ*F*/*F*_0_ maximum (mean ± SD, *n* = 48). Bars marked with different letters are significantly different (*p* < 0.05) by one-way ANOVA with Tukey–Kramer test. **h**, **i** The effect of K^+^ inward channels on NS5806/UA49 related Ca^2+^ behavior. The leaf epidermal strips were treated with 0.2% DMSO (mock), 10 μM NS5806, or UA49 at 0 min. Time course data of representative normalized cpVenus/CFP ratios of NES-YC3.6 expressed in *kincless* are shown in (**h**). Peak cpVenus/CFP ratios as Δ*R*/*R*_0_ maximum increase (0 ≤ time (min) ≤ 20) are shown in (**i**). “W” and “*k*” indicate “wild type” and “*kincless*”, respectively. Data are plotted as mean ± SD (*n* = 60). Bars marked with different letters are significantly different (*p* < 0.05) by one-way ANOVA with Tukey–Kramer test.

Since NS5806/UA49-treated plants were dependent on internal Ca^2+^ during stomatal closure (Fig. 4e), we examined whether the impact of NS5806/UA49 on K^+^ channel activity affected Ca^2+^ oscillations in guard cells (Fig. 5). Wild-type *Arabidopsis thaliana* (Col-0) expressing the Ca^2+^ indicator FRET (Förster resonance energy transfer) based protein NES-YC3.6, which senses Ca^2+^ in vitro with a *K*_d_ of 250 nM^37,38^, was used for monitoring temporal changes in cytoplasmic Ca^2+^ concentrations. The addition of NS5806/UA49 rapidly increased the Ca^2+^ concentration in guard cells, and the increase in Ca^2+^ concentration lasted for at least 30 min (Fig. 5a, b). A representative video is available in Supplementary Movies 1–3. These Ca^2+^ characteristics are distinct from the Ca^2+^ oscillation profile observed after ABA or flg22 application^10,39,40^. However, the strong increase in cytosolic Ca^2+^ caused by NS5806/UA49 was not observed when guard cells were treated with EGTA to chelate apoplastic Ca^2+^ (Fig. 5c, d). When half of the solution in the chamber was replaced with a solution containing NS5806 or UA49 and Ca^2+^ at 10 min and 20 min, the cytosolic Ca^2+^ levels increased. The increase in cytosolic Ca^2+^ levels by NS5806/UA49 was not observed in pavement cells or root tips (Supplementary Fig. 10), supporting the conclusion that the action of NS5806/UA49 occurs rather specifically in guard cells. Additionally, to directly monitor Ca^2+^ dynamics within the endoplasmic reticulum (ER), we used Arabidopsis lines expressing the ER-localized Ca^2+^ indicator, ER-GCaMP6-210^41^. Our analyses in guard cells revealed that treatment with NS5806/UA49 also induced an increase in Ca^2+^ levels in the ER (Fig. 5e–g), supporting the conclusion that the apoplast is the main source of Ca^2+^ sustaining the cytosolic Ca²⁺ increase. Such changes of cytosolic Ca^2+^ levels in guard cells are key signatures of responses to various stimuli, mediated by specific channels, such as hydrogen-peroxide-induced Ca^2+^ increases (HPCA1) for H_2_O_2_^21^, hyperosmolality-gated Ca^2+^ permeable channel 1.1 (OSCA1.1) for osmotic stress^19^, and OSCA1.3/1.7 for pathogen signaling^20^. To determine whether NS5806 and UA49 affect these pathways, we performed Ca^2+^ imaging in the cytosol in their respective mutant backgrounds (Supplementary Fig. 11). While NS5806 affected the peak Ca^2+^ increase only in *osca1.3/1.7*, UA49 induced significant differences in all three lines (*hpca1-1*, *osca1-1*, and *osca1.3/1.7*) relative to the WT. Collectively, these data suggest that NS5806 signaling is partially dependent on OSCA1.3/1.7, whereas UA49 exhibits a broader overlap with HPCA1, OSCA1.1, and OSCA1.3/1.7 pathways to execute stomatal response. To determine the requirement for an increase in cytosolic Ca^2+^ for K^+^ channel inhibition, we used the *kincless* mutant, which lacks functional voltage-dependent inward K^+^ channels in the plasma membrane^42^. NS5806/UA49-induced Ca^2+^ elevation was abolished with the *kincless* mutant (Fig. 5h, i). Accordingly, the stomatal closure induced by NS5806/UA49 was impaired in the *kincless* mutant (Supplementary Fig. 12a). However, NS5806/UA49-induced stomatal closure did not occur in the *gork* mutant, suggesting that GORK is also involved in the stomatal closure mediated by these compounds (Supplementary Fig. 12b). These results indicate that the administration of NS5806/UA49 led to an increase in cytosolic Ca^2+^ through the regulation of K^+^ channel activity.

## Discussion

In this study, the KAT1 inhibitor NS5806/UA49, which promotes a decrease in the cytosolic K^+^ content in guard cells, conferring significant drought tolerance in Arabidopsis (Figs. 1 and 2), was developed and tested. The results of this study support the notion that an osmotic decrease in the cytosolic K^+^ content in guard cells is necessary for stomatal closure. The number of gene expression changes induced by NS5806/UA49 was smaller than the number of changes induced by ABA (Supplementary Fig. 5). Unlike ABA, NS5806/UA49 did not cause delays in germination or root elongation, suggesting minimal or no side effects (Supplementary Fig. 4b, c). Cell membrane proteins are crucial for regulating various processes, making them compelling targets for human therapeutic drug development^43^. Similarly, targeting proteins located in the plasma membrane of guard cells is one strategy to optimize plant physiological status. Our study demonstrated that the temporary or partial inhibition of KAT1 using NS5806/UA49 resulted in drought tolerance similar to that observed in the *KAT1* mutant^24^, effectively suppressing light-induced stomatal opening (Fig. 2c).

The dissection of the NS5806/UA49-mediated stomatal closure pathway revealed differences from the ABA-dependent response pathway (Figs. 3, 4, and 5). Treatment with NS5806/UA49 had similar effects on stomatal movement as that observed in the *pyr1/pyl1/pyl2/pyl4/pyl5/pyl8* mutant, the *ost1* mutant and the wild type (Fig. 4a, b), which is characterized as receptor-mediated Ca^2+^-independent pathway. While NS5806/UA49 required SLAC1 for stomatal closure, similar to the ABA pathway, the phosphorylation sites needed for SLAC1 activation were different (Fig. 4d). The distinct kinases likely contributed to the NS5806/UA49-mediated stomatal closing pathway. SLAC1 is a crucial anion efflux channel for reducing cytosolic anion content during guard cell shrinkage, which occurs in the conventional model of ABA-induced stomatal closure. Moreover, Ca^2+^ signaling is suggested to control actin filaments, and Ca^2+^ channels are proposed to be regulated by actin dynamics^29,44,45^. NS5806/UA49 induced actin filamentation for 5 min, which was not observed with ABA treatment (Fig. 3b, c), suggesting that the NS5806/UA49-mediated stomatal closure pathway is different from the ABA-mediated stomatal closure pathway. Another hallmark feature of NS5806/UA49-related stomatal closure is the unique generation of sustained high Ca^2+^ levels in guard cells (Fig. 5b), whereas ABA or flg22 induced repetitive oscillations with a transient increase in cytosolic Ca^2+^^10,39,40^. This phenomenon is reminiscent of stomatal closure thorough artificial cytosolic Ca²⁺ elevation generated by regulating increased cytosolic pH and Ca^2+^-induced Ca^2+^ release using channelrhodopsins in guard cells^10–12,46^. The influx of Ca²⁺ from the extracellular space is contributing factors to this process (Fig. 5c–g). Nevertheless, NS5806/UA49 did not directly inhibit the *I*_Ca_ channel in the patch clamp measurements (Fig. 1e). The defective K^+^ inward channel mutants *kincless*^42^ exhibited a marked decrease in cytosolic Ca^2+^ elevation caused by NS5806/UA49 (Fig. 5h, i). These results indicate that hyperpolarization caused by the NS5806/UA49-mediated inhibition of inward K^+^ channels, such as KAT1, might activate plasma membrane Ca²⁺ influx channels. In animal cells, K^+^ movement across the membrane directly influences the membrane potential^47^. A combination of K^+^ channels and Ca^2+^ channels cooperate in neurons and cardiac cells during Ca^2+^ influx and release, which subsequently regulates other K^+^ channels. Some reports have shown that changes in the plasma membrane potential affect Ca^2+^ channel activity in guard cells^10,48^. Therefore, similar to the situation in animal systems, connections between K^+^ channels and Ca^2+^ channels might also exist in the NS5806/UA49-mediated signaling pathway in guard cells.

The results of this study reveal a stomatal closure signaling pathway triggered by NS5806/UA49 treatment in Arabidopsis plants. This pathway differs from the ABA-induced signaling pathway, except for the decrease in cytosolic K^+^ concentrations and the requirement for SLAC1, which are common to the pathway. These findings suggest that NS5806/UA49 could serve as potential molecular tools for elucidating the common basis of stomatal closure mechanisms; furthermore, they may have potential applications as plant regulators and biostimulants in horticulture and agriculture.

## Methods

### Ethical statement

Xenopus experiments were carried out in accordance with the institutional guidelines.

### Chemical preparations

(*S*)-(+)-abscisic acid (ABA) was obtained from Tokyo Chemical Industry Co., Ltd. (TCI). NS5806 and its analogous compounds were synthesized. Please refer to the Supplementary Information for the compound synthesis method and NMR data. All compounds were dissolved in DMSO. The collection of 135 commercially available, animal channel regulators was kindly given to us by Sumitomo Chemical Co., Ltd., and each molecule is commercially available.

### Recordings in *Xenopus laevis* oocytes

To express KAT1, KAT2, AKT1, AKT2, GORK, and SLAC1 channels in *Xenopus laevis* oocytes, the respective cDNA was subcloned into a pYES2-derived vector (Invitrogen, Carlsbad, CA, USA)^49^. cRNAs were synthesized from *Not*I-linearized plasmids using an in vitro transcription kit (Ambion, Austin, TX, USA), and oocytes were injected with 10 ng of cRNA in 50 nL per oocyte, followed by incubation in Barth’s buffer (88 mM NaCl, 1 mM KCl, 0.41 mM CaCl_2_, 0.33 mM Ca(NO_3_)_2_, 1 mM MgSO_4_, 2.4 mM NaHCO_3_, 5 mM HEPES-NaOH and 50 mg/L gentamicin sulfate (pH 7.3)) at 18 °C for 1–3 days. For two-electrode voltage clamp recordings, KAT1, KAT2, AKT1, and AKT2 were tested in 120 mM K^+^ solution (120 mM KCl, 1 mM MgCl_2_, 1 mM CaCl_2_ 10 mM HEPES-NaOH (pH 7.3)). GORK was tested in 12 mM K^+^ solution (12 mM KCl, 108 mM NaCl, 1 mM MgCl_2_, 1 mM CaCl_2_ 10 mM HEPES-NaOH (pH 7.3)). SLAC1 was tested in 75 mM Na^+^ solution (75 mM NaCl, 20 mM CaCl_2_, 1 mM MgCl_2_, 10 mM HEPES, osmolality was adjusted with D-mannitol to 220–260 mOsm/L). Protocols for recording Arabidopsis K^+^ channels and SLAC1 are detailed elsewhere^50–54^. Recordings and data analysis were conducted using an AxoClamp 2B amplifier (Axon Instruments) and an Axon Digidata 1550 System (Molecular Devices).

### Patch-clamp recordings

Guard cell protoplasts were prepared from Arabidopsis rosette leaves as described previously^55,56^. For *I*_Ca_ current recording, the pipette solution contained BaCl_2_ at 10 mM, EGTA at 4 mM, and HEPES-Tris (pH 7.1) at 10 mM, osmolality was adjusted to 500 mOsm kg^−1^ with D-sorbitol. The bath solution contained BaCl_2_ at 100 mM, and MES-Tris (pH 5.6) at 10 mM, osmolality was adjusted to 485 mOsm kg^−1^ with D-sorbitol. NADPH at 5 mM was freshly added to the pipette solution. DTT at 0.1 mM was freshly added to the pipette and bath solutions. Whole-cell *I*_Ca_ currents were recorded using a CEZ-2200 patch-clamp amplifier (NIHON KOHDEN, Tokyo, Japan) and pCLAMP 8.1 software (Molecular Devices, Inc., CA, USA). Ramp voltage pulses were applied from +20 to −180 mV with −200 mV s^−1^ and the holding potential was 0 mV. Control currents were recorded 10 times with a 10-s interval. After the recording, NS5806 or UA49 was added to the bath solution, and then *I*_Ca_ currents were recorded 10 times with a 10-s interval. The average current obtained from the 10 traces was used for current–voltage curves. Leak currents were not subtracted, and the liquid junction potential was not corrected.

For inward K^+^ current recordings, the pipette solution contained KCl at 30 mM, K-Glu at 70 mM, CaCl_2_ at 3.35 mM, MgCl_2_ at 2 mM, EGTA 6.7 mM, HEPES-Tris (pH 7.1) at 10 mM, and Mg-ATP at 5 mM, osmolality was adjusted to 500 mOsm kg^−1^ with D-sorbitol. The bath solution contained KCl at 30 mM, MgCl_2_ at 2 mM, CaCl_2_ at 40 mM, and MES-Tris (pH 5.6) at 10 mM, osmolality was adjusted to 485 mOsm kg^−1^ with D-sorbitol. Inward K^+^ currents were recorded using a CEZ-2200 patch-clamp amplifier or CEZ-2400 patch-clamp amplifier (NIHON KOHDEN, Tokyo, Japan) and pCLAMP 8.1 software (Molecular Devices, Inc., CA, USA). Twenty millivolts decrease voltage step pulses with a duration of 1 s were applied from 0 to −180 mV. The holding potential was −40 mV. Guard cell protoplasts were incubated with NS5806 or UA49 for 10 min before the recording. Leak currents were not subtracted, and liquid junction potential was not corrected.

### Plant materials and growth conditions

Except as otherwise noted, all Arabidopsis lines were grown on soil under long-day conditions (16 h light/8 h dark, 80–135 μmol m^−2^ s^−1^) in a growth chamber at 22 °C. Unless otherwise specified, Columbia-0 (Col-0) was used as wild-type (WT) plants. Wassilewskija (WS) WT and *kincless* mutant plants were transformed with the NES-YC3.6 Ca^2+^ indicator via floral dipping, using the original *pTKan-NES-YC3.6* construct reported by Krebs et al.^57^. In this construct, the coding sequence of the sensor is controlled by the *UBQ10* promoter and *rbcs* terminator. Transformants were selected using a fluorescence stereomicroscope equipped with a GFP filter, isolating fluorescent seedlings. The original WS and *kincless* seeds were kindly provided by Hervè Sentenac (Institute for Plant Sciences of Montpellier).

### Leaf temperature measurements

Thirty-two-day-old plants were sprayed with water containing 0.1% DMSO, 10 µM ABA, 100 µM NS5806, or UA49, and after 3 h, the surface temperature was determined with a thermal camera FLIR A6700 MWIR (Teledyne FLIR LLC, U.S.A.). Image analysis was performed using ResearchIR software (Teledyne FLIR LLC, U.S.A.).

### Drought stress test

*Arabidopsis thaliana* (Col-0 ecotype) seeds were sown in Dio propagation mix no. 2 soil (DIO Chemicals, Tokyo, Japan). After a 3-day vernalization at 4 °C in darkness, the seedlings were transferred to a greenhouse and grown at 22 °C with a 16 h light (~100 µmol m^−2^ s^−1^ photon flux density)/8 h dark cycle for 17 days. Seventeen-day-old plants were sprayed with 625 μL of 0.1% DMSO (Mock), 100 μM NS5806, UA49, or 10 µM ABA and immediately started drought stress treatment. The second spray was performed 48 h after the first one. After the first spray, plants were immediately subjected to drought stress by ceasing the water supply and removing the water excess. For each treatment, six pots containing 5 plants each, harboring 30 plants in total, were used with three experimental replications (a total set of 90 plants per treatment). Experiments were repeated three times to confirm reproducibility. Plants were re-watered after 13 days for drought treatment, and the survival rate was recorded for each treatment.

### Stomatal aperture measurements

Stomatal aperture measurements were carried out as described previously^56^. Briefly, to examine stomatal responses to chemicals during light-to-light transitions, rosette leaves from 3 to 4-week-old plants were excised and blended for 30 s using a blender (700BUJ, WARING). The resulting material was filtered through a 100-µm nylon mesh to retain the epidermis. This epidermis, left on the mesh, was subsequently incubated in guard cell buffer 1 (5 mM KCl, 10 mM MES, and 50 µM CaCl_2_, pH 6.15 adjusted with Tris-base) under light conditions (~100 µmol m^−2^ s^−1^ photon flux density) for 2 h. Following this, the samples were treated with 10 μM ABA, 10 μM NS5806, or 10 μM UA49 for an additional 2 h in the light. To examine stomatal responses to chemicals during dark-to-light transitions, rosette leaves from 3 to 4-week-old plants were excised and incubated in guard cell buffer 2 (50 mM KCl and 10 mM MES, pH 6.2 adjusted with KOH) overnight in the dark. Epidermal fragments were obtained using a blender and incubated in fresh guard cell buffer 2 supplemented with 10 μM ABA, 10 μM NS5806, or 10 μM UA49 for 3 h under light conditions. The final concentration of DMSO was 0.2%. The width of the stomatal pore was measured under a bright field microscope, using Image J (http://imagej.nih.gov/ij/).

### Primary root length test

Col-0 seeds were placed into a 1.5 ml tube, and 200 μL of 0.1% Tween 20 were added. Then, 200 μl of sodium hypochlorite were added, and the seeds were suspended for 2 min (0.1% Tween 20: sodium hypochlorite = 1:1). The tube was centrifuged to pellet the seeds, and the supernatant was removed using a pipette. The seeds were resuspended with 1 mL of sterile water, and the liquid was discarded (wash). The washing process was repeated three times. The sterilized seeds were sown onto 1/2 MS agar medium with 0.4% Gellan gum, and the plate was wrapped in aluminum foil to keep it in the dark for 3 days of stratification at 4 °C. The plate was then transferred to a growth chamber set at 22 °C, with 16 h of light and 8 h of dark conditions, and the plates were placed vertically to allow the seeds to germinate and seedlings to grow. On the third day after germination, the seedlings were transferred to 1/2 MS agar medium containing 10 µM ABA, NS5806, or UA49. The final DMSO concentration was 0.1%. The plates were again placed vertically in the growth chamber for 4 days of growth, and then the seedlings were photographed. The root lengths were measured using Image J (http://imagej.nih.gov/ij/).

### Seed germination test

The seeds were resuspended with 1 mL of sterile water, and the liquid was discarded (wash). The sterilized seeds were sown onto 1/2 MS agar medium with 0.4% Gellan gum containing 10 µM ABA, NS5806 or UA49. The final DMSO concentration was 0.1%. The agar plate was wrapped in aluminum foil to keep it in the dark for 3 days of stratification at 4 °C. After that, the plate was transferred to a growth chamber set at 22 °C, with 16 h of light and 8 h of dark conditions. After 3 days, germinated seeds were counted.

### Immunohistochemical detection of PM H^+^-ATPase

Immunohistochemical staining was performed according to a previously described method with slight modifications^28^. In brief, epidermal tissues isolated from 4 to 5-week-old Col-0 plants were suspended in basal buffer (5 mm MES-1,3-bis[tris(hydroxymethyl)methylamino] propane [pH 6.5], 50 mm KCl, and 0.1 mm CaCl_2_) containing 10 μM ABA, NS5806, or UA49. The final concentration of DMSO was 0.1%. Then the epidermal tissues were illuminated with red light (50 µmol m^–2^ s^–1^) for 20 min, after which a blue light pulse (10 µmol m^–2^ s^–1^) was superimposed on the red light for 2.5 min. The epidermal tissues were fixed just before or after blue light illumination. The phosphorylation status of the penultimate residue, Thr (pen-Thr), of the PM H^+^-ATPase and the amount of PM H^+^-ATPase were detected using specific antibodies, anti-pen-pThr and anti-H^+^-ATPase^58^. Fluorescence from the Alexa Fluor488-labeled secondary antibodies was detected with a fluorescence microscope. Fluorescence signal intensities were quantified using ImageJ software (http://imagej.nih.gov/ij/).

### Confocal microscopy and image analysis of the cytoskeleton

To observe the actin cytoskeleton and microtubules, we used transgenic *A. thaliana* plants stably expressing GFP-ABD2 under the control of the CaMV35S promoter^59,60^ and VisGreen-TUB6 under the control of the TUB6 promoter^61,62^. Seeds were sown in Jiffy-7 Pots (Sakata Seed Corp., Yokohama, Japan) and incubated in a growth chamber at 23.5 °C under a 16 h light/8 h dark cycle. The light source was an 86.2 μmol m^−2^ s^−1^ light-emitting diode (Plantflec, LH-241PFP-S; NK System, Tokyo, Japan). The above-ground parts of the 14-day-old *A. thaliana* seedlings were detached and immediately immersed in a buffer containing 0.1% DMSO (mock), 10 μM ABA, NS5806, or UA49 for either 5 or 120 min. Subsequently, the abaxial side of the cotyledons was observed using confocal microscopy. Serial optical sections of guard cells were obtained at 0.5 μm intervals using a fluorescence microscope (IX-70; Olympus, Tokyo, Japan) equipped with a CSU-X1 scanning head (Yokogawa, Tokyo, Japan), a 100× objective lens (UPlanSApo100X, Evident, Tokyo, Japan), and a scientific complementary metal-oxide-semiconductor camera (Prime 95B; Teledyne Photometrics, Tucson, AZ, USA). GFP fluorescence was excited with a 488-nm laser and detected through a 510–550 nm band-pass filter. The skewness of the intensity distribution, an indicator of cytoskeleton bundling, was measured using skeletonized serial optical sections^59,63^.

### RNAseq analysis

Three- to 4-week-old Col-0 plants were treated for 3 h with 0.1% DMSO, 10 µM ABA, NS5806, or UA49 as described above in the drought stress test section and separated into shoot and root. Three independent biological samples for each treatment were harvested and snap-frozen in liquid nitrogen. Total RNA was extracted using a NucleoSpin RNA Plant (MACHEREY-NAGEL). Complementary DNA libraries were constructed from total RNA using the NEBNext Ultra II RNA Library Prep Kit for Illumina (New England Biolabs) and sequenced with a NextSeq 550 system (Illumina). Obtained reads were aligned to the Arabidopsis reference genome (TAIR10) using the RNA-Seq Alignment tool of Illumina BaseSpace. Normalization of read counts and statistical analysis were performed using the Bioconductor package edgeR^64^ in the web tool Degust ver. 3.1 (http://degust.erc.monash.edu); Low-expression genes were filtered out based on the criterion that their count-per-million (CPM) exceeded 0.5 in at least two samples. GO term analysis were performed with Shiny GO 0.80 (http://bioinformatics.sdstate.edu/go/), and graphs were created with R and Rstudio software (https://posit.co/download/rstudio-desktop/).

### PP2C enzyme assay

PP2C phosphatase assays were performed as described previously^65^ with some modifications. Briefly, PYLs and HAB1 were expressed in *E. coli* and purified by affinity column chromatography. Purified proteins were preincubated in 80 µL of a buffer (TBS, pH 7.5) containing 1.25 mM MnCl_2_ and test compound (5 µM ABA or 50 µM NS5806) at 22 °C for 30 min. After adding 20 µL of substrate buffer (165 mM Tris-acetate, pH 7.9, 330 mM potassium acetate, and 25 mM *p*NPP), reactions were immediately monitored for hydrolysis of *p*NPP at 405 nm. The reactions contained 600 nM HAB1 and 600 nM (PYR1, PYL1–6, and PYL10) or 1200 nM (PYL8 and PYL9) PYL proteins.

### Fluorescence microscopy to monitor cytosolic potassium and calcium dynamics

The Arabidopsis YC3.6 and lc-LysM GEPII 1.0 indicator lines were analyzed with two microscopy setups. Setup 1: Nikon Ti-E inverted fluorescence wide field microscope (Nikon, Tokyo, Japan) with a CFI PLAN APO 20 × VC dry objective for guard cell and root cell imaging. Excitation light at 440 nm (436/20 nm) was produced by a fluorescent lamp (Prior Lumen 200 PRO, Prior Scientific Inc., Rockland, MA, USA) set to 20%. Images were collected with a Hamamatsu Dual CCD camera (ORCA-D2, Hamamatsu Photonics, Hamamatsu City, Japan). The FRET CFP/YFP optical block A11400-03 (emission 1: 483/32 nm for CFP and emission 2: 542/27 nm for FRET) with a dichroic 510-nm mirror (Hamamatsu Photonics) was used for simultaneous CFP and cpVenus acquisitions. Exposure time was 200 ms with 4 × 4 CCD binning. Images were acquired every 5 s. Filters and dichroic mirrors were purchased from Chroma Technology. The NIS-Element software (Nikon) was used as a platform to control the microscope, illuminator, camera, and post-acquisition analyses. The same setup was used to perform experiments with plants expressing the ER-GCaMP6-210. Settings were identical to those reported in Grenzi et al. (2025)^66^, aside from the excitation light, 470 nm, that was produced by the CoolLED pE4000 illuminator (https://www.coolled.com/products/pe-4000/). The LED power was set to 20%, and the ER-GCaMP6-210 fluorescence emission was collected at 505-530 nm. Exposure time was set to 300 ms with 2 × 2 CCD binning. Images were acquired every 5 s.

Setup 2: Olympus IX73 inverted fluorescence microscope (Evident, Tokyo, Japan) with UPLXAPO 40XO oil objective for guard cell imaging. This system was connected to a Yokogawa spinning disk confocal unit CSU-W1 (Yokogawa Electric Corporation, Tokyo, Japan). Excitation light at 445 nm was generated by a laser (OBIS LX, Coherent Corporation, Tokyo, Japan) set to 85%. Images were collected with two Hamamatsu CMOS cameras (ORCA-Flash 4.0, Hamamatsu Photonics, Hamamatsu City, Japan). For simultaneous CFP and cpVenus (FRET) detection, a 514 LP dichroic mirror and two band-pass filters (482/35 nm and 542/27 nm) were used. The exposure time was 500 ms with 4 × 4 binning. Images were acquired every 5 s. Filters and dichroic mirrors were purchased from Semrock. The cellSens software was used as a platform to control the microscope, illuminator, camera, and post-acquisition analyses.

Time-lapse recordings were analyzed using the FIJI platform^67^. Fluorescence intensity measurements were conducted over specific regions of interest (ROI) corresponding to single guard cells or root tips. The emitted signals from cpVenus and CFP within these ROIs were utilized to compute the ratio (*R*) (cpVenus/CFP), which was plotted against time. These ratios were then normalized to the initial ratio (*R*_0_) and represented as Δ*R*/*R*_0_ against time. Before the ratio calculation, background subtraction was independently conducted for both fluorescence emission wavelengths. For ER-GCaMP6-210 the fluorescence intensity of each ROI was background subtracted and then normalized to its baseline as (*F* − *F*_0_)/*F*_0_ (∆*F*/*F*_0_), and plotted over time.

### Analyses of cytosolic calcium and potassium dynamics in guard cells

We used *Arabidopsis thaliana* plants expressing the Yellow Cameleon YC3.6 Ca^2+^ indicator^68^ or, lc-LysM GEPII 1.0^27^ fused to a cytosolic localization signal^26,57^, and the ER-GCaMP6-210^41^. Guard cells were imaged following the protocol outlined by Behera and Kudla^37^. In brief, small leaf discs from 3 to 4-week-old plants were glued to a cover slip using either Hollister 7730 medical adhesive (Hollister Incorporated, Libertyville, IL, USA) or Cemedine Super XG (Cemedine Co., Ltd., Japan). The upper cell layers were gently removed with a razor blade, leaving strips of the abaxial epidermis on the cover slip. For experiments assessing the effects of NS5806/UA49 on guard cell cytosolic K^+^ or Ca^2+^ concentrations, epidermal strips were incubated for 3 h in a buffer solution (5 mM KCl, 10 mM MES, and 50 µM CaCl_2_, pH 6.15 adjusted with Tris-base) in the growth chamber, under light to induce stomatal opening.

Subsequently, epidermal strips were carefully moved to an open-top chamber filled with the same buffer solution and mounted on the stage of a wide-field Nikon Ti-E microscope or Olympus IX73 microscope. The epidermal strips were imaged continuously for 10 min before and 30 min after the addition of NS5806/UA49 with 5 s interval. NS5806/UA49 were prepared as 100 µM stock solutions in dimethyl sulfoxide (DMSO) and directly added to the imaging solution to a final concentration of 10 µM. The final DMSO concentration in the experimental setup was maintained at 0.2%. For experiments to confirm Ca^2+^ influx from outside the cell membrane, 3 h after stomatal opening induction, the epidermal strips were washed 3 times with 1 mM EGTA for 5 min under lights to chelate the free Ca^2+^ in the apoplast. Epidermal strips were imaged continuously for 10 min before the addition of NS5806/UA49 to the imaging solution in the presence of 1 mM EGTA. Half of the solution filling the chamber was replaced with fresh NS5806/UA49 solution without EGTA at 10 min and 20 min after the initial addition of NS5806/UA49. The final DMSO concentration was 0.2% throughout the experiment.

### Statistical analysis

Tests for differences between two groups were performed using two-tailed Student’s *t* test. Multiple-group comparison was performed by one-way ANOVA with Tukey–Kramer test. Excel (Microsoft), CoStat (CoHort) and GraphPad Prism 8.4.3 (GraphPad) were used for these statistical analyses. *p-*values were set as described in the respective figure legends. Outliers were excluded using the ROUT method (*Q* = 1%).

### Reporting summary

Further information on research design is available in the Nature Portfolio Reporting Summary linked to this article.

## Supplementary information

Supplementary Information

Description of Additional Supplementary Files

Supplementary Movie 1

Supplementary Movie 2

Supplementary Movie 3

Reporting Summary

Transparent Peer Review File

## Source data

Source Data

## Data Availability

The authors declare that all data supporting the findings of this study are available within this article, Source Data file, and Supplementary Information file. RNA-seq data have been deposited in the DDBJ Sequence Read Archive at the DNA Data Bank (http://www.ddbj.nig.ac.jp/) with the BioProject accession number PRJDB20282. The collection of 135 commercially-available animal channel regulators was provided to us by Sumitomo Chemical Co., Ltd. and each regulator is commercially available. Source data are provided with this paper.
