# Supplementary material for: Synthetic ion channel inhibitors enhance plant drought tolerance
Source: Nat Commun. 2026 Jul 27;17:7257. doi: 10.1038/s41467-026-75894-w (PMC13408460; doi:10.1038/s41467-026-75894-w)
Supplement: Supplementary file 1 — Supplementary Information [file 41467_2026_75894_MOESM1_ESM.pdf]

Supplementary Figure 1

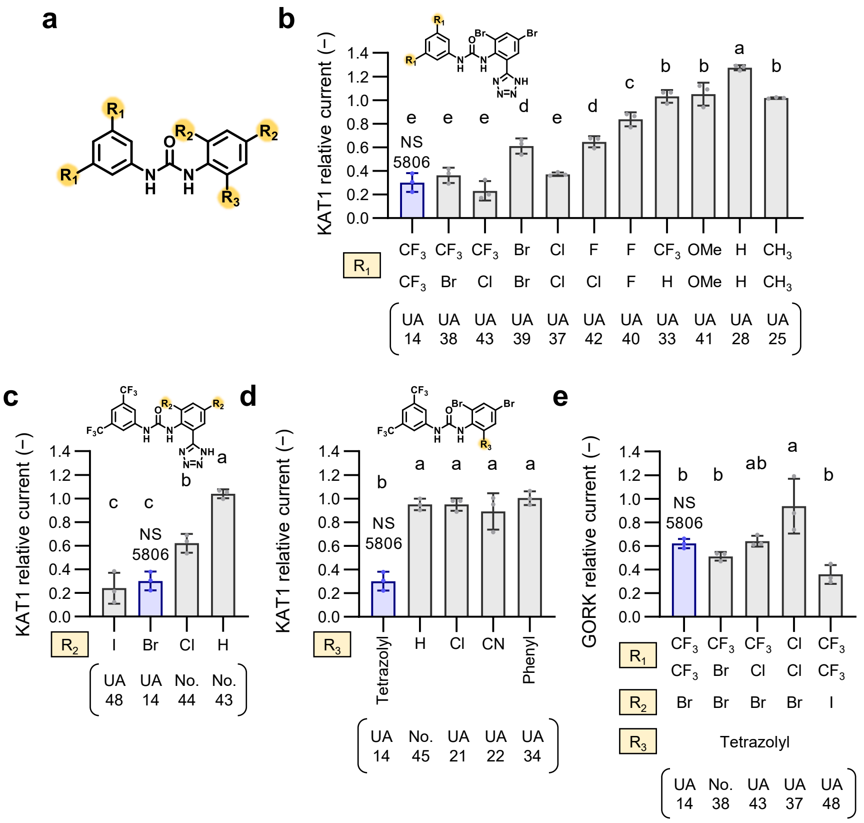

Supplementary Fig. 1: Activities of structural analogs of NS5806.
a to **d**, NS5806 was examined for structural specificity by dividing it into three parts, R_1, R_2, and R_3, as shown in the figure in **a** with Two-electrode voltage clamp. KAT1 current data are shown; R_1: electronegativity in **b**, R_2: types of halogens in **c**, and R_3: water solubility in **d** by KAT1 recording using two-electrode voltage clamp respectively. Two-electrode voltage clamps were carried out for KAT1 with or without 30 µM for each compound in the external buffer, and relative currents were plotted (n = 3, mean ± SD). Bars marked with different letters are significantly different ($p < 0.05$) by one-way ANOVA with Tukey–Kramer test. The current value was collected at the end of the pulse at -170 mV. The data of NS5806 in **b**, **c**, and **d** are the same. **e**, Effect on GORK current in analogous compounds of NS5806. Two-electrode voltage clamps were carried out for GORK with or without 30 µM for each compound in the bath solution, and relative currents were plotted (n = 3, mean ± SD).

Supplementary Figure 2

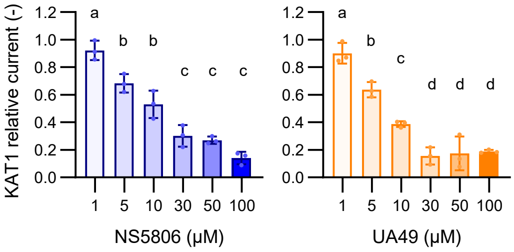

Supplementary Fig. 2: NS5806 and UA49 inhibit K^+ channels in a dose-dependent manner.
Dose dependency test of NS5806 and UA49 on KAT1. Two-electrode voltage clamp was performed using *Xenopus laevis* oocytes for each channel with or without NS5806 or UA49 in the bath solution, and relative currents were plotted ($n = 3$, mean ± SD). The current value was collected at the end of the pulse at −170 mV. Bars marked with different letters are significantly different ($p < 0.05$) by one-way ANOVA with Tukey–Kramer test.

Supplementary Figure 3

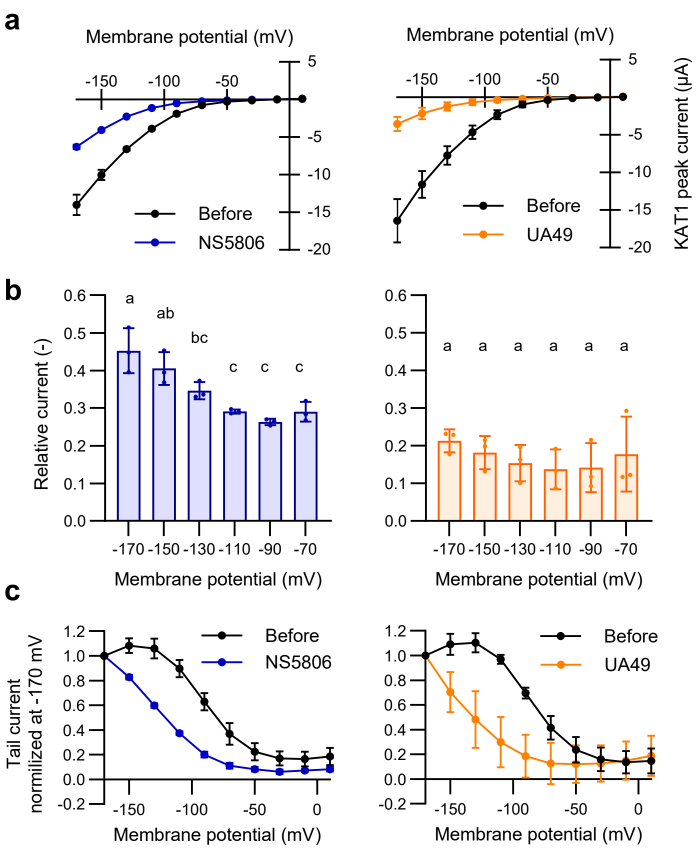

Supplementary Fig. 3: Reanalyzed data of KAT1 current obtained by two electrode voltage clamp in Fig. 1c.
a, I-V diagram for peak current. The current value was collected at the end of the pulse. **b**, Relative peak currents at each pulse following treatment with 30 μM NS5806 or UA49. Bars marked with different letters are significantly different ($p < 0.05$) by one-way ANOVA with Tukey–Kramer test ($n = 3$, mean ± SD). **c**, I-V diagram for tail current. Current amplitudes were measured at 15 ms after stepping back to the holding voltage, and the values were plotted against the preceding pulse potentials.

Supplementary Figure 4

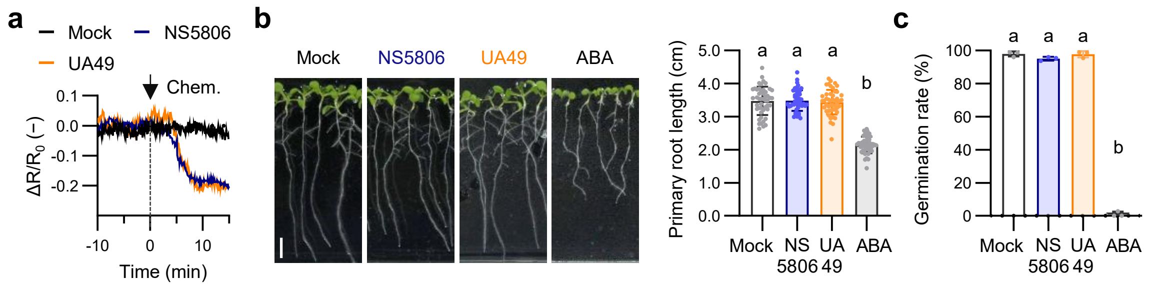

Supplementary Fig. 4: Effect of NS5806 and UA49 on Arabidopsis growth.
a, Time course data of representative normalized cpVenus/CFP ratios of lc-LysM GEPII 1.0. The leaf epidermal strips were treated with 0.2% DMSO (Mock), 10 µM NS5806, or UA49 at 0 min. **b**, Phenotype of *A. thaliana* root length for NS5806, UA49, and ABA. Three-day-old plants were transferred to 1/2MS (Murashige and Skoog) medium including 0.1% DMSO (Mock), 10 µM NS5806, UA49, and ABA and imaged after 4 days later. Scale bar = 5 µm. Primary root length was measured with Image J software. Data are shown as mean ± SD (n = 47-48). Bars marked with different letters are significantly different ($p < 0.05$) by one-way ANOVA with Tukey–Kramer test. **c**, The effect of NS5806 and UA49 for *A. thaliana* seed germination. 70-100 seeds were sow on 1/2MS medium including 0.1% DMSO (Mock), 10 µM NS5806, UA49, and ABA. The experiments were repeated 3 times. Data are shown as mean ± SD. Bars marked with different letters are significantly different ($p < 0.05$) by one-way ANOVA with Tukey–Kramer test.

Supplementary Figure 5

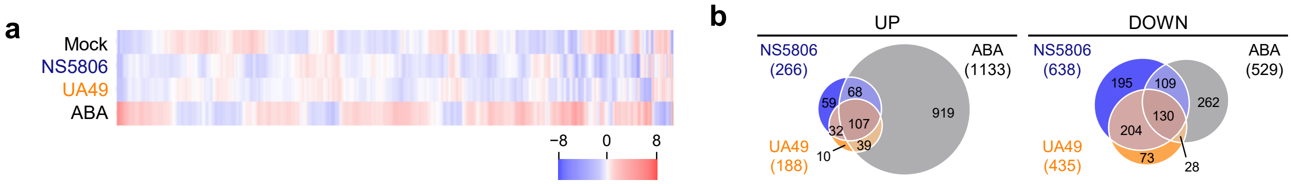

Supplementary Fig. 5: Gene expression analysis for NS5806 and UA49.
RNA expression profiles of *A. thaliana* 3 h after 0.1% DMSO (Mock), 10 µM NS5806, UA49, or ABA spraying on leaf adaxial side. Heatmap of differentially expressed genes between NS5806, UA49, or ABA vs. Mock is shown in **a**, where red is higher expression and blue is lower expression. Venn diagrams are shown in **b**. Three biological replicates, log2 fold change > 1 (UP) and <−1 (DOWN), edgeR; FDR < 0.01.

Supplementary Figure 6

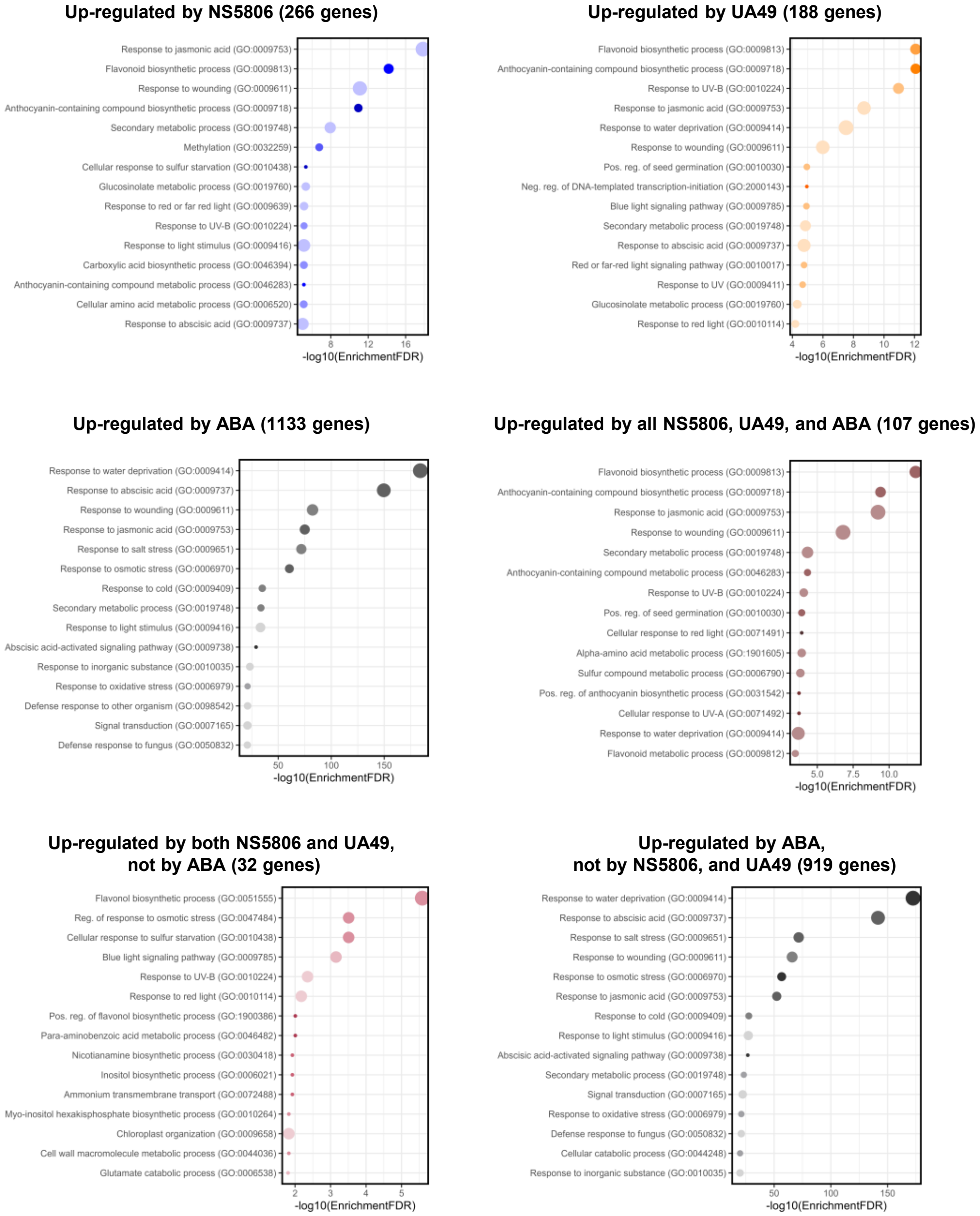

Supplementary Fig. 6: Gene ontology (GO) term analysis of upregulated genes by NS5806, UA49, or ABA.
GO term analysis were performed with Shiny GO 0.80 (http://bioinformatics.sdstate.edu/go/) using RNA expression profiles shown in Fig. 3f and g. Process: GO Biological process; Species: Arabidopsis thaliana araport11; FDR cutoff: 0.05; selected by FDR, sort by Fold Enrichment.

Supplementary Figure 7

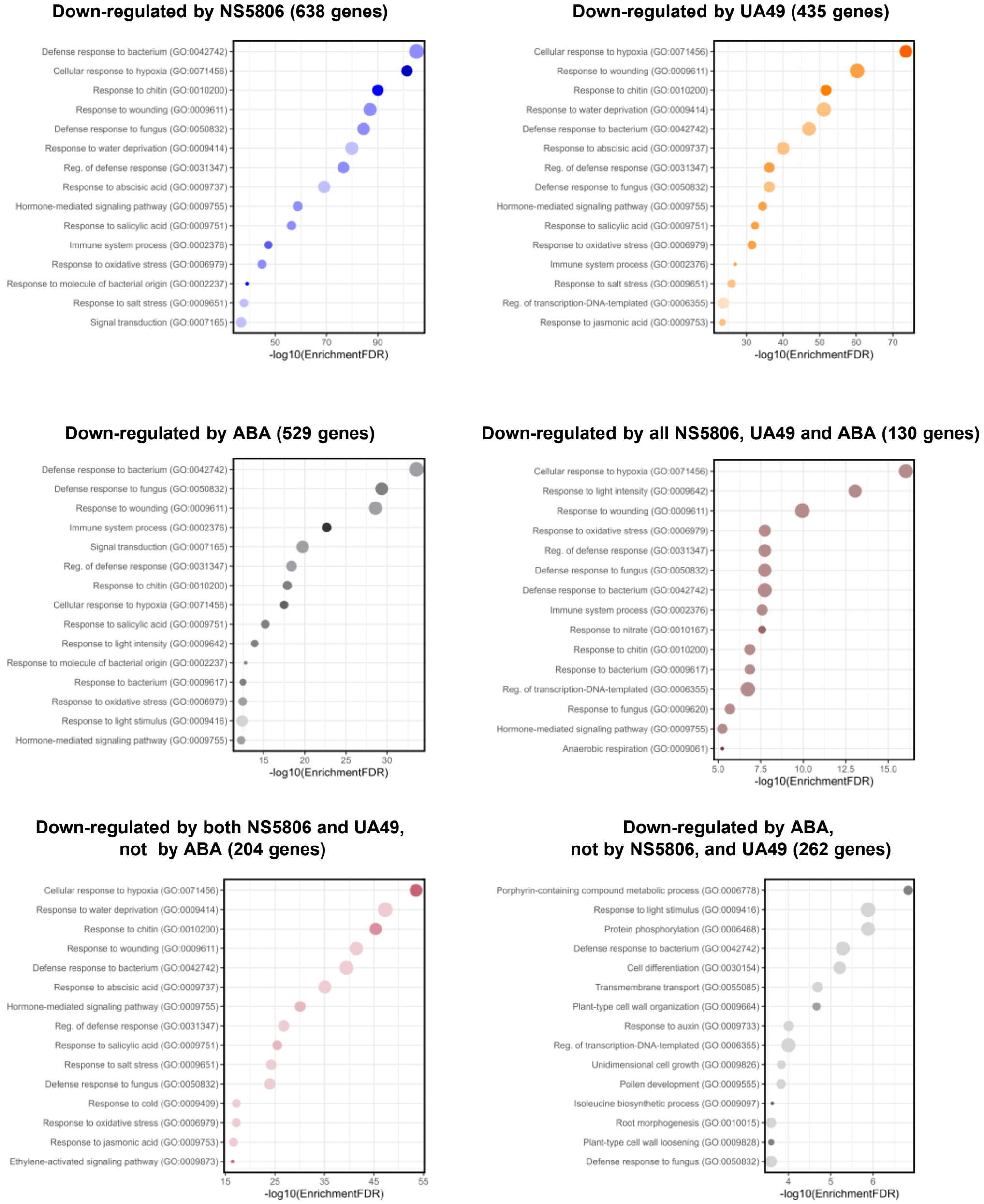

Supplementary Fig. 7: Gene ontology (GO) term analysis of downregulated genes by NS5806, UA49, or ABA.
GO term analysis were performed with Shiny GO 0.80 (http://bioinformatics.sdstate.edu/go/) using RNA expression profiles shown in Fig. 3f and g. Process: GO Biological process; Species: Arabidopsis thaliana araport11; FDR cutoff: 0.05; selected by FDR, sort by Fold Enrichment.

Supplementary Figure 8

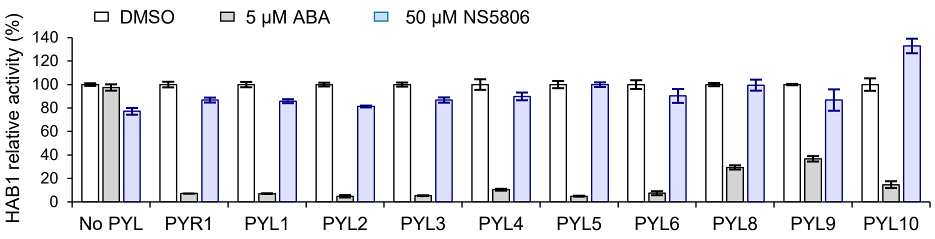

Supplementary Fig. 8: Effect of NS5806 on HAB1 inhibition by ABA receptors.
Chemical inhibition of HAB1 by various ABA receptors in the presence of 5 μM ABA or 50 μM NS5806. The concentration of each PYL was set at a molar ratio to HAB1 of 1:1 (PYR1, PYL1−6, and PYL10) or 2:1 (PYL8 and PYL9). HAB1 phosphatase activity was normalized to a control (DMSO-treated) value of 100% (*n* = 3, error bars represent SD) and is expressed as relative activity.

Supplementary Figure 9

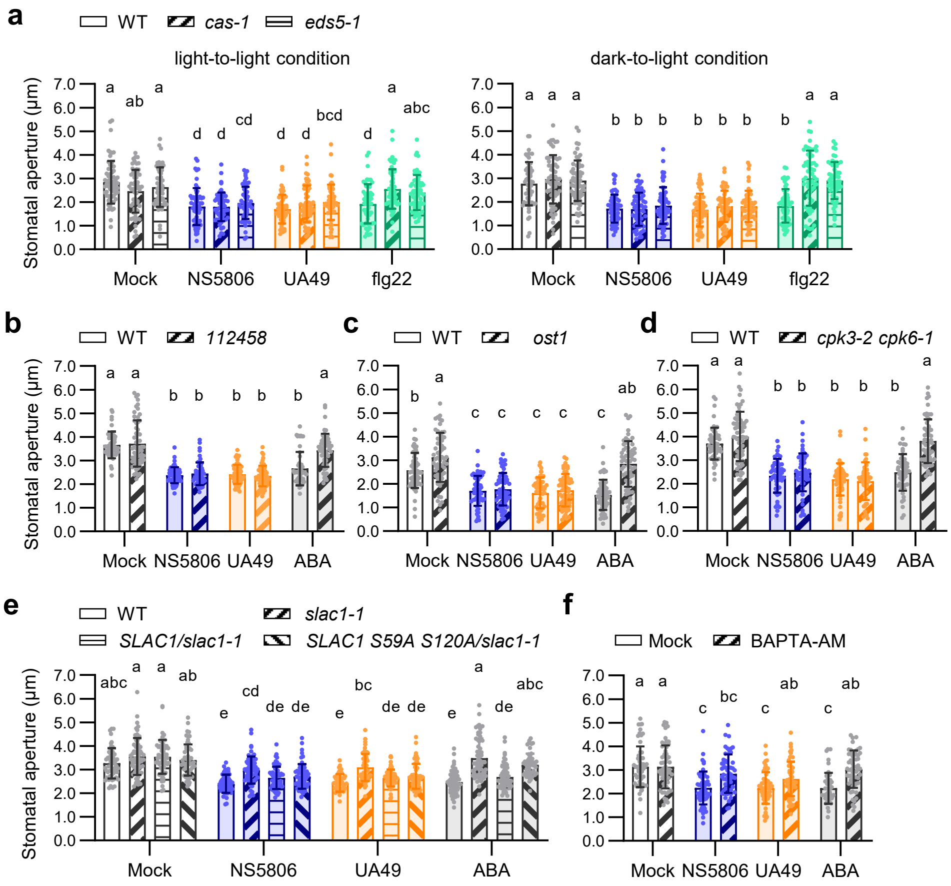

Supplementary Fig. 9: Stomatal responses to NS5806 or UA49.

The stomatal aperture was measured using *A. thaliana* mutants. For the light-to-light condition test, epidermal strips were pre-incubated for 2 h under light to induce stomatal opening and followed by a 2 h incubation under light with 0.2% DMSO (Mock), 10 µM NS5806, UA49, or ABA. For the dark-to-light condition test, epidermal strips were pre-incubated overnight under the dark to induce stomatal closing and followed by a 3 h incubation under light with 0.2% DMSO (Mock), 10 µM NS5806, UA49, or ABA. **a**, Characteristic factors in pathogen responses; *cas-1* and *eds5-1*. Stomatal response under the light-to-light transition (left, n = 58-60) and dark-to-light transition (right, n = 60). **b-f**, Stomatal response under dark-to-light transition. **b** and **c**, Characteristic genes in the ABA pathway; *pyr1 pyl1 ply2 pyl4 pyl5 pyl8* (*112458*) (**b**, n = 54-60) and *ost1* (**c**, n = 60). **d**, Ca^{2+}-dependent protein kinases; *cpk3-2 cpk6-1* (n = 59). **e**, The anion channel required for stomatal closure; *slac1*, *SLAC1/slac1-1* and *SLAC1 S59A S120A/slac1-1* (n = 58-60). **f**, Requirement of cytosolic Ca^{2+} in stomatal closure; WT with BAPTA-AM (n = 60). Data are shown as mean ± SD. Bars marked with different letters are significantly different ($p < 0.05$) by one-way ANOVA with Tukey–Kramer test.

Supplementary Figure 10

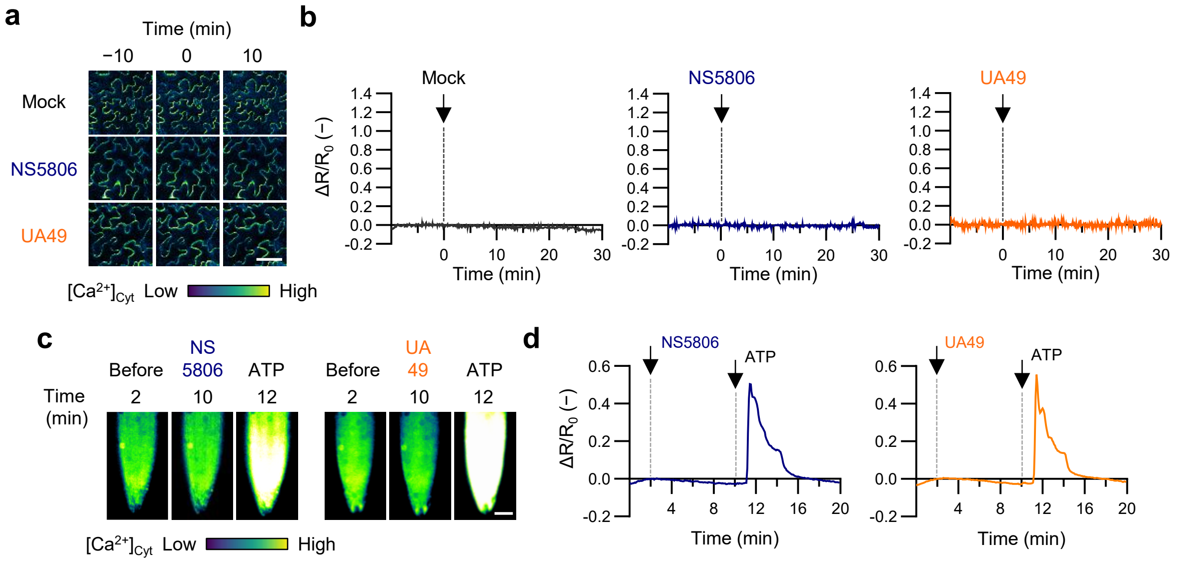

Supplementary Fig. 10: Tissue specificity of cytosolic Ca^{2+} dynamics triggered by NS5806 and UA49.

a and **b**, The changes of cytosolic Ca^{2+} levels in epidermal pavement cells in response to NS5806/UA49 treatment. **a**, Representative false-color images displaying the cpVenus/CFP emission ratios of NES-YC3.6. Data were collected for 40 min, with 10 µM NS5806/UA49 applied to the imaging solution 10 min after the onset (defined as 0 min on the labels). Scale bar = 50 µm. **b**, Representative time course of cytosolic Ca^{2+} level of epidermal pavement cells. Data were collected from 24 guard cells. **c** and **d**, The changes of cytosolic Ca^{2+} levels in root cells in response to NS5806/UA49 treatment. **c**, Representative false-color images displaying the cpVenus/CFP emission ratios of NES-YC3.6. Data were collected for 20 min, with 10 µM NS5806/UA49 applied to the imaging solution at 2 min and 1 mM ATP applied at 10 min after the onset. **d**, Representative time course of cytosolic Ca^{2+} level of root cells. Data were collected from 3 plants. Scale bar = 50 µm.

Supplementary Figure 11

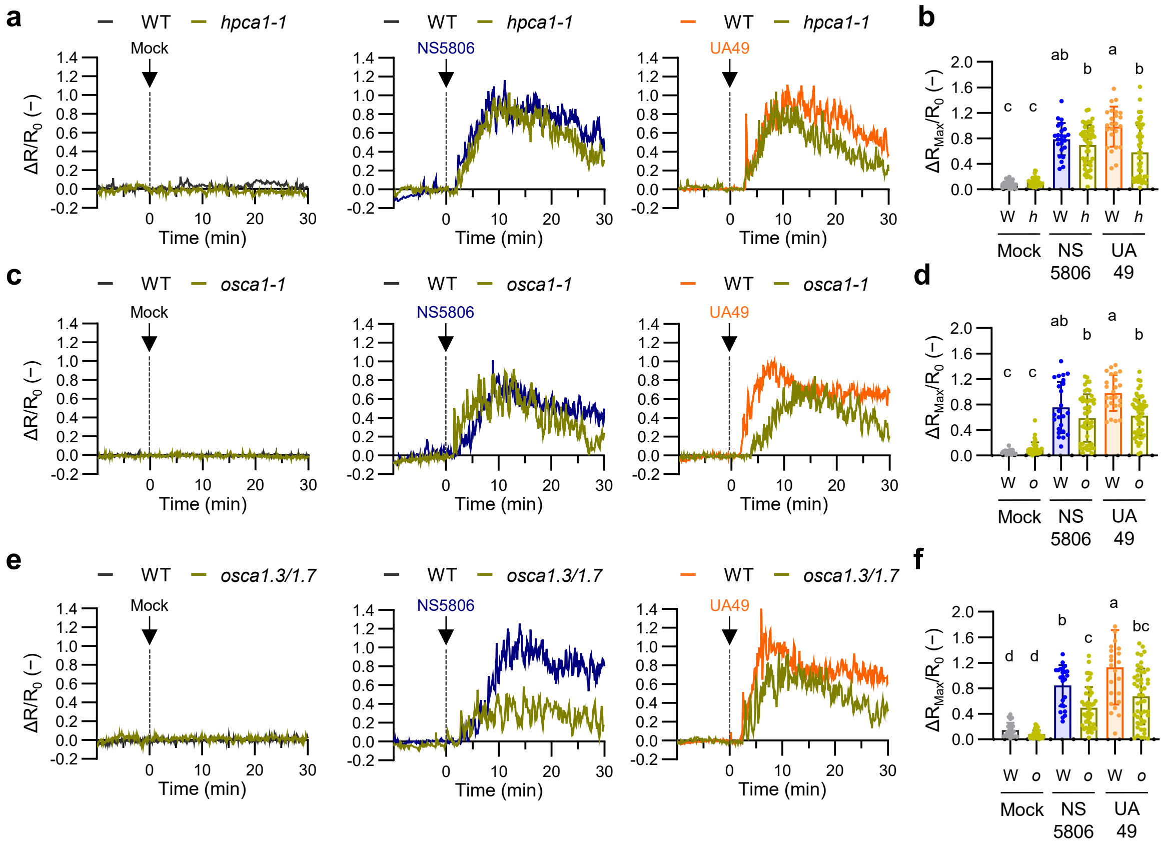

Supplementary Fig. 11: Cytosolic Ca^{2+} imaging of *Arabidopsis* calcium channel-deficient mutants expressing YC3.6 upon treatment with NS5806 or UA49.

Data were collected in the same way as Fig 5a, b. Guard cells were treated with 10 µM NS5806/UA49 at 0 min as indicated in the graphs. **a** and **b**, *hpca1-1* (n = 24-48), **c** and **d**, *osca1-1* (n = 22-48), **e** and **f**, *osca1.3/1.7* (n = 24-48). **a**, **c**, and **e** show the representative time course. **b**, **d**, and **f** show the peak cpVenus/CFP ratios as $\Delta R/R_0$ maximum (mean ± SD). Bars marked with different letters are significantly different ($p < 0.05$) by one-way ANOVA with Tukey–Kramer test.

Supplementary Figure 12

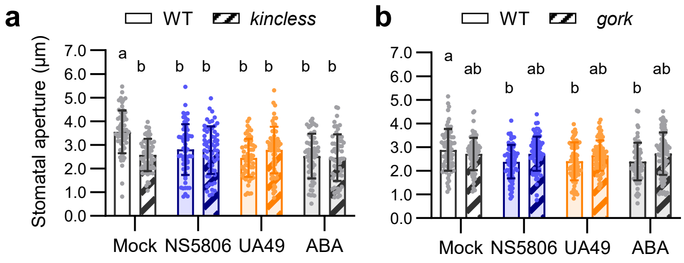

Supplementary Fig. 12: Stomatal responses by NS5806 and UA49 treatments under light-to-light condition (test for inducement of stomatal closure).

The stomatal aperture was measured using *A. thaliana* mutants. For the light-to-light condition test, epidermal strips were pre-incubated for 2 h under light to induce stomatal opening and followed by a 2 h incubation under light with 0.2% DMSO (Mock), 10 µM NS5806, UA49, or ABA. **a**, *kincless* (n = 60). **b**, *gork* (n = 80). Data are shown as mean ± SD. Bars marked with different letters are significantly different ($p < 0.05$) by one-way ANOVA with Tukey–Kramer test.

Supplementary Information

^{1}H-, ^{13}C-, ^{19}F-NMR spectra were recorded on a Bruker Avance (400 MHz) and JEOL ECS (400 MHz). ^{19}F-NMR spectra were recorded with trifluoroacetic acid as an external standard ($\delta -78.5$). The following abbreviations (or combinations thereof) were used to explain multiplicities: s = singlet, d = doublet, t = triplet, q = quartet, m = multiplet. Melting points were determined with a Yanagimoto micro melting point apparatus without correction. Infrared (IR) spectra were recorded on a JASCO FT/IR-410 spectrometer. Mass spectra were measured on a SHIMAZU LCMS-IT-TOF spectrometer.

Procedure for synthesis of 1-[3-chloro-5-(trifluoromethyl)phenyl]-3-[3,6-diiodo-2-(1*H*-tetrazol-5yl)phenyl]urea UA49

In a three-necked flask were placed I_2 (10.6 g, 42 mmol), Ag_2SO_4 (13.1 g, 42 mmol), and 2-aminobenzonitrile (2.4 g, 20 mmol) in EtOH (100 mL) under an argon atmosphere, and the mixture was heated at reflux for 4 h in an oil bath. The solid was removed by filtration and washed with ethyl acetate. After removing solvent of the filtrate under reduced pressure, the residue was dissolved in THF (100 mL), 1N NaOH aq. was added, and the mixture was stirred for 10 min. THF was removed under reduced pressure, and the aqueous layer was extracted with ethyl acetate (100 mLx4). The combined organic extracts were dried with $MgSO_4$ and concentrated under reduced pressure. The residue was recrystallized from EtOH to obtain 2-amino-3,5-diiodobenzonitrile (5.0 g, 68%).

In a two-necked flask were placed 2-amino-3,5-diiodobenzonitrile (2.0 g, 5.4 mmol), tetrabutylammonium fluoride trihydrate (850 mg, 2.7 mmol) and trimethylsilyl azide (1.05 mL, 924 mg, 8.0 mmol) under an argon atmosphere, and the mixture was heated at 85 °C for 2 h. The mixture was dissolved in ethyl acetate and washed with 1N HCl (20 mLx3). The organic layer was dried with $MgSO_4$ and concentrated under reduced pressure. The residue was washed with CH_2Cl_2 (300 mL) giving 2,4-diiodo-6-(1*H*-tetrazol-5-yl)aniline (1.7 g, 78%).

Solution **A**: In a two-necked flask was placed triphosgen (77.1 mg, 0.26 mmol) in CH_2Cl_2 (1.5 mL) under an argon atmosphere, and the solution was stirred at 0 °C. Then the solution of 3-iodo-5-(trifluoromethyl)aniline (195.6 mg, 1.0 mmol) and triethylamine (0.1 mL) in CH_2Cl_2 (1.0 mL) was added dropwise, and the mixture stirred at 0 °C for 5 min.
In a two-necked flask were placed 2,4-diiodo-6-(1*H*-tetrazol-5-yl)aniline (289.1 mg, 0.7 mmol), potassium carbonate (117.5 mg, 0.85 mmol) in THF (3 mL) under an argon atmosphere and solution **A** was added dropwise , the mixture was stirred at 35 °C for 11 h. Then solvent was removed under reduced pressure, and the residue was purified by flash column chromatography an silica gel (eluent; AcOEt/MeOH = 9/1) giving 1-[3-chloro-5-(trifluoromethyl)phenyl]-3-[3,6-diiodo-2-(1*H*-tetrazol-5yl)phenyl]urea **UA49** (140.7 mg, 32%). The scheme is shown in the Supplementary Fig.13.

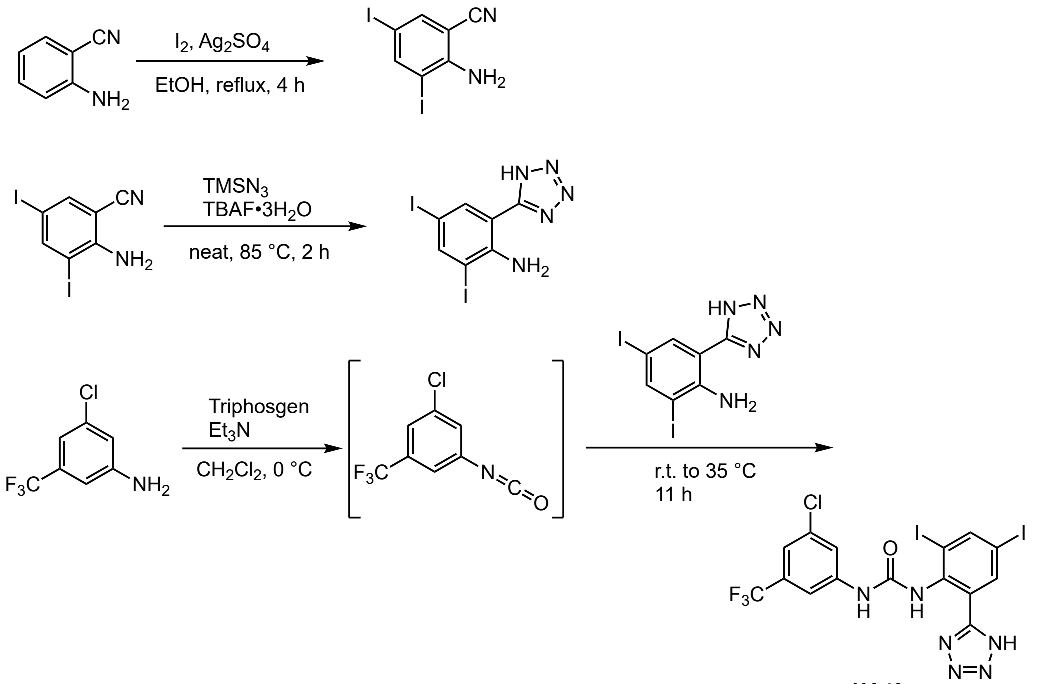

Supplementary Fig. 13: Synthesis of *N*-[3-chloro-5-(trifluoromethyl)phenyl]-*N'*-[3,6-diiodo-2-(1*H*-tetrazol-5yl)phenyl]urea **UA49.**

_N_-[3-Chloro-5-(trifluoromethyl)phenyl]-_N'_-[2,4-diiodo-6-(2_H_-tetrazol-5yl)phenyl]urea UA49

Pale pink solid. M.p. 216.9-217.8 °C (Toluene). ^{1}H-NMR (400 MHz, DMSO-d$_6$) δ 9.67 (1H, bs), 8.71 (1H, s), 8.42 (1H, d, J = 2.0 Hz), 8.20 (1H, d, J = 2.0 Hz), 7.74 (1H, s), 7.72 (1H, s), 7.38 (1H, s). ^{13}C-NMR (100 MHz, DMSO-d$_6$) δ 154.0, 152.2, 147.9, 142.2, 137.8, 137.6, 134.2, 131.1 (q, J = 32.6 Hz), 125.9, 123.3 (q, J = 272.0 Hz), 120.9, 117.8 (d, J = 3.4 Hz), 112.9 (d, J = 3.8 Hz), 104.0, 92.8. ^{19}F-NMR (376 MHz, DMSO-d$_6$) δ −61.7. IR (KBr) ν 3315, 1658, 1600, 1557, 1461, 1337, 1174, 1131 cm^{-1}. HRMS (ESI) Calcd for $C_{15}H_9ON_6F_3ClI_2$: 634.8562. Found: 634.8527.

Procedure for synthesis of _N_-[3,5-bis(trifluoromethyl)phenyl]-_N'_-[2,4-dibromo-6-(2_H_-tetrazol-5yl)phenyl]urea NS5806 (UA14)

In a three-necked flask, bromine (110 mmol, 5.6 mL) was added dropwise to 2-aminobenzonitrile (50 mmol, 5.9 g) in an acetic acid solution (150 mL) under an argon atmosphere, and the mixture was stirred at room temperature for 5 hours. The reaction mixture was poured into ice water (200 mL), and the resulting solid was filtrated and washed with water. The residue was recrystallized from ethyl acetate to obtain 2-amino-3,5-dibromobenzonitrile (6.9 g, 50%).

In a two-necked flask were placed 2-amino-3,5-dibromobenzonitrile (2.75 g, 10.0 mmol), tetrabutylammonium fluoride trihydrate (1.58 g, 5.0 mmol) and trimethylsilyl azide (8.0 mmol, 1.05 mL) under an argon atmosphere, and the mixture was heated at 85 °C for 2 h. The mixture was dissolved in ethyl acetate and washed with 1N HCl (5 mLx3). The organic layer was dried with $MgSO_4$ and concentrated under reduced pressure. The residue was purified by flash column chromatography an silica gel (eluent; AcOEt/EtOH = 10/1) giving 2,4-dibromo-6-(1*H*-tetrazol-5-yl)aniline (1.4 g, 44%).

3,5-Di(trifluoromethyl)phenylisocyanate (0.5 mmol, 87.0 μL) was added dropwise to the THF (3.0 mL) solution of 2,4-dibromo-6-(1*H*-tetrazol-5-yl)aniline (157.5 mg, 0.5 mmol) and triethylamine (1.5 mL), and the mixture stirred at room temperature for 12 h. Then solvent was removed under reduced pressure, and the residue was purified by flash column chromatography an silica gel (eluent; AcOEt/Hexane = 1/1) giving *N*-[3,5-bis(trifluoromethyl)phenyl]-*N'*-[2,4-dibromo-6-(2*H*-tetrazol-5yl)phenyl]urea **NS5806** (**UA14**, 57.4 mg, 20%). The scheme is shown in the Supplementary Fig.14.

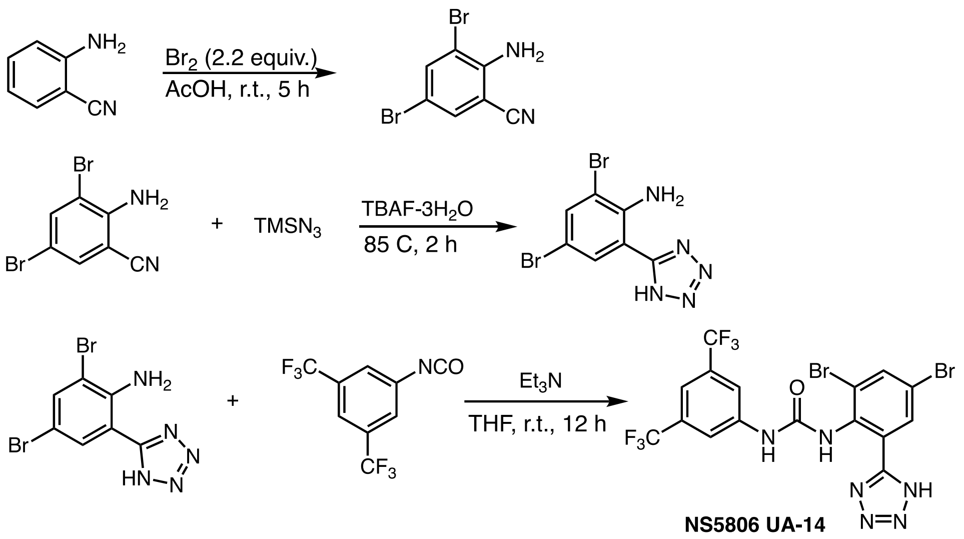

Supplementary Fig. 14: Synthesis of *N*-[3,5-bis(trifluoromethyl)phenyl]-N'-[2,4-dibromo-6-(2*H*-tetrazol-5yl)phenyl]urea **NS5806** (**UA14**)

***N*-[3,5-bis(trifluoromethyl)phenyl]-N'-[2,4-dibromo-6-(2*H*-tetrazol-5yl)phenyl]urea NS5806 (UA14)**

Coloreless solid. M.p. 219.0-220.0 °C (decomposed, AcOEt/Toluene = 2/1). ^{1}H-NMR (400 MHz, DMSO-d$_6$) δ9.82 (1H, bs), 8.72 (1H, s), 8.21 (d, J = 2.4 Hz), 8.10 (d, J = 2.4 Hz), 8.04 (2H, s), 7.61 (1H, s). ^{13}C-NMR (100 MHz, DMSO-d$_6$) δ1153.9, 152.3, 141.8, 136.8, 134.5, 131.6, 130.7 (q, J = 32.6 Hz), 126.5, 125.4, 123.3 (q, J = 271.4 Hz), 119.5, 117.8, 114.5. ^{19}F-NMR (376 MHz, DMSO-d$_6$) δ –61.5. IR (KBr) 3353, 1681, 1279. LRMS (FAB) *m/z* 576.9 ([**M**+H+4]$^+$), 574.9 ([**M**+H+2]$^+$), 572.9 ([**M**+H]$^+$). HRMS (FAB) Calcd for $C_{16}H_9Br_2F_6N_6O^+$ ([**M**+H]$^+$): 572.9109, Found: 572.9117.

***N*-[3-Trifluoromethyl-5-bromophenyl]-*N'*-[2,4-dibromo-6-(2*H*-tetrazol-5yl)phenyl]urea UA38**

Colorless solid. M.p. 190.0-190.1 °C (decomposed). ^{1}H-NMR (400 MHz, DMSO-d$_6$) δ 7.49 (1H, s), 7.81 (1H, d, J = 2.4 Hz), 7.86 (1H, s), 7.98 (1H, s), 8.22 (1H, d, J = 2.0 Hz), 10.3 (1H, s), 10. 4 (1H, s). ^{13}C-NMR (150 MHz, DMSO-d$_6$) δ 113.2, 118.8, 120.5, 122.3, 123.0 (q, J = 273.6 Hz), 123.8, 124.4, 126.9, 130.9, 131.2 (q, J = 31.7 Hz), 134.3, 135.9, 142.1, 152.1, 155.0. ^{19}F-NMR (376 MHz, DMSO-d$_6$) δ -61.7 (s, 3F). IR (KBr, cm^{-1}) 3328, 1657, 1560, 1456, 1334, 1130, 863. LRMS (FAB) m/z 588.9 ([M+H+6]$^+$), 586.9 ([M+H+4]$^+$), 584.9 ([M+H+2]$^+$), 582.9 ([M+H]$^+$); HRMS (FAB) Calcd for $C_{15}H_9Br_3F_3N_6O^+$ ([M+H]$^+$): 582.8340, Found 582.8346.

***N*-[3-Chloro-5-trifluorophenyl]-*N'*-[2,4-dibromo-6-(2*H*-tetrazol-5yl)phenyl]urea UA43**

Colorless solid. M.p. 215.0-216.0 °C (decomposed. Ethyl acetate:Toluene:Hexane = 1:1:1). ^{1}H-NMR (400 MHz, DMSO-d$_6$) δ 7.38 (1H, s), 7.71 (1H, s), 7.73 (1H, s), 8.08 (1H, d, J = 2.0 Hz), 8.20 (1H, d, J = 2.0 Hz), 8.69 (1H, s), 9.67 (1H, s). ^{13}C-NMR (150 MHz, DMSO-d$_6$) δ 112.9, 117.8, 119.3, 120.9, 123.2 (q, J = 273.5 Hz), 125.1, 126.3, 131.0 (q, J = 31.7 Hz), 131.5, 134.2, 134.5, 136.8, 142.0, 152.1, 153.9. ^{19}F-NMR (376 MHz, DMSO-d$_6$) δ -61.7 (s, 3F). IR (KBr, cm^{-1}) 3354, 3332, 3088, 1687, 1556, 1339, 1178, 1122, 871. LRMS (FAB) m/z 542.9 ([M+H+4]$^+$), 540.9 ([M+H+2]$^+$), 538.9 ([M+H]$^+$). HRMS (FAB): Calcd for $C_{15}H_9Br_2ClF_3N_6O^+$ ([M+H]$^+$) 538.8845, Found 538.8862.

***N*-[3,5-Di(bromo)phenyl]-*N'*-[2,4-di(bromo)-6-(2*H*-tetrazol-5yl)phenyl]urea UA39**

Colorless solid. M.p. 218.0-219.0 °C (decomposed). ^{1}H-NMR (400 MHz, DMSO-d_6) δ 7.37 (1H, t, J = 1.6 Hz), 7.59 (2H, d, J = 2.0 Hz), 8.07 (1H, d, J = 2.0 Hz), 8.21 (1H, d, J = 2.0 Hz), 8.62 (1H, bs), 9.46 (1H, bs). ^{13}C-NMR (150 MHz, DMSO-d_6) δ 119.2, 119.4, 122.3, 124.9, 126.1, 126.3, 131.5, 134.5, 136.7, 142.4, 151.9, 153.9. IR (KBr, cm^{-1}) 3353, 3074, 1675, 1582, 1539, 871. LRMS (FAB) m/z 600.8 ([M+H+8]$^+$), 598.8 ([M+H+6]$^+$), 596.8 ([M+H+4]$^+$), 594.8 ([M+H+2]$^+$), 592.8 ([M+H]$^+$); HRMS (FAB) Calcd for $C_{14}H_9Br_4N_6O^+$ ([M+H]$^+$): 592.7571, Found 592.7543.

***N*-[3,5-Di(chloro)phenyl]-*N'*-[2,4-di(bromo)-6-(2*H*-tetrazol-5yl)phenyl]urea UA37**

Colorless solid. M.p. 227.0-228.0 °C. ^{1}H-NMR (600 MHz, DMSO-d_6) δ 7.12-7.14 (1H, m), 7.41 (2H, d, J = 1.6 Hz), 8.08 (1H, bs), 8.17 (1H, s), 8.70 (1H, s), 9.51 (1H, s). ^{13}C-NMR (150 MHz, DMSO-d_6) δ 114.1, 116.1, 119.1, 121.0, 124.7, 126.4, 131.4, 134.0, 134.5, 136.5, 142.1, 151.9. IR (neat, cm^{-1}) 3297, 3072, 2987, 1700, 1591, 1543, 1445, 1437, 1263, 1215. LRMS (FAB) 505 (M+H)$^+$. HRMS (FAB-EB) (M+H)$^+$ Calcd for $C_{14}H_9Br_2Cl_2N_6O^+$: 504.8582, Found 504.8570.

***N*-(3-Chloro-5-fluorophenyl)-*N'*-[2,4-di(bromo)-6-(2*H*-tetrazol-5yl)phenyl]urea UA42**

Colorless solid. M.p. 179.0-180.0 °C (Ethyl acetate:Hexane = 1:1). ^{1}H-NMR (400 MHz, DMSO-d_6) δ 6.97 (1H, dt, J = 8.4, 2.0 Hz), 7.27 (1H, dt, J = 11.6, 2.0 Hz), 7.35 (1H, t, J = 2.0 Hz), 7.96 (1H, d, J = 2.4 Hz), 8.15 (1H, d, J = 2.4 Hz), 9.60 (1H, bs), 10.01 (1H, bs). ^{13}C-NMR (150 MHz, DMSO-d_6) δ 103.4 (d, J = 26.0 Hz), 108.8 (d, J = 25.8 Hz), 113.5, 118.0, 123.4, 127.5, 130.1, 133.9 (d, J = 8.7 Hz), 134.2, 134.7, 142.6 (d, J = 13.1 Hz), 151.9, 156.3, 162.3 (d, J = 164.1 Hz). ^{19}F-NMR (376 MHz, DMSO-d_6) δ -110.3 (1F, t, J = 10.2 Hz). IR (KBr, cm^{-1}) 3358, 2923, 1681, 1606, 1557, 1426, 1210. LRMS (FAB) m/z 492.9 ([M+H+4]$^+$), 490.9 ([M+H+2]$^+$), 488.9 ([M+H]$^+$). HRMS (FAB) Calcd for $C_{14}H_9Br_2ClFN_6O^+$ ([M+H]$^+$), 488.8877, Found 488.8829.

***N*-[3,5-Di(fluoro)phenyl]-*N'*-[2,4-di(bromo)-6-(2*H*-tetrazol-5yl)phenyl]urea UA40**

Colorless solid. M.p. 215.5-216.5 °C (decomposed. Ethyl acetate:Toluene:Hexane = 1:1:1). ^{1}H-NMR (400 MHz, DMSO-d_6) δ 6.77 (1H, tt, J = 9.2 Hz), 7.03-7.09 (2H, m), 8.07 (1H, d, J = 2.4 Hz), 8.20 (1H, d, J = 2.4 Hz), 8.61 (1H, s), 9.54 (1H, s). ^{13}C-NMR (150 MHz, DMSO-d_6) δ 96.9 (t, J = 27.5 Hz), 100.8 (d, J = 30.2 Hz), 119.1, 124.7, 126.0, 131.6, 134.5, 136.8, 142.3 (t, J = 14.4 Hz), 151.8, 153.8, 162.6 (d, J = 229.1 Hz). ^{19}F-NMR (376 MHz, DMSO-d_6) δ -116.1 (2F, t, J = 9.0 Hz). IR (KBr, cm^{-1}) 3378, 3273, 3102, 1671, 1611, 1577, 1478, 1230. LRMS (FAB) m/z 477.0 ([M+H+4]$^+$), 475.0 ([M+H+2]$^+$), 473.0 ([M+H]$^+$). HRMS (FAB) Calcd for $C_{14}H_9Br_2F_2N_6O^+$ ([M+H]$^+$), 472.9173, Found 472.9162.

***N*-(3-Trifluoromethylphenyl)-*N'*-[2,4-di(bromo)-6-(2*H*-tetrazol-5yl)phenyl]urea UA33**

Colorless solid. M.p. 206.8-208.0 °C (decomposed). ^{1}H-NMR (400 MHz, DMSO-d_6) δ 7.29–7.26 (m, 1H), 7.49–7.44 (m, 2H), 7.83 (s, 1H), 8.06 (brs, 1H), 8.16 (brs, 1H), 8.62 (s, 1H), 9.51 (s, 1H). ^{13}C-NMR (150 MHz, DMSO-d_6) δ 114.0 (brq, J = 4.4 Hz), 118.2 (brq, J = 4.4 Hz), 119.0, 121.6, 124.7, 124.1 (q, J = 271.7 Hz), 125.9, 129.4 (q, J = 30.2 Hz), 129.9, 131.5, 134.7, 136.8, 140.4, 152.0, 153.8. ^{19}F-NMR (376 MHz, DMSO-d_6) δ -62.8 (s, 3F). IR (KBr, cm^{-1}) 3339, 3271, 3067, 2730, 1883, 1656, 1604, 1566, 1450, 1397, 1335, 1278, 1230, 1178, 1120, 1069, 793, 697. HRMS (ESI) Calcd for $C_{15}H_8Br_2F_3N_6O$ [M–H]$^-$ 502.9084, Found 502.9056.

***N*-[3,5-Dimethoxylphenyl]-*N'*-[2,4-dibromo-6-(2*H*-tetrazol-5yl)phenyl]urea UA41**

Colorless solid. M.p. 185.0 °C (decomposed). ^{1}H-NMR (400 MHz, DMSO-d_6) δ 9.13 (1H, s), 8.41 (1H, s), 8.16 (1H, s), 8.02 (5H, s), 7.82 (4H, s), 6.53 (2H, s), 6.11(1H, s), 3.67 (6H, s). ^{13}C-NMR (400 MHz, DMSO-d_6)δ 160.6, 151.7, 143.4, 141.1, 136.3, 134.9, 131.5, 129.9, 124.2, 118.5, 110.4, 108.2, 105.9, 96.4, 55.0. IR (KBr, cm^{-1}) 3474, 3329, 3006, 2838, 1614, 1469, 1153, 1071, 870, 759 cm^{-1}. HRMS (ESI) Calcd for $C_{16}H_{15}Br_2N_6O_3$ $[M+H]^+$: 496.9567. Found: 496.9681.

***N*-Phenyl-*N'*-[2,4-dibromo-6-(2*H*-tetrazol-5yl)phenyl]urea UA28**

Colorless solid. M.p. 212.0-213.0 °C (decomposed, Hexane/Ethyl acetate = 1/4). ^{1}H-NMR (400 MHz, DMSO-d_6) δ 6.93 (1H, t, J = 7.2 Hz), 7.22 (2H, t, J = 8.4 Hz), 7.29 (2H, d, J = 8.4 Hz), 8.02 (1H, d, J = 2.4 Hz), 8.18 (1H, d, J = 2.0 Hz), 8.46 (1H, bs), 9.15 (1H, bs); ^{13}C-NMR (100 MHz, DMSO-d_6) δ 118.2, 118.7, 122.2, 124.2, 125.7, 129.0, 131.8, 135.2, 137.0, 139.7, 152.0, 154.0. IR (KBr, cm^{-1}) 3291, 1654, 1602, 1496, 753, 692. LRMS (FAB) m/z 441.0 ($[M+H+4]^+$), 439.0 ($[M+H+2]^+$), 437.0 ($[M+H]^+$). HRMS (FAB) Calcd for $C_{14}H_{11}Br_2N_6O^+$ $[M+H]^+$ 436.9361, Found 436.9355.

***N*-[3,5-Dimethylphenyl]-*N'*-[2,4-dibromo-6-(2*H*-tetrazol-5yl)phenyl]urea UA25**

Colorless solid. M.p. 217-218 °C (decomposed, Reprecipitation from acetone-hexane). ^{1}H-NMR (400 MHz, DMSO-d_6) δ 2.17 (6H, s), 6.57 (1H, s), 6.91 (1H, s), 8.02 (1H, d, J = 2.4 Hz), 8.16 (1H, d, J = 2.0 Hz), 8.45 (1H, s), 8.99 (1H, s). ^{13}C-NMR (100 MHz, DMSO-d_6) δ 21.3, 116.0, 118.6, 123.8, 124.3, 125.7, 131.7, 135.3, 136.9, 137.9, 139.5, 152.0, 154.0. IR (KBr, cm^{-1}) 3273, 1656, 1573, 1225. LRMS (FAB, NBA) m/z 469.0 ([M+H+4]+), 467.0 ([M+H+2]+), 465.0 ([M+H]+). HRMS (FAB, NBA): Calcd for $C_{16}H_{15}Br_2N_6O^+$ ([M+H]+) 464.9669, Found 464.9670.

***N*-[3,5-Bis(trifluoromethyl)phenyl]-*N'*-[2,4-dichloro-6-(2*H*-tetrazol-5yl)phenyl]urea No.44**

Colorless solid. M.p. > 220 °C (decomposed.). ^{1}H-NMR (400 MHz, DMSO-d_6) δ 7.61 (1H, s), 7.94 (1H, d, J = 2.8 Hz), 7.98 (1H, d, J = 2.4 Hz), 8.03 (2H, s), 8.78 (1H, s), 9.85 (1H, s). ^{13}C-NMR (100 MHz, DMSO-d_6) δ 114.7, 114.8 (d, J = 3.7 Hz), 118.0, 123.5 (q, J = 271.9 Hz), 126.1, 128.4, 130.9 (q, J = 32.8 Hz), 131.5 (d, J = 3.7 Hz), 133.0, 134.4, 142.0, 152.6, 154.2. ^{19}F-NMR (376 MHz, DMSO-d_6) δ -63.2 (s, 6F). IR (KBr, cm^{-1}) 3315, 3085, 1663, 1581, 1385, 1280, 1129. MS (EI) m/z 484 (M^+, 57%), 255 (M^+-77, 100%). HRMS (EI) Calcd for $C_{16}H_8Cl_2F_6N_6O$ (M^+) 484.0041, Found 484.0036.

***N*-[3,5-Bis(trifluoromethyl)phenyl]-*N'*-[2-(2*H*-tetrazol-5yl)phenyl]urea No.43**

Colorless solid. Mp > 260 °C (Acetone/Hexane = 5/1). ^{1}H-NMR (400 MHz, DMSO-d_6) δ 7.26 (1H, td, J = 7.2, 1.2 Hz), 7.55 (1H, td, J = 7.6, 1.6 Hz), 7.65 (1H, s), 7.91 (1H, dd, J = 7.6, 1.2 Hz), 8.20 (2H, s), 8.32 (1H, d, J = 7.6 Hz), 10.1, 10.5. ^{13}C-NMR (100 MHz, DMSO-d_6) δ 112.2, 114.8 (d, J = 3.7 Hz), 114.9, 118.3 (d, J = 3.0 Hz), 121.8, 123.1, 123.5 (q, J = 271.9 Hz), 128.8, 130.9 (q, J = 32.8 Hz), 137.7, 142.1, 152.4, 154.3. ^{19}F-NMR (376 MHz, DMSO-d_6) δ -63.2. IR (KBr, cm^{-1}) 3288, 2920, 1619, 1576, 1280, 1131. MS (EI) m/z 416 (M^+, 1%), 255 (M^+-161, 100%). HRMS Calcd. for $C_{16}H_{10}N_6F_6O$: 416.0820. Found: 416.0835.

***N*-[3,5-Bis(trifluoromethyl)phenyl]-*N'*-(2,4-dibromophenyl)urea No.45**

Colorless solid. Mp 204.0-205.0 °C (Acetone/Hexane = 5/1). ^{1}H-NMR (400 MHz, DMSO-d_6) δ 7.55 (1H, dd, J = 8.8, 2.4 Hz), 7.67 (1H, s), 7.88 (1H, d, J = 2.4 Hz), 7.99 (1H, d, J = 9.2 Hz), 8.08 (2H, s), 8.38 (1H, bs), 10.14 (1H, bs). ^{13}C-NMR (100 MHz, DMSO-d_6) δ 115.0, 115.6, 118.11, 118.14, 123.5 (q, J = 271.9 Hz), 124.3, 131.0 (q, J = 32.0 Hz), 131.3, 134.5, 136.3, 141.6, 152.2. ^{19}F-NMR (376 MHz, DMSO-d_6) δ -63.1. IR (KBr, cm^{-1}) 3331, 3106, 1666, 1578, 1389, 1279. MS (EI) m/z 504 (M^+, 1%), 71 (M^+-433, 100%). HRMS Calcd. for $C_{15}H_8Br_2F_6N_2O$: 503.8908. Found: 503.8935.

***N*-[3,5-bis(trifluoromethyl)phenyl]-*N'*-(2,4-dibromo-6-chlorophenyl)urea UA21**

Colorless solid. M.p. >260 °C (decomp. Toluene). ^{1}H-NMR (400 MHz, acetone-d_6) δ 7.62 (1H, s), 7.79 (1H, d, J = 2.0 Hz), 7.89 (1H, d, J = 2.0 Hz), 8.05 (1H, bs), 8.21 (2H, s), 9.14 (1H, bs). ^{13}C-NMR (100 MHz, acetone-d_6) δ 115.8 (hep, J = 3.7 Hz), 119.0 (d, J = 3.8 Hz), 121.3, 124.4 (q, J = 270.5 Hz), 126.6, 132.5, 132.5 (q, J = 32.7 Hz), 134.8, 134.8, 136.4, 142.7, 152.8. ^{19}F-NMR (376 MHz, acetone-d_6) δ -63.0. IR (KBr, cm^{-1}) 3301, 1650, 1276, 682. LRMS (FAB) *m/z*: 542.9 ([**M**+H+4]$^+$), 540.9 ([**M**+H+2]$^+$), 538.9 ([**M**+H]$^+$); HRMS (FAB): Calcd for $C_{15}H_8Br_2ClF_6N_2O^+$ ([**M**+H]$^+$): 538.8596, Found: 538.8582.

***N*-[3,5-bis(trifluoromethyl)phenyl]-*N'*-(2,4-dibromo-6-cyanophenyl)urea UA22**

Colorless solid. Mp. 202.0-203.0 °C (decomp, AcOEt). ^{1}H-NMR (400 MHz, acetone-d_6) δ 7.65 (1H, s), 8.08 (1H, d, J = 2.4 Hz), 8.21 (2H, s), 8.22 (1H, d, J = 2.4 Hz), 8.37 (1H, bs), 9.31 (1H, bs). ^{13}C-NMR (100 MHz, acetone-d_6) δ 115.5, 116.3 (hep, J = 3.8 Hz), 116.4, 119.3 (d, J = 3.8 Hz), 120.4, 124.3 (q, J = 274.2 Hz), 124.4, 132.5 (q, J = 32.8 Hz), 135.8, 139.2, 140.4, 142.3, 152.8, 162.9. ^{19}F-NMR (376 MHz, acetone-d_6) δ -63.0. IR (KBr, cm^{-1}) 3302, 2238, 1663, 1278. LRMS (FAB) *m/z*: 533.9 ([**M**+H+4]$^+$), 531.9 ([**M**+H+2]$^+$), 529.9 ([**M**+H]$^+$); HRMS (FAB): Calcd for $C_{16}H_8Br_2F_6N_3O^+$ ([**M**+H]$^+$): 529.8938, Found: 529.8910.

***N*-[3,5-bis(trifluoromethyl)phenyl]-*N'*-[3,5-dibromo-(1,1'-biphenyl)-2-yl]urea UA34**

Colorless solid. Mp. > 210 °C (sublimation, Toluene). ^{1}H-NMR (400 MHz, acetone-d_6) δ 7.33-7.41 (3H, m), 7.45 (2H, dd, J = 1.2, 8.0 Hz), 7.55 (2H, d, J = 2.0 Hz), 7.76 (1H, bs), 7.89 (1H, dd, J = 0.8, 2.0 Hz), 8.05 (2H, s), 8.83 (1H, bs). ^{13}C-NMR (100 MHz, acetone-d_6) δ 115.5 (t, J = 3.7 Hz), 118.9, 121.4, 124.4 (q, J = 270.4), 126.9, 128.9, 129.2, 129.5, 132.3 (q, J = 32.7 Hz), 133.4, 134.3, 134.9, 138.8, 142.8, 145.5, 153.5. ^{19}F-NMR (376 MHz, acetone-d_6) δ –62.9. IR (KBr, cm^{-1}) 3296, 1639, 1280, 701, 683. MS (EI) *m/z* 581 (M^++1, 50%). HRMS Calcd for $C_{21}H_{13}Br_2F_6N_2O$: 580.9299. Found: 580.9283.

***N*-[3,5-(ditrifluoromethyl)phenyl]-*N'*-[2,4-diiodo-6-(2*H*-tetrazol-5yl)phenyl]urea UA48**

Pale pink solid. Mp. 190°C (decomp, Toluene).^{1}H-NMR (400 MHz, CD_3OD) δ 8.48 (1H, d), 8.26 (1H, d), 7.96 (1H, s), 7.53 (1H, s). ^{13}C-NMR (400 MHz, CD_3OD) δ 156.4, 154.6, 150.5 (q, J = 8.6 Hz), 142.7, 139.6, 138.8, 133.2 (q, J = 32.6 Hz), 127.1, 124.7 (q, J = 270.5 Hz), 119.5, 116.3, 103.4, 92.8. ^{19}F-NMR (376 MHz, CD_3OD) δ –64.6. IR (KBr, cm^{-1}) 3333, 3089, 1669, 1567, 1385, 1280, 1181, 1128, 892, 700 cm^{-1}. HRMS (ESI) Calcd for $C_{16}H_9F_6I_2N_6O$ [M+H]$^+$: 668.8826. Found: 668.8886.

^{1}H-NMR: **UA49**

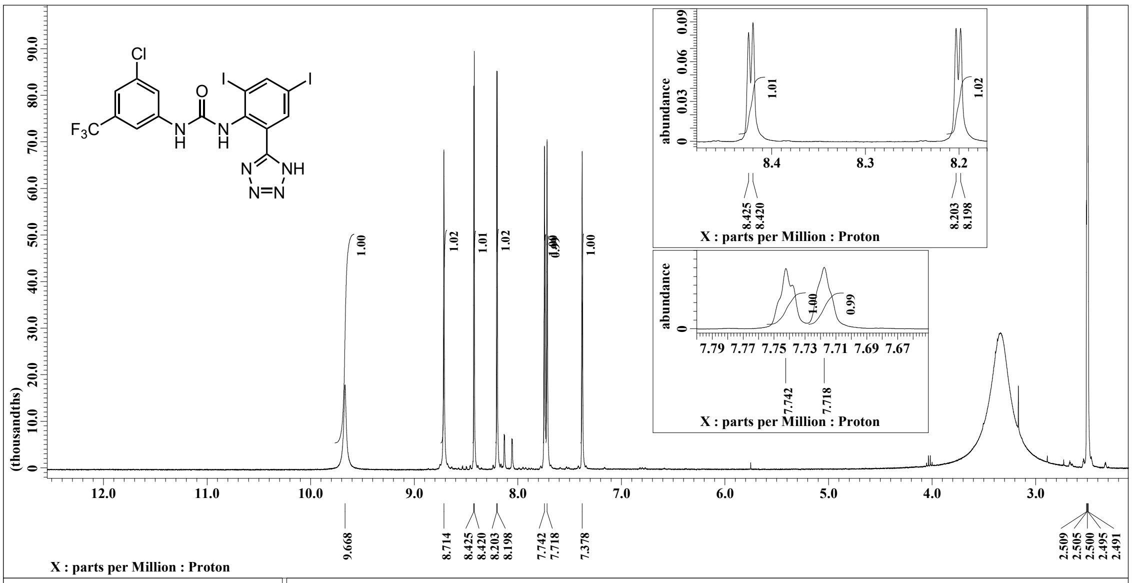


```
Filename          = 20240316UA49_proton-1-2.jdf
Author            = delta
Experiment        = proton.jxp
Sample_Id         = 20240316UA49
Solvent           = DMSO-D6
Actual_Start_Time = 17-MAR-2024 00:21:47
Revision_Time     = 15-NOV-2024 11:48:50

Comment           = single_pulse
Data_Format       = 1D COMPLEX
Dim_Size          = 52429
X_Domain          = Proton
Dim_Title         = Proton
Dim_Units         = [ppm]
Dimensions        = X
Site              = JNM-ECZL400S
```

^{13}C-NMR: **UA49**

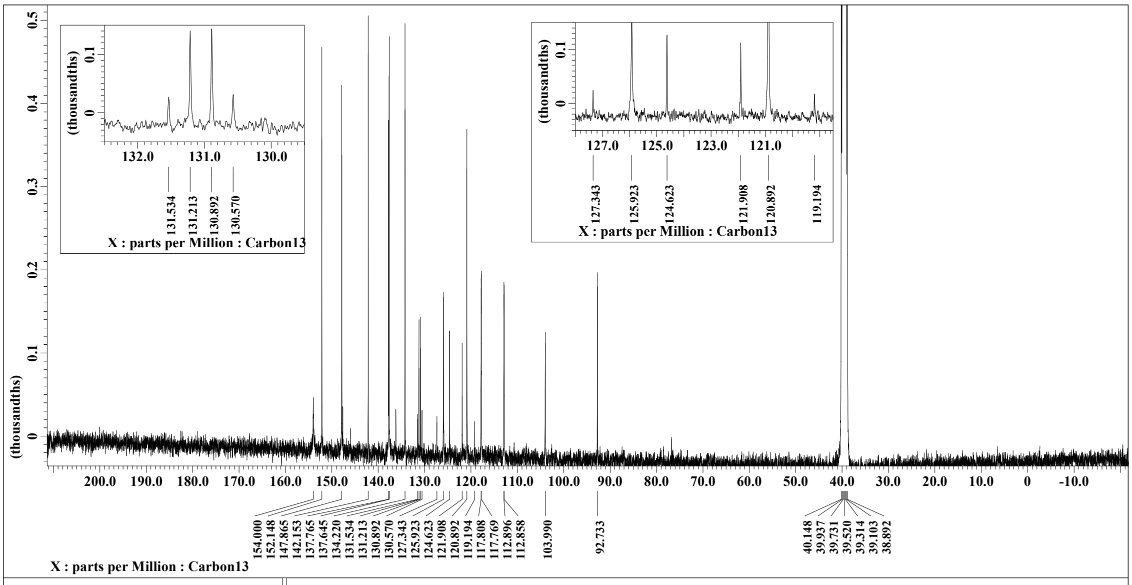


```
Filename          = 20240316UA49_carbon-1-2.jdf
Author            = delta
Experiment        = carbon.jxp
Sample_Id         = 20240316UA49
Solvent           = DMSO-D6
Actual_Start_Time = 17-MAR-2024 00:24:12
Revision_Time     = 18-MAR-2024 08:29:15

Comment           = single pulse decoupled gated NOE
Data_Format       = 1D COMPLEX
Dim_Size          = 52429
X_Domain          = Carbon13
Dim_Title         = Carbon13
Dim_Units         = [ppm]
Dimensions        = X
Site              = JNM-ECZL400S
```

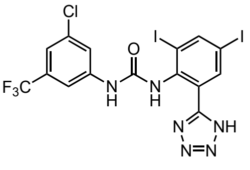

^{19}F-NMR: **UA49**

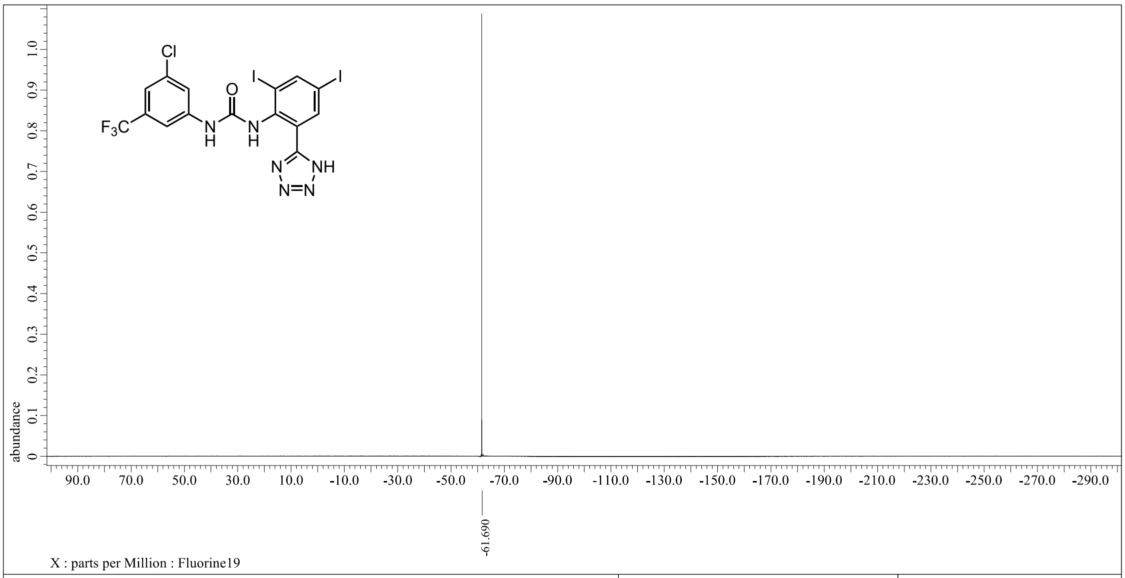

Filename	= 20240130UA49_proto	Spectrometer	= NM-70010S4L1
Author	= delta		
Experiment	= proton.jxp	Field_Strength	= 9.3803762[T] (400[
Sample_Id	= 20240130UA49	X_Acq_Duration	= 0.4325376[s]
Solvent	= DMSO-D6	X_Domain	= Fluorine19
Actual_Start_Time	= 30-JAN-2024 09:06:	X_Freq	= 375.79488151[MHz]
Revision_Time	= 17-MAR-2024 22:13:	X_Offset	= -100[ppm]
		X_Points	= 81920
Comment	= single_pulse	X_Prescans	= 1
Data_Format	= 1D COMPLEX	X_Resolution	= 2.31193774[Hz]
Dim_Size	= 209715	X_Sweep	= 189.39393939[kHz]
X_Domain	= Fluorine19	X_Sweep_Clipped	= 151.51515152[kHz]
Dim_Title	= Fluorine19	Irr_Domain	= Fluorine19
Dim_Units	= [ppm]	Irr_Freq	= 375.79488151[MHz]
Dimensions	= X	Irr_Offset	= 5[ppm]
Site	= JNM-ECZL400S	Tri_Domain	= Fluorine19

---- PROCESSING PARAMETERS ----
sexp(1.15597[Hz], 0.0[s])
trapezoid(0[%], 0[%], 80[%], 100[%])
zerofill(2, TRUE)
fft(1, TRUE, TRUE)
machinephase
ppm

以下に由来:: 20240130UA49_proton-2-1.jdf

^{1}H-NMR: **NS5806 (UA14)**

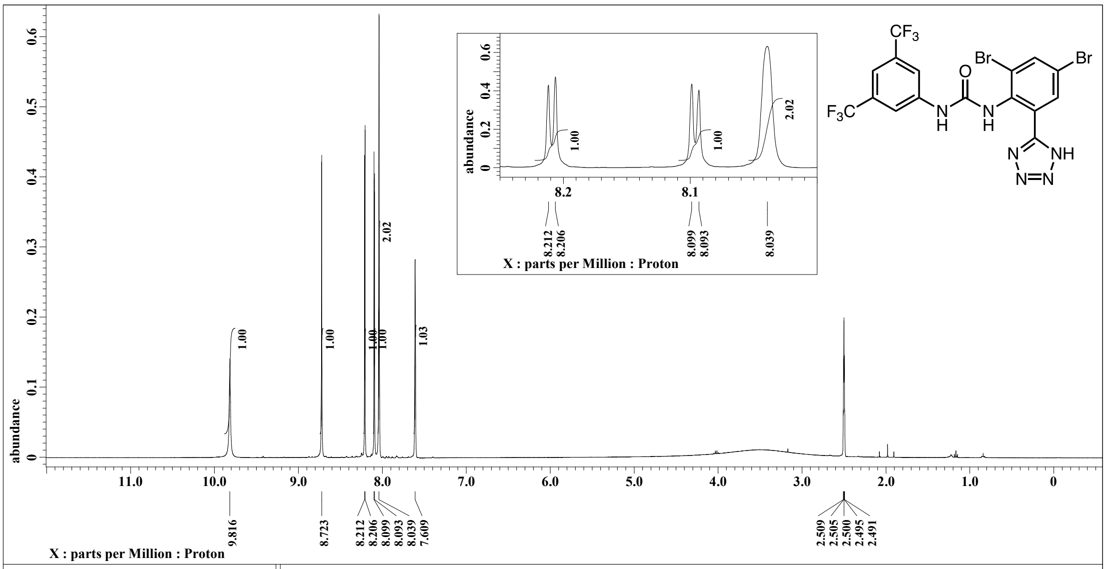

Filename	= 20241114UA-14_proton-1-3.jdf
Author	= delta
Experiment	= proton.jxp
Sample_Id	= 20241114UA-14
Solvent	= DMSO-D6
Actual_Start_Time	= 14-NOV-2024 22:29:14
Revision_Time	= 15-NOV-2024 11:33:07
Comment	= single_pulse
Data_Format	= 1D COMPLEX
Dim_Size	= 52429
X_Domain	= Proton
Dim_Title	= Proton
Dim_Units	= [ppm]
Dimensions	= X
Site	= JNM-ECZL400S

^{13}C-NMR: **NS5806 (UA14)**

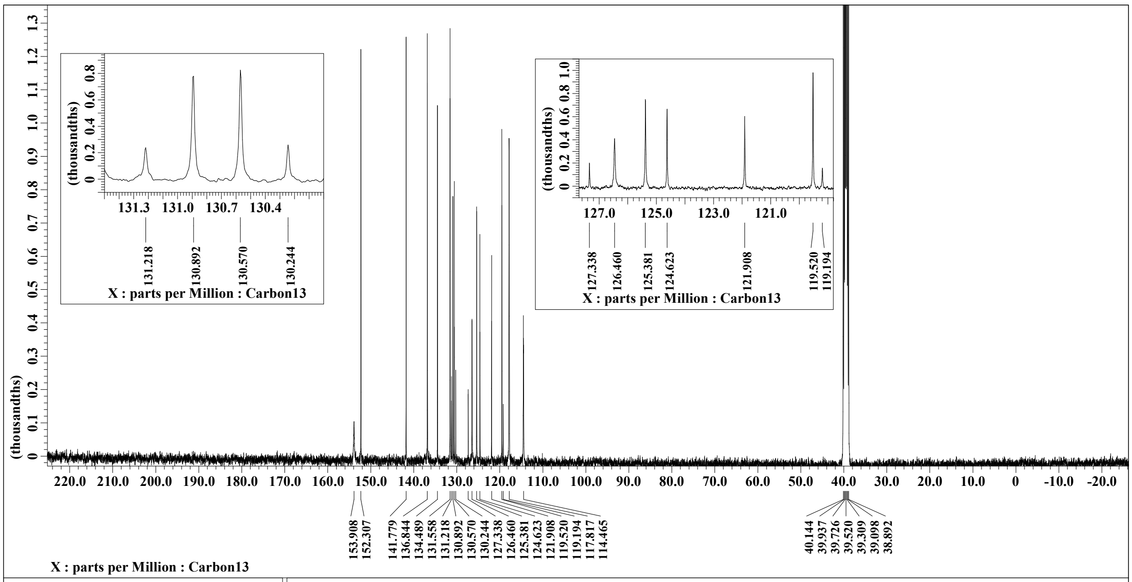

Filename	= 20241114UA-14-13C_carbon-1-1.jdf
Author	= delta
Experiment	= carbon.jxp
Sample_Id	= 20241114UA-14
Solvent	= DMSO-D6
Actual_Start_Time	= 14-NOV-2024 22:46:41
Revision_Time	= 15-NOV-2024 08:25:10
Comment	= single pulse decoupled gated NOE
Data_Format	= 1D COMPLEX
Dim_Size	= 52429
X_Domain	= Carbon13
Dim_Title	= Carbon13
Dim_Units	= [ppm]
Dimensions	= X
Site	= JNM-ECZL400S

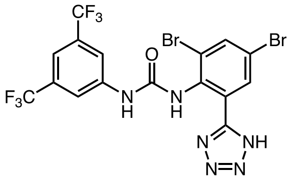

^{19}F-NMR: **NS5806 (UA14)**

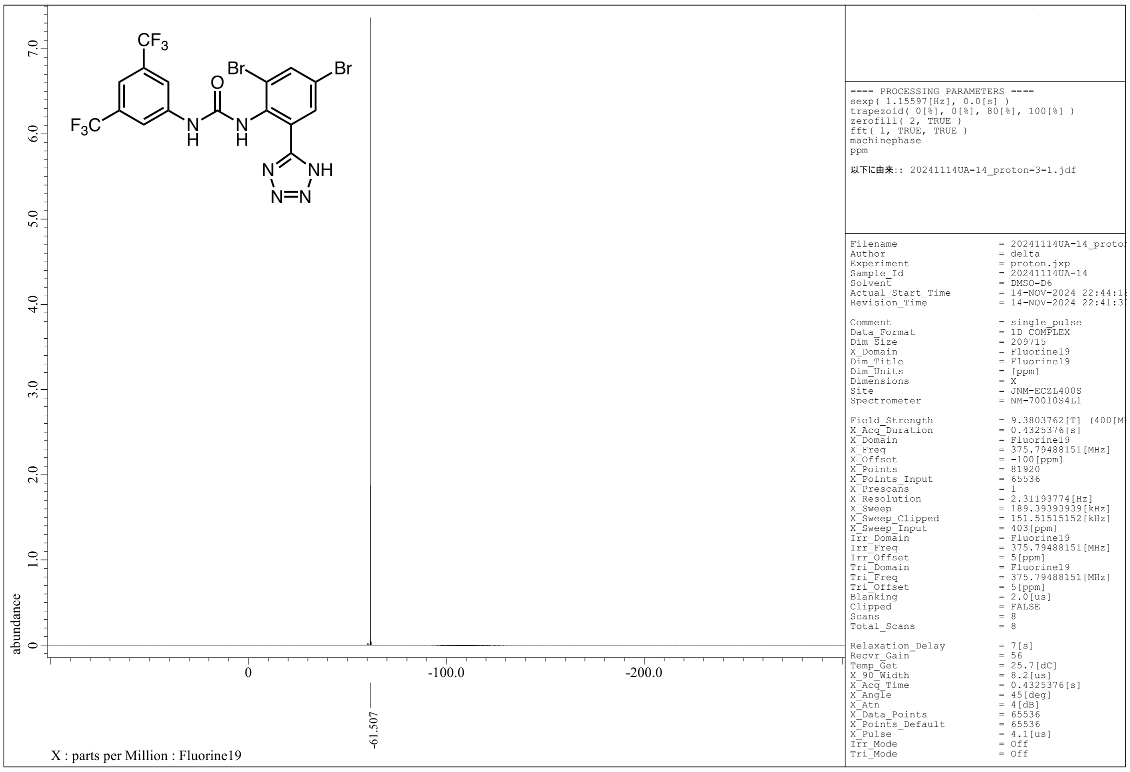


```
---- PROCESSING PARAMETERS ----
sexp( 1.15597[Hz], 0.0[s] )
trapezoid( 0[%], 0[%], 80[%], 100[%] )
zerofill( 2, TRUE )
fft( 1, TRUE, TRUE )
machinephase
ppm

以下に由来:: 20241114UA-14_proton-3-1.jdf
```

```
Filename                = 20241114UA-14_proto
Author                  = delta
Experiment              = proton.jxp
Sample_Id               = 20241114UA-14
Solvent                 = DMSO-D6
Actual_Start_Time       = 14-NOV-2024 22:44:1
Revision_Time           = 14-NOV-2024 22:41:3

Comment                 = single_pulse
Data_Format             = 1D COMPLEX
Dim_Size                = 209715
X_Domain                = Fluorine19
Dim_Title               = Fluorine19
Dim_Units               = [ppm]
Dimensions              = X
Site                    = JNM-ECZL400S
Spectrometer            = NM-70010S4L1

Field_Strength          = 9.3803762[T] (400[M
X_Acq_Duration          = 0.4325376[s]
X_Domain                = Fluorine19
X_Freq                  = 375.79488151[MHz]
X_Offset                = -100[ppm]
X_Points                = 81920
X_Points_Input          = 65536
X_Prescans              = 1
X_Resolution            = 2.31193774[Hz]
X_Sweep                 = 189.39393939[kHz]
X_Sweep_Clipped         = 151.51515152[kHz]
X_Sweep_Input           = 403[ppm]
Irr_Domain              = Fluorine19
Irr_Freq                = 375.79488151[MHz]
Irr_Offset              = 5[ppm]
Tri_Domain              = Fluorine19
Tri_Freq                = 375.79488151[MHz]
Tri_Offset              = 5[ppm]
Blanking                = 2.0[us]
Clipped                 = FALSE
Scans                   = 8
Total_Scans             = 8

Relaxation_Delay        = 7[s]
Recvr_Gain              = 56
Temp_Get                = 25.7[dC]
X_90_Width              = 8.2[us]
X_Acq_Time              = 0.4325376[s]
X_Angle                 = 45[deg]
X_Atn                   = 4[dB]
X_Data_Points           = 65536
X_Points_Default        = 65536
X_Pulse                 = 4.1[us]
Irr_Mode                = Off
Tri_Mode                = Off
```

Supplemental Table

Arabidopsis strains used in this study.

Resource	Source	Identifier
lc-LysM GEPII1.0, t-DNA line in Columbia 0 (Col-0)	Wang et al., 2021	N/A
NES-YC3.6, t-DNA line in Col-0	Krebs et al, 2011	N/A
NES-YC3.6, t-DNA line in Wassilewskija (Ws)	This study	N/A
NES-YC3.6, t-DNA line in *kincless*	This study	N/A
YC3.6, t-DNA line in *osca1-1*	Yuan et al., 2014	N/A
YC3.6, t-DNA line in *osca1.3/1.7*	Thor et al, 2020	N/A
NES-YC3.6, t-DNA line in *hpca1-1*	Wu et al., 2020	N/A
ER-GCaMP6-210, t-DNA line in Col-0	Resentini et al., 2021	N/A
GFP-fABD2, t-DNA line in Col-0	Voigt et al., 2005	N/A
112458, t-DNA line in Col-0	Gonzalez-Guzman et al., 2012	N/A
ost1, t-DNA line in Col-0	ABRC	SALK_008068
cpk3-2 cpk6-1, t-DNA line in Col-0	Mori et al., 2006	N/A
slac1, t-DNA line in Col-0	Brandt et al., 2015	N/A
SLAC1/slac1-1, t-DNA line in Col-0	Brandt et al., 2015	N/A
SLAC1 S59A S120A/slac1-1, t-DNA line in Col-0	Brandt et al., 2015	N/A
cas-1, t-DNA line in Col-0	ABRC	SALK_070416
eds5-1, t-DNA line in Col-0	Glazebrook et al., 1996	N/A
kincless, t-DNA line in Ws	Lebaudy et al., 2008	N/A
gork, t-DNA line in Col-0	ABRC	GABI_865F05
