# Supplementary material for: Synthetic ion channel inhibitors enhance plant drought tolerance
Source: Nat Commun. 2026 Jul 27;17:7257. doi: 10.1038/s41467-026-75894-w (PMC13408460; doi:10.1038/s41467-026-75894-w)
Supplement: Supplementary file 2 — Description of Additional Supplementary Files [file 41467_2026_75894_MOESM2_ESM.pdf]

Description of Additional Supplementary Files

File Name: Supplementary Movie 1

Description: Cytosolic Ca^{2+} imaging in guard cells of *Arabidopsis thaliana* (Mock control).

Leaf epidermal strips were treated with 0.2% DMSO (mock control) at 0 s. False-color images show the cpVenus/CFP ratio in guard cells expressing NES-YC3.6 at the indicated time. To compose the panel, images were cropped. Low cpVenus/CFP ratios, indicating low Ca^{2+} are shown in black, and high cpVenus/CFP ratios indicating high Ca^{2+} are shown in white. Scale bar = 20 µm.

File Name: Supplementary Movie 2

Description: Cytosolic Ca^{2+} imaging in guard cells of *Arabidopsis thaliana* (NS5806).

Leaf epidermal strips were treated with 10 µM NS5806 at 0 s. False-color images show the cpVenus/CFP ratio in guard cells expressing NES-YC3.6 at the indicated time. To compose the panel, images were cropped. Low cpVenus/CFP ratios, indicating low Ca^{2+} are shown in black, and high cpVenus/CFP ratios indicating high Ca^{2+} are shown in white. Scale bar = 20 µm.

File Name: Supplementary Movie 3

Description: Cytosolic Ca^{2+} imaging in guard cells of *Arabidopsis thaliana* (UA49).

Leaf epidermal strips were treated with 10 µM UA49 at 0 s. False-color images show the cpVenus/CFP ratio in guard cells expressing NES-YC3.6 at the indicated time. To compose the panel, images were cropped. Low cpVenus/CFP ratios, indicating low Ca^{2+} are shown in black, and high cpVenus/CFP ratios indicating high Ca^{2+} are shown in white. Scale bar = 20 µm.
