# Supplementary material for: Synthetic ion channel inhibitors enhance plant drought tolerance
Source: Nat Commun. 2026 Jul 27;17:7257. doi: 10.1038/s41467-026-75894-w (PMC13408460; doi:10.1038/s41467-026-75894-w)
Supplement: Supplementary file 6 — Reporting Summary [file 41467_2026_75894_MOESM6_ESM.pdf]

nature portfolio

Corresponding author(s): Nobuyuki Uozumi

Last updated by author(s): Jun 1, 2026

Reporting Summary

Nature Portfolio wishes to improve the reproducibility of the work that we publish. This form provides structure for consistency and transparency in reporting. For further information on Nature Portfolio policies, see our Editorial Policies and the Editorial Policy Checklist.

Statistics

For all statistical analyses, confirm that the following items are present in the figure legend, table legend, main text, or Methods section.

n/a	Confirmed	
☐	☒	The exact sample size (*n*) for each experimental group/condition, given as a discrete number and unit of measurement
☐	☒	A statement on whether measurements were taken from distinct samples or whether the same sample was measured repeatedly
☐	☒	The statistical test(s) used AND whether they are one- or two-sided<br>*Only common tests should be described solely by name; describe more complex techniques in the Methods section.*
☒	☐	A description of all covariates tested
☒	☐	A description of any assumptions or corrections, such as tests of normality and adjustment for multiple comparisons
☐	☒	A full description of the statistical parameters including central tendency (e.g. means) or other basic estimates (e.g. regression coefficient) AND variation (e.g. standard deviation) or associated estimates of uncertainty (e.g. confidence intervals)
☐	☒	For null hypothesis testing, the test statistic (e.g. *F*, *t*, *r*) with confidence intervals, effect sizes, degrees of freedom and *P* value noted<br>*Give P values as exact values whenever suitable.*
☒	☐	For Bayesian analysis, information on the choice of priors and Markov chain Monte Carlo settings
☒	☐	For hierarchical and complex designs, identification of the appropriate level for tests and full reporting of outcomes
☒	☐	Estimates of effect sizes (e.g. Cohen's *d*, Pearson's *r*), indicating how they were calculated

Our web collection on statistics for biologists contains articles on many of the points above.

Software and code

Policy information about availability of computer code

Data collection	Microsoft Excel (version 2604) and Graph Pad Prism (version 10.6.0) were used for data collection. Commercial pCLAMP (version 11.4.2) software was used to obtain the electrophysiological recordings.
Data analysis	Microsoft Excel (version 2604), CoStat (version 6.45), and Graph Pad Prism (version 10.6.0) were used for data collection.

For manuscripts utilizing custom algorithms or software that are central to the research but not yet described in published literature, software must be made available to editors and reviewers. We strongly encourage code deposition in a community repository (e.g. GitHub). See the Nature Portfolio guidelines for submitting code & software for further information.

Data

Policy information about availability of data

All manuscripts must include a data availability statement. This statement should provide the following information, where applicable:

- Accession codes, unique identifiers, or web links for publicly available datasets
- A description of any restrictions on data availability
- For clinical datasets or third party data, please ensure that the statement adheres to our policy

The authors declare that all data supporting the findings of this study are available within this article, Source Data file, and Extended Data files. RNA-seq data have been deposited in the DDBJ Sequence Read Archive at the DNA Data Bank (http://www.ddbj.nig.ac.jp/) with the BioProject accession number PRJDB20282.

Research involving human participants, their data, or biological material

Policy information about studies with human participants or human data. See also policy information about sex, gender (identity/presentation), and sexual orientation and race, ethnicity and racism.

Reporting on sex and gender	Not applicable
Reporting on race, ethnicity, or other socially relevant groupings	Not applicable
Population characteristics	Not applicable
Recruitment	Not applicable
Ethics oversight	Not applicable

Note that full information on the approval of the study protocol must also be provided in the manuscript.

Field-specific reporting

Please select the one below that is the best fit for your research. If you are not sure, read the appropriate sections before making your selection.

[x] Life sciences   [ ] Behavioural & social sciences   [ ] Ecological, evolutionary & environmental sciences

For a reference copy of the document with all sections, see nature.com/documents/nr-reporting-summary-flat.pdf

Life sciences study design

All studies must disclose on these points even when the disclosure is negative.

Sample size	No statistical methods were used to predetermine sample size. Sample size were chosen to produce biologically meaningful data for comparison purposes, based on previous experiments of out laboratory and other using the physiological and biochemical methods described.
Data exclusions	All experimental data were subjected to outlier analysis using GraphPad Prism (ROUT method, Q=1%).
Replication	All experiments were conducted with at least two independent biological replicates and gave similar results.
Randomization	Randomization was not relevant to this study because all experiments were performed using genetically identical plants grown under identical, strictly controlled environmental conditions, ensuring that sample characteristics were homogeneous prior to treatment allocation.
Blinding	Blinding was not performed. Blinding was not possible because the same investigator processed the samples and analyzed the data.

Reporting for specific materials, systems and methods

We require information from authors about some types of materials, experimental systems and methods used in many studies. Here, indicate whether each material, system or method listed is relevant to your study. If you are not sure if a list item applies to your research, read the appropriate section before selecting a response.

Materials & experimental systems

n/a	Involved in the study	
[ ]	[x]	Antibodies
[x]	[ ]	Eukaryotic cell lines
[x]	[ ]	Palaeontology and archaeology
[x]	[ ]	Animals and other organisms
[x]	[ ]	Clinical data
[x]	[ ]	Dual use research of concern
[ ]	[x]	Plants

Methods

n/a	Involved in the study	
[x]	[ ]	ChIP-seq
[x]	[ ]	Flow cytometry
[x]	[ ]	MRI-based neuroimaging

Antibodies

Antibodies used	Rabbit anti-H+-ATPase polyclonal atibody (1:3000 dilution) and rabbit anti-pen-pThr polyclonal (1:3000 dilution) have been produced by our research groups.
Validation	The antibody profiles and validations are available in the following references.<br><br><Rabbit anti-H+-ATPase polyclonal antibody>

Polyclonal antibody against the conserved catalytic domain of the plasma membrane H+-ATPase of Arabidopsis were raised in rabbit. (Hayashi Y, Nakamura S, Takemiya A, Takahashi Y, Shimazaki K and Kinoshita T. Plant Cell Physiol. 51:1186-1196, 2010)

<Rabbit anti-pen-pThr polyclonal antibody>
Polyclonal antibody against the penultimate phospharylated Thr947 of the plasma membrane H+-ATPase of Arabidosis were raised in rabbits using the phosphorylated synthetic peptide (CIETPSHYpTV) as an antigen. (Hayashi Y, Nakamura S, Takemiya A, Takahashi Y, Shimazaki K and Kinoshita T. Plant Cell Physiol. 51:1186-1196, 2010)

Plants

Seed stocks

Columbia-0 (Col-0) and Wassilewskija (Ws) were used as the wild-type. Col-0 is a background ecotype of lc-LysM GEPII1.0(Wang et al., 2021), NES-YC3.6 (Krebs et al, 2011), osca1-1 (Yuan et al., 2014), osca1.3/1.7 (Thor et al, 2020), hpca1-1 (Wu et al., 2020), ER-GCaMP6-210 (Resentini et al., 2021), GFP-fABD2 (Voigt et al., 2005), 112458 (Gonzalez-Guzman et al., 2012), ost1 (SALK_008068), cpk3-2 cpk6-1 (Mori et al., 2006), slac1 (Brandt et al., 2015), SLAC1/slac1-1 (Brandt et al., 2015), SLAC1 S59A S120A/slac1-1 (Brandt et al., 2015), cas-1 (SALK_070416), and eds5-1 (Glazebrook et al., 1996), gork (GABI_865F05). Ws is a background ecotype of YC3.6 and kincless (Lebaudy et al., 2008). The original WS and kincless seeds were kindly provided by Hervè Sentenac (Institute for Plant Sciences of Montpellier).

Novel plant genotypes

Wassilewskija (WS) WT and kincless mutant plants were transformed with the NES-YC3.6 Ca^{2+} indicator via floral dipping, using the original pTKan-NES-YC3.6 construct reported by Krebs et al. (2012)57. In this construct, the coding sequence of the sensor is controlled by the UBQ10 promoter and rbcs terminator.

Authentication

Transformants were selected using a fluorescence stereomicroscope equipped with a GFP filter, isolating fluorescent seedlings.
