# Supplementary material for: Synthetic ion channel inhibitors enhance plant drought tolerance
Source: Nat Commun. 2026 Jul 27;17:7257. doi: 10.1038/s41467-026-75894-w (PMC13408460; doi:10.1038/s41467-026-75894-w)
Supplement: Supplementary file 7 — Transparent Peer Review File [file 41467_2026_75894_MOESM7_ESM.pdf]

nature portfolio

Peer Review File

Synthetic ion channel inhibitors enhance plant drought tolerance

Corresponding Author: Professor Nobuyuki Uozumi

This file contains all reviewer reports in order by version, followed by all author rebuttals in order by version.

Version 0:

Reviewer comments:

Reviewer #2

(Remarks to the Author)
This paper reports a unique mechanism underlying one of most important plants' responses to the detrimental stress drought, stomata closure, preventing water loss and increasing survival. It is well known that the stress hormone ABA plays a central role in plants' responses to drought stress by triggering the closure of stomata pores to save water. It is also well known that voltage-dependent potassium (K+) channels functions in stomata opening. However, whether the manipulation of K+ channel activities by inhibitors could inhibit stomatal opening and thus promote relatively stomata closure has not been systematically analyzed and reported, although the hypothesis is interesting. These authors screened a potential K+ channel-inhibitor chemical library and identified two inhibitors, NS5806 and UA49. Subsequently, they carried out a enormous amount of experiments to analyze these two inhibitors' effects on K+ channel activities, stomatal movements, gene expression, as well as plants' responses to drought stress. Notably, they found that to achieve the beneficial effects under drought stress, these K+ channel inhibitors function differently from ABA, suggesting a previously unknown approach that could be used to manipulate stomatal movements under physiological conditions, which has a potential application value for agriculture. Note that, as ABA is a hormone that has a wide range of functions in plant growth and development. Manipulation of ABA signaling pathways could have dramatic side effects, which could cancel its beneficial effects on plants' responses to drought. Furthermore, they characterized the functioning mechanism of these two K+ channel inhibitors. They found these inhibitors trigger cytosolic Ca2+ increases via inhibiting K+ channels, which is a novel finding and may require further examination.

Overall, these findings are exciting and fundamentally important. The amount of experimental results are enormous, and the data are logically presented. This work makes a compelling case for the manipulation of K+ channel to regulate stomatal movements under drought stress, and provides a significant addition to our understating of drought stress signalling networks.

Major concerns:
--- The finding of NS5806/UA49-induced Ca2+ increases is interesting and important. The NS5806/UA49-induced Ca2+ increases occurred relatively slowly, reached the peak around 4 min, and lasted over 30 min, differing from Ca2+ increases induced by other stimuli, such as ABA and flg22 as stated in the paper. These authors stated that "Both the influx of Ca^{2+} from the extracellular space and the possible release of Ca2+ from the ER are contributing factors to this process (Fig. 5c-f).". If this is the case, the effects of these two inhibitors are pleotropic, rather than specific to K+ channels. Note that, many chemicals/stimuli could trigger Ca2+ increases. ---"NS5806/UA49 did not directly inhibit the ICa channel in the patch clamp measurements (Fig. 1e). The defective K+ inward channel mutants kincless39 exhibited a marked decrease in cytosolic Ca2+ elevation caused by NS5806/UA49 (Fig. 4g, h). These results indicate that hyperpolarization caused by the NS5806/UA49-mediated inhibition of inward K+ channels, such as KAT1, might activate plasma membrane Ca^{2+} influx channels." There are a few factors that regulate Ca2+ signaling in stomatal guard cells, such as mechanical/osmotic stress and H2O2. Whether H2O2 or osmotic stress is involved in this process should be examined and discussed, considering NS5806/UA49 could affect the turgor pressure of guard cells.

--- Given that Slac1, OST1 and CPK3/6 are involved in stomatal movements, using corresponding mutants these authors

found that NS5806/UA49 has limited involvement in the ABA pathway. Do NS5806/UA49 affect the osmotic and H2O2 signaling pathway? This could be examined using the corresponding mutants of osca1 and hpca1 mutants.

Minor comments:
-- Line 112: "We conducted a chemical screen of an in-house chemical library for inhibitors of KAT1, a dominant voltage-dependent inward K+ channel in Arabidopsis guard cells23, 24", ref 23, 24 have nothing to do with K+ channels.

-- NS5806 inhibited KAT1 activity by 55%, and UA49 inhibited KAT1 activity by 79% using oocytes. However, the dose-effect was not analysed. Similarly, the dose-effect on Kin currents in guard cells should be analysed.

-- The leaf temperature data points were not shown in Fig. 2b, and the statistical analysis was not shown too.

Reviewer #3

(Remarks to the Author)
Review on the manuscript
'Synthetic ion channel inhibitors enhance plant drought tolerance' by Kanane Sato et al.

The current manuscript reports on the identification and development of two inhibitors of plasma membrane voltage-dependent potassium channels, NS5806 and UA49, that induce stomatal closure, reduce guard cell K+ levels, and increase drought resistance in Arabidopsis.

The paper is well written and technically sound. Experiments have been conducted using state-of-the-art techniques which are well documented. This work represents an interesting and novel approach with considerable interest for the plant science community. In addition, the results presented suggest possible agricultural application potential. The data provided however are rather preliminary and open questions remain as outlined below.

1. It's remarkable that KAT2 a close homolog of KAT1 is hardly affected by the inhibitors. What are the structural constraints? What is the general mechanism of action of these inhibitors? Is it an open pore block – like with TEA+ or Cs+ or does it affect voltage dependent gating? This could be tested easily in Xenopus oocyte experiments.
2. In addition, the compound are quite hydrophobic and to me it's not clear whether they act from the extracellular or cytoplasmic side. What about reversibility and dose dependency?
3. Line 123 and fig. 1c: again, compared to KAT1, KAT2 is not inhibited at all by NS5806! -Why?
4. Apparently, NS5806/UA49 can both promote stomatal closure and inhibit stomatal opening. Is fusicoccin mediated stomatal opening inhibited, too?
5. "NS5806/UA49 treatment resulted in a decrease in the cytosolic K+ concentration in guard cells". Is this decrease sustained or transient, does it compare to kincless!!
6. I am not convinced that the inhibitors act guard cell specific. What about mesophyll or leave/whole plant K+ content in the long term? Since K+ homeostasis is crucial for plant development, how do these inhibitors affect plant development, flowering, seed set and productivity?
7. Gene expression analysis is not very informative at this stage. How do guard cell expression profiles compare to those of leaves (mesophyll)? In fig. 3f clustering should be performed to obtain a better picture about differentially expressed transcripts. Gene lists should be provided as suppl. data.
8. Inhibitor induced stomatal closing is different from ABA. GO analysis shows that differentially addressed genes comprise wounding and JA related pathways. Is inhibitor mediated stomatal closing impaired in JA signaling mutants?
9. Inhibitor induced stomatal closure is impaired in the slac1 mutant, a convergence point for many pathways addressing stomatal movements. Stomatal closure requires the concerted release of anions and potassium. The latter is mediated by K-efflux channels like GORK, which is also addressed by the inhibitors (see fig 1). What is the role of K-efflux channels in this process?
10. Related to the question above. Is it possible that these inhibitor act as activators of anion channels?
11. It appears puzzling that inhibitors do not affect guard cell Ca2+ channels but induce a sustained Ca2+ signal in guard cells. The causal relationship between Ca2+ signaling and inhibitor induced stomatal closure has not been provided, but needs…
12. Do other K+ channels inhibitors like TEA or Cs act in a comparable manner in mediating a Ca2+ response and induction of stomatal closure? If not, what distinguishes the new inh
13. In addition, inhibitor induced Ca2+ signaling is impaired in the kincless mutant lacking functional K+ channels due to the heteromerization potential of the underlying KAT1 mutant. Kout channels however do not assemble into heteromers with Kin channels, pointing to a possible important role of Kout channels. This issue has not been clarified (see above).
14. What is the effect of the inhibitors on guard cell/mesophyll cell membrane potential? Do they really result in hyperpolarization as suggested? Please test.
15. Do these inhibitors really act in a guard cell specific manner and locally or do they act also in a systemic manner? Would the inhibitors affect K+ channels from other plant species or would even act cross species wide (e.g. insect K+ channels)
16. In the Ca2+ imaging movies guard cells appear open and do not close, please comment.
17. Fig. 4g, h is figure 5
18. …effect on stomatal closure implies previously unrevealed cross-talk between K+ channels, anion channels, and Ca2+ channels. Actually, this find is not new. The potassium/calcium interrelationship is well known and has been used by the authors to impose potassium dependent calcium changes to plant cells.

Version 1:

Reviewer comments:

Reviewer #3

(Remarks to the Author)
Review on the revised manuscript by Sato et al. entitled 'Synthetic ion channel inhibitors enhance plant drought tolerance'

First, I would like to express my satisfaction with the manner and extent to which the authors have answered the open questions. The revised manuscript in its present form has improved significantly.
The following issues have been addressed and were answered extensively based on new experimental data:
1. Inhibitor site of action
Assuming the inhibitors could act on the cytosolic site of Kin channels, domain swapping approaches between KAT1/KAT2 identified the C-terminal region of KAT1 to be involved in the inhibitory mechanism of NS5806. Unfortunately, the reciprocal chimera did not show increased sensitivity to NS5806. Likewise, UA49 exhibited consistent inhibition across all chimeric variants.
On the other hand, the authors could convincingly demonstrate the reversibility of inhibitor action in wash-out experiments, which would suggest that these compounds primarily influence channel activity from the extracellular side of the plasma membrane. These data fit well the observed inhibition of animal Shaker channels that exhibit structurally different C-termini compared to plant Kin channels.
This important finding should be included in the manuscript (discussion)
2. Inhibitor mode of action
Re-evaluation of the electrophysiological data strongly suggest that the inhibitors act as gating modifiers. This is an important observation adding to the understanding of stomatal physiological responses is not found in the revision. Since the authors have also addressed the dose dependency it would be helpful getting the IC50 values.
3. Specificity
In new experiments the authors now show that the inhibitors inhibit guard cells specifically and this information is now found in the manuscript.
4. Physiological context
a) In new experiments the authors observed that following a 2-hour pre-treatment with FC, the stomatal closure normally induced by NS5806 was completely abolished. Thus, fusicoccin could overcome inhibitor mediated inhibition of blue light dependent H+-ATPase phosphorylation (Fig. 3). On the other hand, and based on DiBAC data, UA49 seems to induce membrane hyperpolarization, which would require pronounced H+ ATPase activity. Still, this finding adds to the understanding of mode of inhibitor action and would fit to the observed shift in voltage dependent gating triggered by the inhibitors – an information not found in the revised version.
b) In another set of new experiments, the authors show that when using kincless and gork mutants, stomatal closure typically induced by NS5806/UA49 was completely abolished. This strongly supports the idea that suggests that both K+ inward channels KAT1/KAT2 and the outward K+ channel GORK are indispensable for the full physiological response – an information that is not found in the revised version.
c) Finally, new experiments demonstrate that NS5806-induced stomatal closure was significantly impaired in coi1-1, indicating that this inhibitor requires a functional JA signaling pathway to exert its full effect. However, UA49-induced stomatal closure seems JA independent and remained unaffected in the coi1-1 mutant. This is an important physiological context and thus the inhibitors may help to dissect the hormone signaling pathway in guard cells. Please discuss.
In summary, the authors have addressed the open questions and points of criticism with great care, considerable effort and a significant number of new experiments. Like always, several questions remain. For example it is not sufficient to block Kin channels (see Cs+ and TEA data) to induce Ca+ signals. The pharmacological data employing animal Shaker K channels also may point towards a neurotoxic function of these inhibitors, requiring caution in the context of using the inhibitors in their current states for agricultural purposes.

Open Access This Peer Review File is licensed under a Creative Commons Attribution 4.0 International License, which permits use, sharing, adaptation, distribution and reproduction in any medium or format, as long as you give appropriate credit to the original author(s) and the source, provide a link to the Creative Commons license, and indicate if changes were made.
In cases where reviewers are anonymous, credit should be given to 'Anonymous Referee' and the source.
The images or other third party material in this Peer Review File are included in the article's Creative Commons license, unless indicated otherwise in a credit line to the material. If material is not included in the article's Creative Commons license and your intended use is not permitted by statutory regulation or exceeds the permitted use, you will need to obtain permission directly from the copyright holder.
To view a copy of this license, visit https://creativecommons.org/licenses/by/4.0/

Response to Reviewers:

Thank you very much for assessing our manuscript entitled "Synthetic ion channel inhibitors enhance plant drought tolerance" (NCOMMS-25-17471-T). Based on the helpful editor's and reviewers' comments, we are now submitting the revised version. We have extensively performed the additional experiments and carefully addressed the reviewers' comments and concerns. The specific changes made are detailed in our enclosed "Point-by-point response to the reviewers' comments" where we have reproduced their feedback verbatim alongside our responses.

REVIEWER COMMENTS

Reviewer #2 (Remarks to the Author):

This paper reports a unique mechanism underlying one of most important plants' responses to the detrimental stress drought, stomata closure, preventing water loss and increasing survival. It is well known that the stress hormone ABA plays a central role in plants' responses to drought stress by triggering the closure of stomata pores to save water. It is also well known that voltage-dependent potassium (K^+) channels functions in stomata opening. However, whether the manipulation of K^+ channel activities by inhibitors could inhibit stomatal opening and thus promote relatively stomata closure has not been systematically analyzed and reported, although the hypothesis is interesting. These authors screened a potential K^+ channel-inhibitor chemical library and identified two inhibitors, NS5806 and UA49. Subsequently, they carried out a enormous amount of experiments to analyze these two inhibitors' effects on K^+ channel activities, stomatal movements, gene expression, as well as plants' responses to drought stress. Notably, they found that to achieve the beneficial effects under drought stress, these K^+ channel inhibitors function differently from ABA, suggesting a previously unknown approach that could be used to manipulate stomatal movements under physiological conditions, which has a potential application value for agriculture. Note that, as ABA is a hormone that has a wide range of functions in plant growth and development. Manipulation of ABA signaling pathways could have dramatic side effects, which could cancel its beneficial effects on plants' responses to drought. Furthermore, they characterized the functioning mechanism of these two K^+ channel inhibitors. They found these inhibitors trigger cytosolic Ca^{2+} increases via inhibiting K^+ channels, which is a novel finding and may require further examination.

Overall, these findings are exciting and fundamentally important. The amount of experimental results are enormous, and the data are logically presented. This work makes a compelling case for the manipulation of K^+ channel to regulate stomatal movements under drought stress, and provides a significant addition to our understating of drought stress signalling networks.

Response: Thank you very much for your extensive review. We are grateful to the reviewer for positive comments and suggestions.

Major concerns:

--- The finding of NS5806/UA49-induced Ca^{2+} increases is interesting and important. The NS5806/UA49-induced Ca^{2+} increases occurred relatively slowly, reached the peak around 4 min, and lasted over 30 min, differing from Ca^{2+} increases induced by other stimuli, such as ABA and flg22 as stated in the paper. These authors stated that "Both the influx of Ca^{2+} from the extracellular space and the possible release of Ca^{2+} from the ER are contributing factors to this process (Fig. 5c-f).". If this is the case, the effects of these two inhibitors are pleotropic, rather than specific to K^+ channels. Note that, many chemicals/stimuli could trigger Ca^{2+} increases.

Response: We sincerely thank the reviewer for their critical insight regarding the origin of the observed cytosolic Ca^{2+} increase. To address this, we re-evaluated our interpretation using a more direct and sensitive approach. Specifically, we employed Arabidopsis lines expressing the ER-localized Ca^{2+} sensor, ER-GCaMP6-210, to monitor Ca^{2+} dynamics within the endoplasmic reticulum (ER) lumen. Our imaging in guard cells revealed that treatment with NS5806 and UA49 increased ER Ca^{2+} levels (New Data 1). Given that the ER membrane contains both CPA-sensitive and CPA-insensitive Ca^{2+}-ATPases (Grenzi *et al.*, *New Phytol.* (2025)), our previous data was limited to the effects on the CPA-sensitive fraction. Accordingly, we have replaced the original Fig. 5e and Fig. 5f with the ER-GCaMP6-210 imaging results, updated the manuscript (Line#209–212 and 255–256), and modified the method (added Line #446-450, 465-467, and 471, and deleted Line#490). Extended Data Figure 7 has been removed, and the corresponding descriptions in the text have been revised as follows:

-Revised Manuscript Line #209-212

In the original manuscript, "Additionally, when guard cells were treated with cyclopiazonic acid (CPA), an inhibitor of ER Ca^{2+}-ATPases that depletes Ca^{2+} in the ER[11], the increase in cytosolic Ca^{2+} caused by NS5806/UA49 was decreased (Fig. 5e, f; Extended Data Fig. 7). These data indicate that NS5806/UA49 promoted Ca^{2+}influx from the plasma membrane and required proper homeostasis of ER Ca^{2+}."

In the revised manuscript, "Additionally, to directly monitor Ca^{2+} dynamics within the endoplasmic reticulum (ER), we used Arabidopsis lines expressing the ER-localized Ca^{2+} indicator, ER-GCaMP6-210[41]. Our analyses in guard cells revealed that treatment with NS5806/UA49 also induced an increase in Ca^{2+} levels in the ER (Fig. 5e–g), supporting the conclusion that the apoplast is the main source of Ca^{2+} sustaining the cytosolic Ca^{2+} increase."

-Revised Manuscript Line #255-256

In the original manuscript, "Both the influx of Ca^{2+} from the extracellular space and the possible release of Ca^{2+} from the ER are contributing factors to this process (Fig. 5c-f)."

In the revised manuscript, "The influx of Ca^{2+} from the extracellular space is a contributing factor to this process (Fig. 5c-g). Nevertheless, "

-Deleted original sentence in Method (Line #490)

"For the experiments aimed to study the contribution of ER Ca^{2+} homeostasis in the generation of NS5806/UA49-induced cytosolic Ca^{2+} increase, 3 h after stomatal opening induction, the epidermal strips were treated with 20 µM CPA (cyclopiazonic acid) for 20 min before the adding NS5806/UA49, following the 30 min imaging."

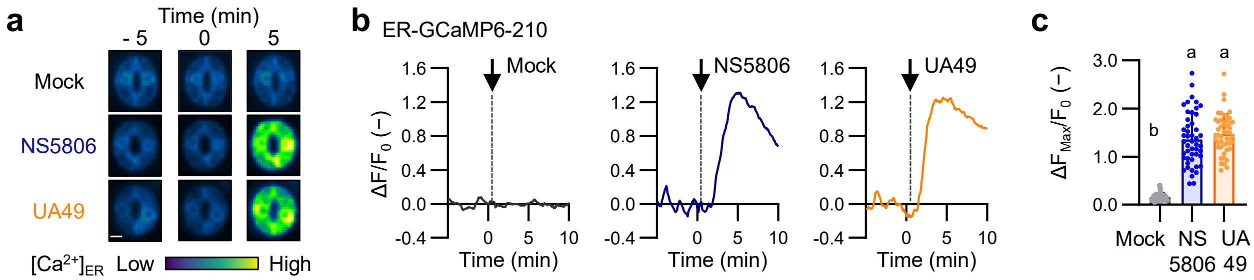

New Data 1 (Fig. 5e-g): NS5806 and UA49 promote Ca^{2+} uptake into the ER.

Ca^{2+} imaging was performed with Arabidopsis ER-GCaMP6-210 expressing line. Guard cells were treated with 10 µM NS5806/UA49 at 0 min as indicated in the graph. The first 300 seconds were removed to wait for the stabilization of the signal. **a**, False-color shows GCaMP6 fluorescence intensity at the indicated time in guard cells. Scale bar = 5 µm. **b**, The representative time course data of normalized GCaMP6 fluorescence intensity. **c**, The peak normalized fluorescence intensity as $\Delta F/F_0$ maximum (mean ± SD, n = 46-48). Bars marked with different letters are significantly different ($p < 0.05$) by one-way ANOVA with Tukey–Kramer test.

---"NS5806/UA49 did not directly inhibit the ICa channel in the patch clamp measurements (Fig. 1e). The defective K^+ inward channel mutants kincless[39] exhibited a marked decrease in cytosolic Ca^{2+} elevation caused by NS5806/UA49 (Fig. 4g, h). These results indicate that hyperpolarization caused by the NS5806/UA49-mediated inhibition of inward K^+ channels, such as KAT1, might activate plasma membrane Ca^{2+} influx channels." There are a few factors that regulate Ca^{2+} signaling in stomatal guard cells, such as mechanical/osmotic stress and H_2O_2. Whether H2O2 or osmotic stress is involved in this process should be examined and discussed, considering NS5806/UA49 could affect the turgor pressure of guard cells.

Response: We thank the reviewer for the constructive suggestion to examine whether NS5806/UA49-induced Ca^{2+} signaling shares common mechanisms with established stress-sensing pathways. To address this, we performed Ca^{2+} imaging using Arabidopsis lines expressing YC3.6 in various mutant backgrounds (New Data 2). Our results showed that while NS5806 and UA49 still induced cytosolic Ca^{2+} increases in *hpca1-1* (H_2O_2-insensitive, Wu *et al.*, *Nature* (2020)) and *osca1-1* (osmotic stress-insensitive, Yuan *et al.*, *Nature* (2014)) mutants, the peak Ca^{2+} levels induced by UA49 were notably lower than those observed in the wild-type (New Data 2a-d). Furthermore, in the *osca1.3/1.7* double mutant, which is impaired in pathogen-induced stomatal signaling (Thor *et al.*, *Nature* (2020)), both NS5806 and UA49 elicited reduced maximum Ca^{2+} levels (New Data 2e, f). NS5806 appears to partially require OSCA1.3/1.7 activity, while UA49 exhibits a broader overlap with HPCA1, OSCA1.1, and OSCA1.3/1.7. We have added New Data 2 as Extended Data Fig. 9, the text below in the Manuscript Line #213-221. According to the change, we have modified the introduction (Line#98-99).

Added sentences

"Such changes of cytosolic Ca^{2+} levels in guard cells are key signatures of responses to various stimuli, mediated by specific channels such as HYDROGEN-PEROXIDE-INDUCED Ca^{2+} INCREASES (HPCA1) for H_2O_2[21], HYPEROSMOLALITY-GATED Ca^{2+} PERMEABLE CHANNEL 1.1 (OSCA1.1) for osmotic stress[19], and OSCA1.3/1.7 for pathogen signaling[20]. To determine whether NS5806 and UA49 affect these pathways, we performed Ca^{2+} imaging, in the cytosol, in their respective mutant backgrounds (Extended Data Fig. 9). While NS5806 affected the peak Ca^{2+} increase only in *osca1.3/1.7*, UA49 induced significant differences in all three lines (*hpca1-1*, *osca1-1*, and *osca1.3/1.7*) relative to the WT. Collectively, these data suggest that NS5806 signaling is partially dependent on OSCA1.3/1.7, whereas UA49 exhibits a broader overlap with HPCA1, OSCA1.1, and OSCA1.3/1.7 pathways to execute stomatal response."

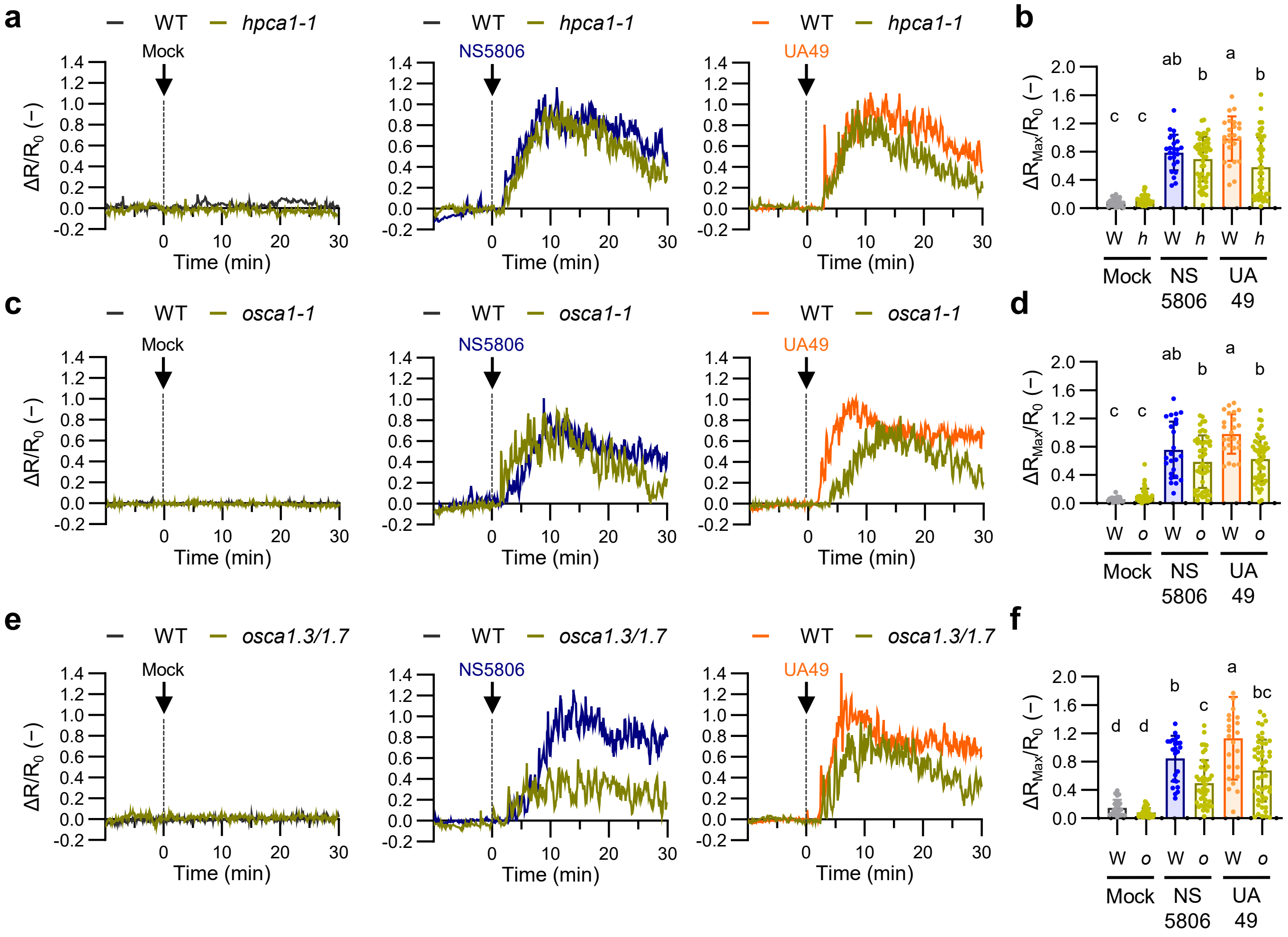

New Data 2 (Extended Data Fig. 9): Cytosolic Ca^{2+} imaging of Arabidopsis calcium channel-deficient mutants expressing YC3.6 upon treatment with NS5806 or UA49.

Data were collected in the same way as Fig 5a, b. Guard cells were treated with 10 µM NS5806/UA49 at 0 min as indicated in the graphs. **a** and **b**, *hpca1-1* (n = 24-48), **c** and **d**, *osca1-1* (n = 22-48), **e** and **f**, *osca1.3/1.7* (n = 24-48). **a**, **c**, and **e** show the representative time course. **b**, **d**, and **f** show the peak cpVenus/CFP ratios as $\Delta R/R_0$ maximum (mean ± SD). Bars marked with different letters are significantly different ($p < 0.05$) by one-way ANOVA with Tukey–Kramer test.

--- Given that Slac1, OST1 and CPK3/6 are involved in stomatal movements, using corresponding mutants these authors found that NS5806/UA49 has limited involvement in the ABA pathway. Do NS5806/UA49 affect the osmotic and H_2O_2 signaling pathway? This could be examined using the corresponding mutants of osca1 and hpca1 mutants.

Response: Thank you very much for these important comments. As described above, NS5806 does not require OSCA1.1 or HPCA1 activity for the cytosolic Ca^{2+} increase, whereas our data suggest that OSCA1.3/1.7 are at least partially required. In contrast, UA49 partially requires OSCA1.1 and OSCA1.3/1.7. Together, these results indicate that OSCA channels contribute to this response with a certain degree of specificity toward different chemical stimuli.

Minor comments:

-- Line 112: "We conducted a chemical screen of an in-house chemical library for inhibitors of KAT1, a dominant voltage-dependent inward K^+ channel in Arabidopsis guard cells[23], [24]", ref 23, 24 have nothing to do with K^+ channels.

Response: We thank the reviewer for the careful reading and for identifying this error. We agree with your suggestions. We have removed these citations from the revised manuscript (Line #116).

-- NS5806 inhibited KAT1 activity by 55%, and UA49 inhibited KAT1 activity by 79% using oocytes. However, the dose-effect was not analysed. Similarly, the dose-effect on Kin currents in guard cells should be analysed.

Response: We have performed additional electrophysiological characterization using the *Xenopus* oocyte heterologous expression system. Our results clearly demonstrate that both NS5806 and UA49 inhibit KAT1 currents in a dose-dependent manner (New Data 3). Regarding the patch-clamp analysis of guard cell protoplasts, we regret to inform the reviewer that we were unable to perform reliable experiments since the chemical concentrations higher than 10 μM prevented the formation of giga-ohm seals and reliable measurements.

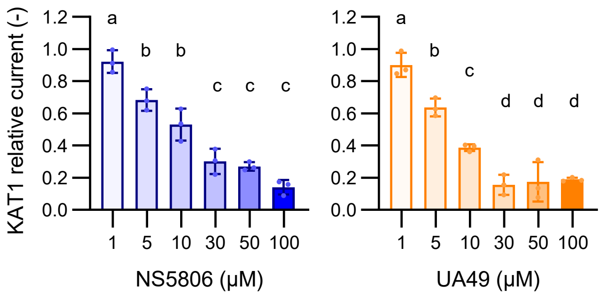

New Data 3: NS5806 and UA49 inhibit K^+ channels in a dose-dependent manner.

Dose dependency test of NS5806 and UA49 on KAT1. Two-electrode voltage clamp was performed using *Xenopus laevis* oocytes for each channel with or without NS5806 or UA49 in the bath solution, and relative currents were plotted (n = 3, mean ± SD). The current value was collected at the end of the pulse at −170 mV. Bars marked with different letters are significantly different ($p < 0.05$) by one-way ANOVA with Tukey–Kramer test.

-- The leaf temperature data points were not shown in Fig. 2b, and the statistical analysis was not shown too.

Response: Thank you for your insightful advice. We calculated the average values of the data shown in Fig. 2b and Extended Data Figure 2a in the original manuscript and performed statistical analysis (New Data 4). A significant temperature increase was observed in UA49-treated plants, but although the same tendency for temperature increase was observed with NS5806, there was no statistically significant difference. According to your suggestion, we have replaced the figure (Fig. 2b), deleted the original Extended Data Fig. 2a, and modified the related sentence in the manuscript as below (Line #140-144) and the figure legends (Fig. 2b).

In the original manuscript, "Additionally, thermal imaging revealed that, compared with untreated controls (Mock), NS5806/UA49, like ABA, increased the leaf temperature, suggesting that NS5806/UA49 induced stomatal closure (Fig. 2b; Extended Data Fig. 2a)."

In the revised manuscript, "Additionally, thermal imaging revealed that UA49 treatment induced an increase in leaf temperature, similar to ABA, compared with untreated controls (Mock). Although no statistically significant difference was observed between Mock and NS5806, a trend toward higher leaf temperature was also detected in NS5806-treated plants, suggesting that both UA49 and NS5806 may promote stomatal closure (Fig. 2b)."

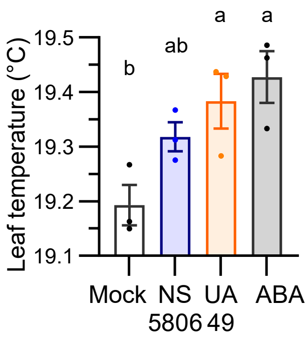

New Data 4: Recalculated leaf surface temperature data in Fig 2b and Extended Data Figure 2a.

Bars marked with different letters are significantly different ($p < 0.05$) by one-way ANOVA with Tukey–Kramer test (mean ± SEM, $n = 3$).

Reviewer #3 (Remarks to the Author):

Review on the manuscript
'Synthetic ion channel inhibitors enhance plant drought tolerance' by Kanane Sato et al.

The current manuscript reports on the identification and development of two inhibitors of plasma membrane voltage-dependent potassium channels, NS5806 and UA49, that induce stomatal closure, reduce guard cell K^+ levels, and increase drought resistance in Arabidopsis.

The paper is well written and technically sound. Experiments have been conducted using state-of-the-art techniques which are well documented. This work represents an interesting and novel approach with considerable interest for the plant science community. In addition, the results presented suggest possible agricultural application potential. The data provided however are rather preliminary and open questions remain as outlined below.

Response: We appreciate the reviewer's suggestions on how to improve our paper. Based on the feedback, we have revised the manuscript as follows.

1. It's remarkable that KAT2 a close homolog of KAT1 is hardly affected by the inhibitors. What are the structural constraints? What is the general mechanism of action of these inhibitors? Is it an open pore block - like with TEA^+ or Cs^+ or does it affect voltage dependent gating? This could be tested easily in Xenopus oocyte experiments.

Response: Thank you for your suggestions. The C-terminal region of KAT1 and KAT2 consists of a C-linker, followed by a Cyclic Nucleotide-Binding Homology Domain (CNBHD), and a distal C-terminal tail where homology between the two channels is particularly low (New Data 5a). To identify the structural domains responsible for the differential sensitivity to NS5806 and UA49, we generated two sets of chimeric constructs: (1) gene-swapped after the sixth transmembrane domain (S6) (New Data 5b) and (2) gene-swapped after the CNBHD (New Data 5c). In the S6-swapped chimeras, the inhibition rates of NS5806 and UA49 remained similar to those of their respective wild-type (WT) counterparts, suggesting that the N-terminal half (S1–S6) alone does not dictate the differences in sensitivity. Interestingly, in the chimeras swapped after the CNBHD, the inhibition of KAT1 by NS5806 was partially attenuated when its distal C-terminal region was replaced with that of KAT2. This indicates that the distal C-terminal region of KAT1 is involved in the inhibitory mechanism of NS5806. However, the reciprocal chimera—where the distal C-terminal region of KAT2 was replaced with that of KAT1—did not show increased sensitivity to NS5806, with inhibition rates remaining negligible, similar to WT KAT2. In contrast, UA49 exhibited consistent inhibition across all chimeric variants, comparable to WT levels. These findings suggest that the interaction sites for NS5806 and UA49, and their subsequent effects on channel activity, are not determined by a single linear domain in the C-terminus.

In addition, we have re-analyzed our electrophysiological data to further characterize the inhibitory mechanisms of NS5806 and UA49. The analysis revealed a difference between NS5806 and UA49 in the voltage-dependency of their actions. For NS5806, the inhibitory effect was partially relieved at stronger hyperpolarizing potentials, suggesting a voltage-dependent inhibition (New Data 6a, b). In contrast, the inhibitory ratio of UA49 remained constant across all tested pulse voltages, suggesting a voltage-independent mechanism. Furthermore, we analyzed the tail currents to assess the effects on channel gating. Although we were unable to calculate the precise half activation potential ($V_{1/2}$) because the saturating plateau of the sigmoid curve was not reached in the presence of the compounds, the clear shift and alteration in the I-V relationship of the tail currents suggest that both compounds may influence the gating process (New Data 6c). Taken together, these results imply that while both compounds act on the gating machinery, NS5806 may interact with the voltage-sensing domain or the pore in a voltage-sensitive manner, whereas UA49 acts via a more constitutive inhibitory mechanism. Unlike TEA^+ and Cs^+, NS5806/UA49 does not possess a significant positive charge, so it may not function as an apparent open-pore blocker. However, it cannot be ruled out that it still acts as an open-pore blocker. To fully elucidate the molecular basis of the distinct inhibitory actions of NS5806 and UA49, future structural studies, such as cryo-electron microscopy (cryo-EM) of the KAT1-inhibitor complex, will be indispensable. However, this is beyond the scope of this work.

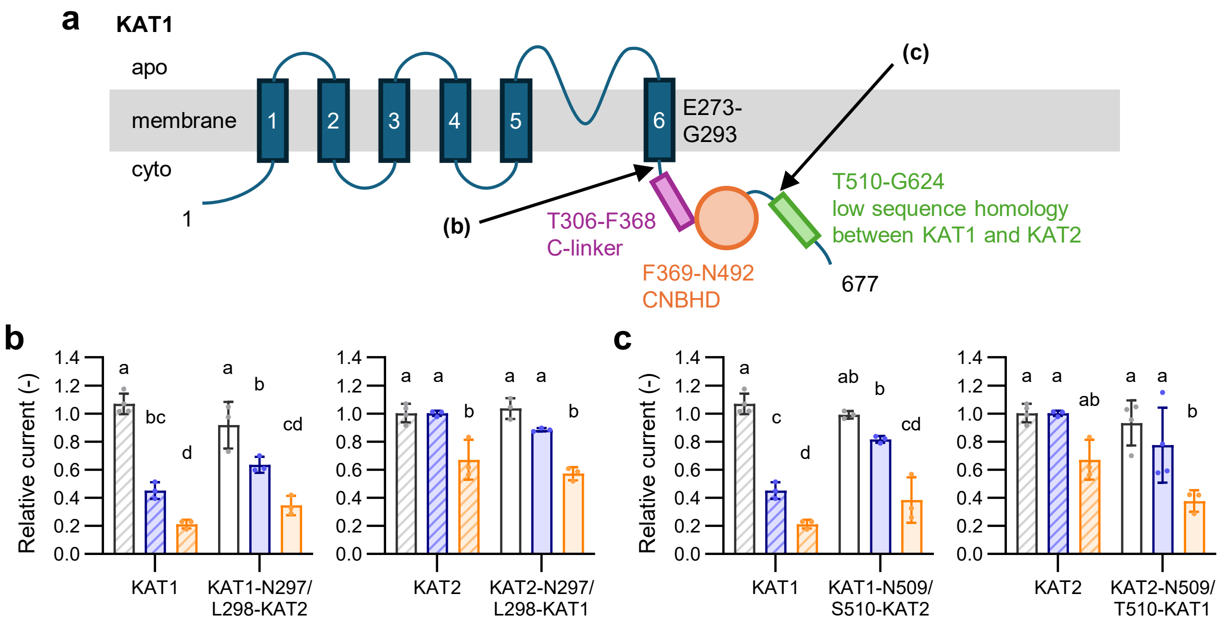

New Data 5: The effect of NS5806 and UA49 on gene-swapped between KAT1 and KAT2 channel.

a, Schematic image of KAT1. **b** and **c**, Two-electrode voltage clamp was performed using *Xenopus laevis* oocytes expressing chimeric channels in which segments were swapped between KAT1 and KAT2 either after the sixth transmembrane domain (**b**) or after the CNBHD (**c**). Oocytes were treated with 30 µM NS5806 or UA49 for 1 min. The current value was collected at the end of the pulse at −170 mV and relative currents were plotted (n = 3-4, mean ± SD). Bars marked with different letters are significantly different ($p < 0.05$) by one-way ANOVA with Tukey–Kramer test. Note: the wild-type KAT1 and KAT2 data are the same data as Fig 1c.

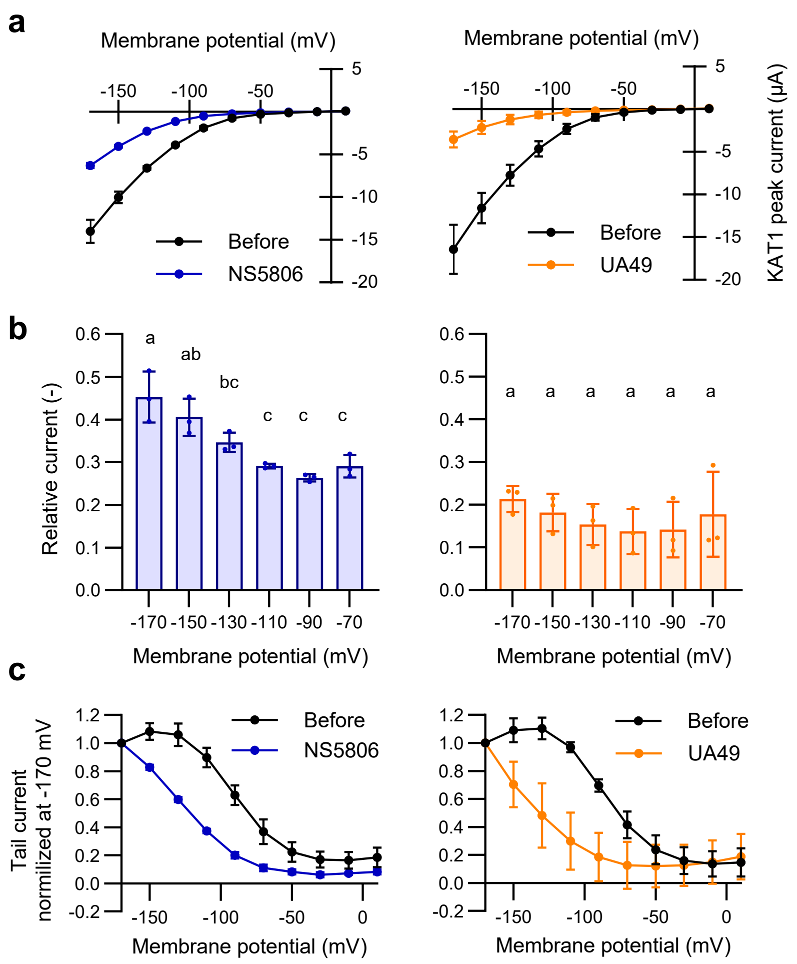

New Data 6: Reanalyzed data of KAT1 current obtained by two-electrode voltage clamp in Fig. 1c.

a, I-V diagram for peak current. The current value was collected at the end of the pulse. **b**, Relative peak currents at each pulse following treatment with 30 μM NS5806 or UA49. Bars marked with different letters are significantly different ($p < 0.05$) by one-way ANOVA with Tukey–Kramer test ($n = 3$, mean ± SD). **c**, I-V diagram for tail current. Current amplitudes were measured at 15 ms after stepping back to the holding voltage, and the values were plotted against the preceding pulse potentials.

2. In addition, the compound are quite hydrophobic and to me it's not clear whether they act from the extracellular or cytoplasmic side. What about reversibility and dose dependency?

Response: Thank you for pointing this out. To investigate whether NS5806 and UA49 target the extracellular or intracellular side of the plasma membrane, we performed two sets of experiments using the *Xenopus* oocyte system and Arabidopsis plants expressing the Ca^{2+} sensor NES-YC3.6. First, in the two-electrode voltage clamp experiments using KAT1-expressing oocytes, we measured the currents after a 1 min washout with a compound-free buffer following the initial treatment. We observed a significant, albeit partial, recovery of the KAT1 channel activity that had been inhibited by NS5806 or UA49 (New Data 7a, b). Second, we monitored Ca^{2+} dynamics in Arabidopsis guard cells. When the treatment solution was replaced with a pure buffer 5 min after the application of the compounds, the UA49-induced increase in cytosolic Ca^{2+} concentration was suppressed within 30 minutes (New Data 7c, d). Taken together, these results demonstrate that the inhibitory effects of NS5806 and UA49 are reversible to a certain extent. This suggests that these compounds primarily influence channel activity from the extracellular side of the plasma membrane rather than acting irreversibly or targeting the intracellular side. Furthermore, we evaluated the dose-dependency of NS5806 and UA49 using *Xenopus* oocytes expressing KAT1 (New Data 3a). Both compounds inhibited KAT1 currents in a concentration-dependent manner, further supporting their roles as potent inhibitors of this channel.

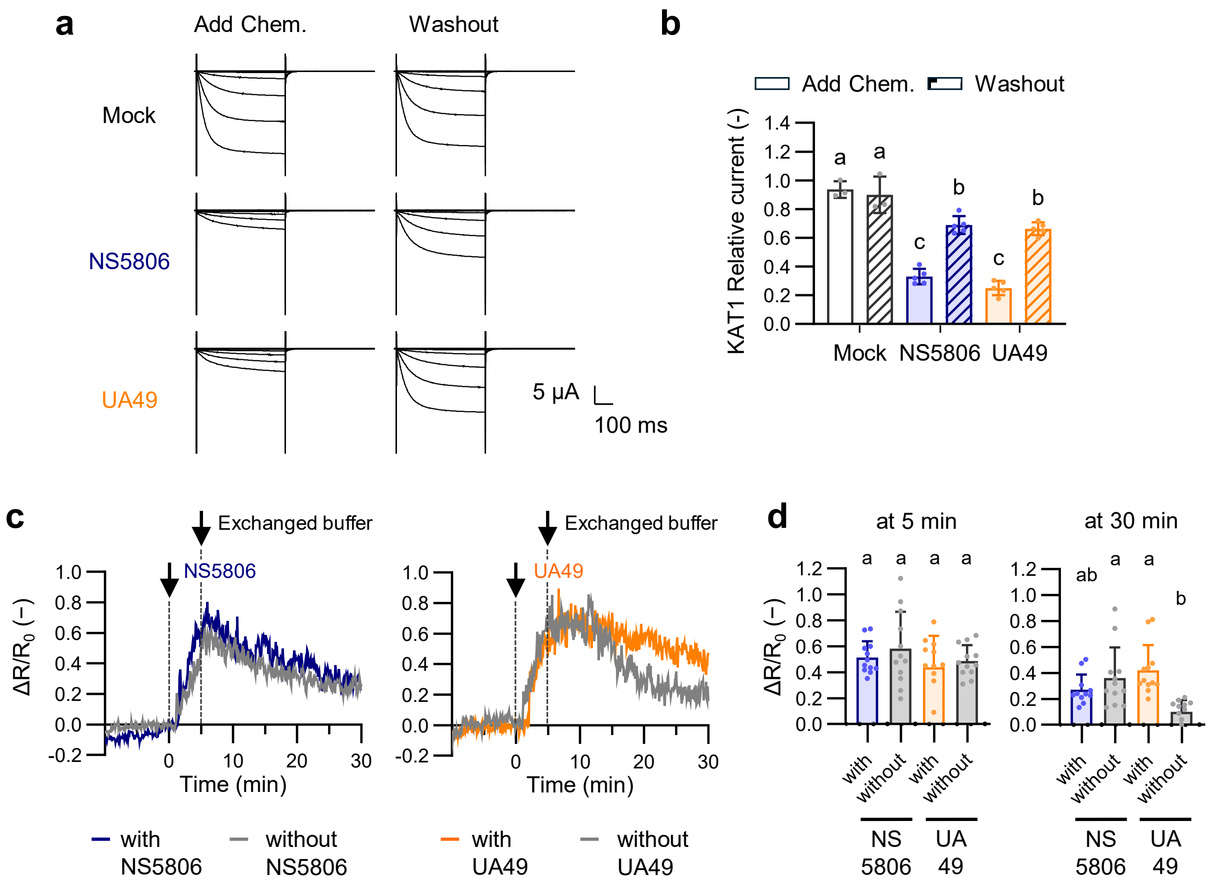

New Data 7: Assessment of the reversibility of NS5806 and UA49 inhibition.

a and **b**, Two-electrode voltage clamp was performed using *Xenopus laevis* oocytes expressing KAT1. Measurements were taken after 1 min of 30 µM NS5806/UA49 treatment and again after a 1-min washout. The representative current traces are shown on **a** and the calculated relative current are shown on **b**. The current value was collected at the end of the pulse at −170 mV (n = 3-5, mean ± SD). **C** and **d**, Calcium imaging on guard cell was performed using the Arabidopsis NES-YC3.6 transgenic line. Guard cells were treated with NS5806/UA49 at 0 min and 75% of the incubation buffer was exchanged with or without 10 µM NS5806/UA49 buffer at 5 min as indicated in the graph. The representative time course data are shown on **c** and the peak cpVenus/CFP ratios as $\Delta R/R_0$ maximum are shown in **d**. Data are plotted as mean ± SD (n = 11-12). Bars marked with different letters are significantly different ($p < 0.05$) by one-way ANOVA with Tukey–Kramer test.

3. Line 123 and fig. 1c: again, compared to KAT1, KAT2 is not inhibited at all by NS5806! -Why?

Response: Thank you for your comment regarding the molecular basis of the inhibitor specificity. This is indeed a central question of our study. Our data show a clear distinction in selectivity: NS5806 is highly specific to KAT1, whereas UA49 inhibits both KAT1 and KAT2 (Fig. 1c). As demonstrated in our chimeric experiments (New Data 5c), replacing the low-homology C-terminal region of KAT1 with that of KAT2 significantly attenuated the inhibitory effect of NS5806. This leads us to hypothesize that the divergent sequences within the C-terminal region—particularly following the CNBHD—act as a critical determinant for the KAT1-specific action of NS5806. However, given that the reciprocal swap (KAT2 with the KAT1 C-terminus) did not confer NS5806 sensitivity, we believe that the binding site or the mechanism of inhibition may involve complex three-dimensional interactions between the C-terminus and other channel domains. We agree that further detailed investigations, such as high-resolution structural analysis (e.g., cryo-EM or X-ray crystallography) or computational docking, will be essential to fully elucidate the precise interaction sites between these compounds and the channel proteins.

4. Apparently, NS5806/UA49 can both promote stomatal closure and inhibit stomatal opening. Is fusicoccin mediated stomatal opening inhibited, too?

Response: To evaluate the physiological potency of NS5806 and UA49 against a strong opening stimulus, we performed stomatal aperture assays using epidermal strips pre-treated with fusicoccin (FC), a well-known activator of the plasma membrane H^+-ATPase (New Data 8). Following a 2-hour pre-treatment with FC, the stomatal closure normally induced by NS5806 was completely abolished. In contrast, UA49 was able to compete with a low concentration of FC (0.1 µM), resulting in significant stomatal closure despite the presence of the opening stimulus.

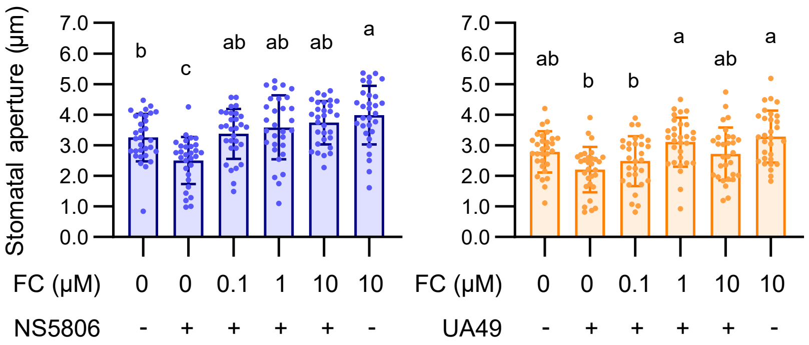

New Data 8: Stomatal responses by NS5806 and UA49 treatments with fusicoccin (FC) under light-to-light condition (test for inducement of stomatal closure).

The stomatal aperture was measured using *A. thaliana* wild type and that of mutants. Epidermal strips were pre-incubated for 2 h under light to induce stomatal opening and followed by a 2 h incubation under light with FC. Subsequently, 10 µM NS5806 or UA49 were added in the sample buffer and incubated 2 h (n = 30-31). Data are shown as mean ± SD. Bars marked with different letters are significantly different ($p < 0.05$) by one-way ANOVA with Tukey–Kramer test.

5. "NS5806/UA49 treatment resulted in a decrease in the cytosolic K^+ concentration in guard cells". Is this decrease sustained or transient, does it compare to kincless!!

Response: To further substantiate the mode of action and the target specificity of our compounds, we monitored the dynamics of cytosolic K^+ concentration ($[K^+]_{cyt}$) using lc-LysM GEPII1.0 plant and performed genetic analyses using the *kincless* mutant. Our time-lapse analysis revealed that the reduction in $[K^+]_{cyt}$ induced by NS5806/UA49 is not transient but persists for at least 50 min (New Data 9a), indicating a sustained inhibitory effect on K^+ homeostasis. Crucially, when we performed stomatal aperture assays using the *kincless* mutant, the stomatal closure typically induced by NS5806/UA49 was completely abolished (New Data 9b). This genetic evidence demonstrates that inward K^+ channels are the indispensable targets for the physiological action of NS5806/UA49. Taken together, these findings strongly support that NS5806/UA49 promote stomatal closure by targeting the K^+ inward channels.

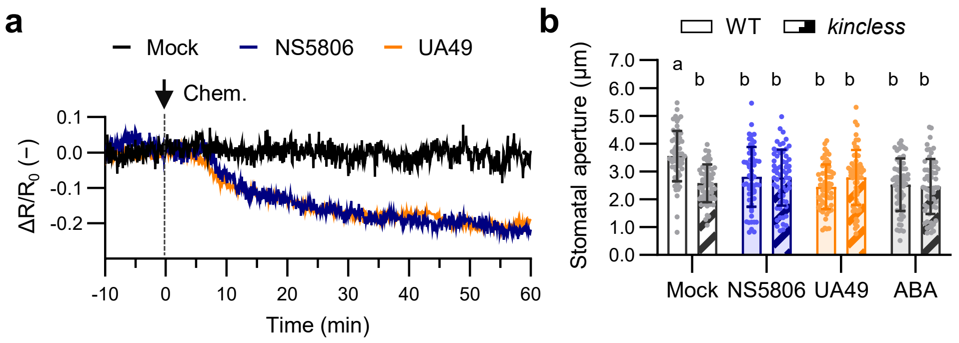

New Data 9: Cytosolic K^+ decrecement and stomatal responses by NS5806 and UA49 treatments under light-to-light condition (test for inducement of stomatal closure).

a, Time course data of representative normalized cpVenus/CFP ratios in guard cells expressing lc-LysM GEPII 1.0 for measuring $[K^+]_{cyt}$ ($n = 24$). The leaf epidermal strips were treated with 0.2% DMSO (Mock), 10 µM NS5806, or UA49 at 0 min. **b**, The stomatal aperture was measured using *A. thaliana* wild type and that of mutants. Epidermal strips were pre-incubated for 2 h under light to induce stomatal opening and followed by a 2 h incubation under light with 0.2% DMSO (Mock), 10 µM NS5806, UA49, or ABA. **b**, *kincless* ($n = 60$). Data are shown as mean ± SD. Bars marked with different letters are significantly different ($p < 0.05$) by one-way ANOVA with Tukey–Kramer test.

6. I am not convinced that the inhibitors act guard cell specific. What about mesophyll or leave/whole plant K^+ content in the long term? Since K^+ homeostasis is crucial for plant development, how do these inhibitors affect plant development, flowering, seed set and productivity?

Response: As shown in Extended Data Fig. 2b and c, these compounds did not affect primary root elongation or seed germination rates, suggesting no broad inhibitory effect on plant development. Furthermore, we conducted Ca^{2+} imaging in different cell types using NES-YC3.6-expressing plants (New Data 10). Unlike the Ca^{2+} increasement observed in guard cells, the application of NS5806 or UA49 did not induce any detectable changes in cytosolic Ca^{2+} levels in pavement cells or root tips. Given that the primary targets, KAT1 and KAT2, are predominantly expressed in guard cells, these results support the conclusion that the physiological effects of NS5806 and UA49 are highly specific to guard cell K^+ channels and their downstream signaling pathways. We added New data 10 as Extended Data Fig. 8 and the text below in the Manuscript Line #206-209.

Added sentence

"The increase in cytosolic Ca^{2+} levels by NS5806/UA49 was not observed in pavement cells or root tips (Extended Data Fig. 8), supporting the conclusion that the action of NS5806/UA49 occurs rather specifically in guard cells."

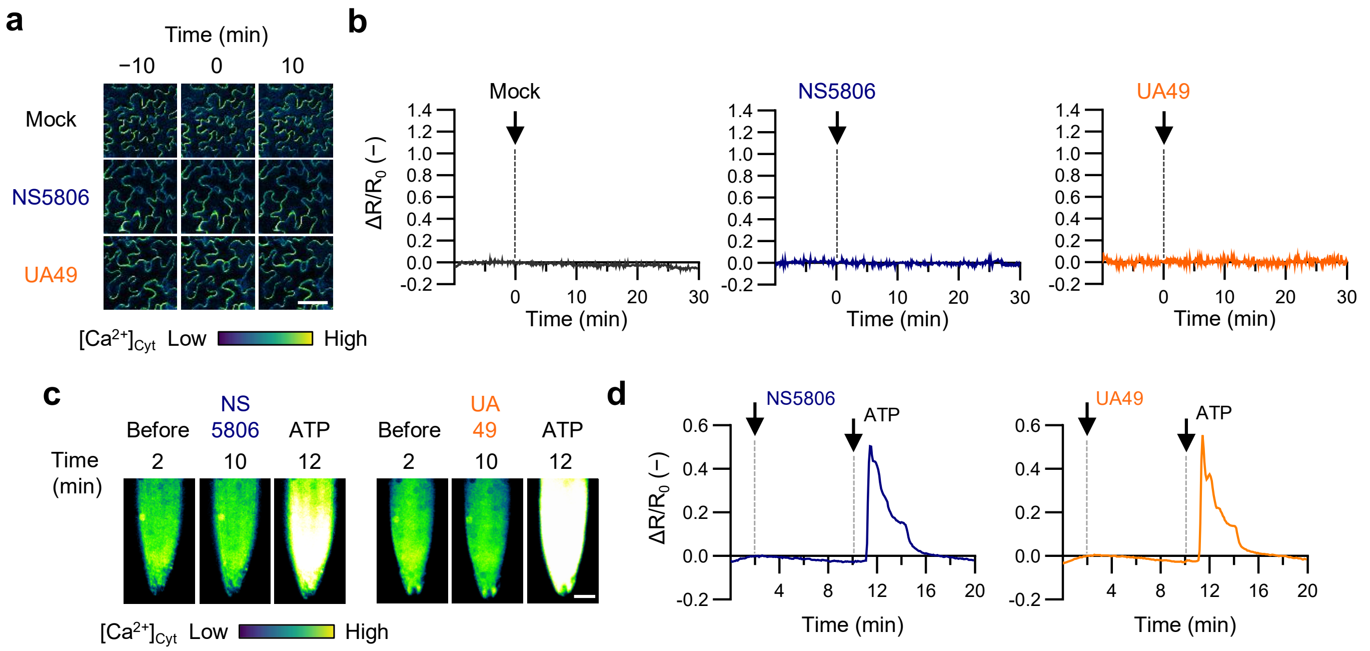

New Data 10 (Extended Data Fig. 8): Tissue specificity of cytosolic Ca^{2+} dynamics triggered by NS5806 and UA49.

a and **b**, The changes of cytosolic Ca^{2+} levels in epidermal pavement cells in response to NS5806/UA49 treatment. **a**, Representative false-color images displaying the cpVenus/CFP emission ratios of NES-YC3.6. Data were collected for 40 min, with 10 µM NS5806/UA49 applied to the imaging solution 10 min after the onset (defined as 0 min on the labels). Scale bar = 50 µm. **b**, Representative time course of cytosolic Ca^{2+} level of epidermal pavement cells. Data were collected from 24 guard cells. **c** and **d**, The changes of cytosolic Ca^{2+} levels in root cells in response to NS5806/UA49 treatment. **c**, Representative false-color images displaying the cpVenus/CFP emission ratios of NES-YC3.6. Data were collected for 20 min, with 10 µM NS5806/UA49 applied to the imaging solution at 2 min and 1 mM ATP applied at 10 min after the onset. **d**, Representative time course of cytosolic Ca^{2+} level of root cells. Data were collected from 3 plants. Scale bar = 50 µm.

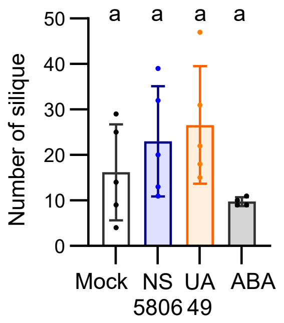

New Data 11: The effect of NS5806 and UA49 on silique formation.

2-3 week olds plant were treated with 500 µL of 0.1% DMSO (Mock), 100 µM NS5806, UA49, or 10µM ABA. Bars marked with different letters are significantly different ($p < 0.05$) by one-way ANOVA with Tukey–Kramer test (n = 4-5).

7. Gene expression analysis is not very informative at this stage. How do guard cell expression profiles compare to those of leaves (mesophyll)? In fig. 3f clustering should be performed to obtain a better picture about differentially expressed transcripts. Gene lists should be provided as suppl. data.

Response: We thank the reviewer for this insightful suggestion. We agree that our gene expression analysis, which was not performed specifically on isolated guard cells, provides limited direct information regarding the molecular mechanisms of NS5806 and UA49 action. Our original intention in including these data was simply to provide a broad comparison to show that the overarching modes of action of NS5806/UA49 differ from those of ABA. Acknowledging that these data are peripheral to the primary mechanistic study of KAT1 inhibition, we have moved the gene expression results to the Extended Data section (Extended Data Fig. 3) as suggested. In addition, a gene list was created in an Excel file. This adjustment allows the manuscript to focus more clearly on the rapid signaling and electrophysiological characterization of the compounds. In addition, a gene list was created in an Excel file.

8. Inhibitor induced stomatal closing is different from ABA. GO analysis shows that differentially addressed genes comprise wounding and JA related pathways. Is inhibitor mediated stomatal closing impaired in JA signaling mutants?

Response: We are sincerely grateful to the reviewer for suggesting the potential involvement of the jasmonic acid (JA) signaling pathway. Following this suggestion, we performed stomatal aperture assays using the JA-insensitive mutant of CORONANTINE INSENSITIVE1 (COI1), *coi1-1* (New Data 12). Interestingly, we found that the NS5806-induced stomatal closure was significantly impaired in *coi1-1*, indicating that NS5806 requires a functional JA signaling pathway to exert its full effect. In contrast, UA49-induced stomatal closure remained unaffected in the *coi1-1* mutant, suggesting that UA49 operates through a mechanism independent of both ABA and JA signaling pathways. This discovery provides a novel layer of understanding regarding the distinct modes of action for these two compounds. While both compounds inhibit KAT1, NS5806's physiological effect is integrated with JA-mediated responses, whereas UA49 acts as a more direct or broadly independent modulator of stomatal movement.

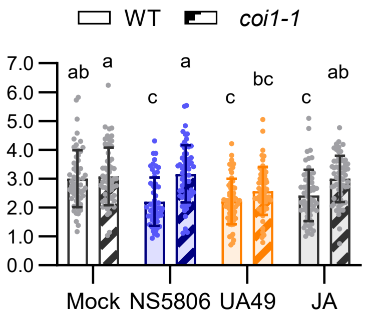

New Data 12: Stomatal responses by NS5806 and UA49 treatments under light-to-light condition (test for inducement of stomatal closure).

The stomatal aperture was measured using *A. thaliana* wild type and *coi1-1*. Epidermal strips were pre-incubated for 2 h under light to induce stomatal opening and followed by a 2 h incubation under light with 0.2% DMSO (Mock), 10 µM NS5806, UA49, or 20 µM jasmonic acid (JA). Data are shown as mean ± SD. Bars marked with different letters are significantly different ($p < 0.05$) by one-way ANOVA with Tukey–Kramer test (n = 56-60).

9. Inhibitor induced stomatal closure is impaired in the slac1 mutant, a convergence point for many pathways addressing stomatal movements. Stomatal closure requires the concerted release of anions and potassium. The latter is mediated by K-efflux channels like GORK, which is also addressed by the inhibitors (see fig 1). What is the role of K-efflux channels in this process?

Response: This is a crucial yet challenging question regarding the hierarchy of ion channel regulation during compound-induced stomatal closure. Our genetic analysis showed that the stomatal closure induced by NS5806/UA49 was impaired in both the *kincless* (New Data 9b) and the *gork* (New Data 13), which suggests that both K^+ inward channels KAT1/KAT2 and the outward K^+ channel GORK are indispensable for the full physiological response for NS5806/UA49. However, it is still unclear which is controlled first by the compound. Multiple lines of evidence suggest that KAT1/KAT2 are the primary targets of these compounds rather than GORK. In *Xenopus* oocyte assays, both compounds showed a stronger inhibitory effect on KAT1 compared to GORK (Fig. 1c). Also, direct patch-clamp recordings from guard cells confirmed that K_{in} currents were markedly suppressed upon compound application (Fig. 1d). Based on these results, we hypothesize that NS5806 and UA49 primarily target KAT1/KAT2 to trigger the closure process (likely involving the observed Ca^{2+} increase), while GORK functions as the essential downstream effector for K^+ efflux. Without functional GORK, the cell cannot effectively reduce its turgor pressure, which explains the lack of response in the *gork* even if the primary target is inhibited.

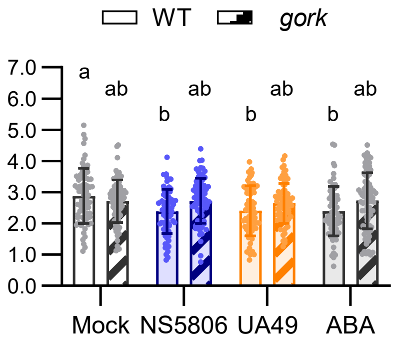

New Data 13: Stomatal responses by NS5806 and UA49 treatments under light-to-light condition (test for inducement of stomatal closure).

The stomatal aperture was measured using *A. thaliana* wild type and *gork*. Epidermal strips were pre-incubated for 2 h under light to induce stomatal opening and followed by a 2 h incubation under light with 0.2% DMSO (Mock), 10 µM NS5806, UA49, or ABA. Data are shown as mean ± SD. Bars marked with different letters are significantly different ($p < 0.05$) by one-way ANOVA with Tukey–Kramer test ($n = 80$).

10. Related to the question above. Is it possible that these inhibitor act as activators of anion channels?

Response: We further examined the selectivity of our compounds by testing their effects on the anion channels SLOW ANION CHANNEL-ASSOCIATED 1 (SLAC1) and SLAC1 HOMOLOGUE 3 (SLAH3) (New Data 14). We found a clear difference in their selectivity profiles: NS5806 showed no inhibitory effect on either SLAC1 or SLAH3. In contrast, UA49 exhibited a significant inhibitory effect on SLAC1, although it did not affect SLAH3. These results indicate that while both compounds effectively inhibit KAT1, NS5806 possesses a higher degree of specificity toward K^+ channels compared to UA49, which shows some cross-reactivity with the anion channel SLAC1.

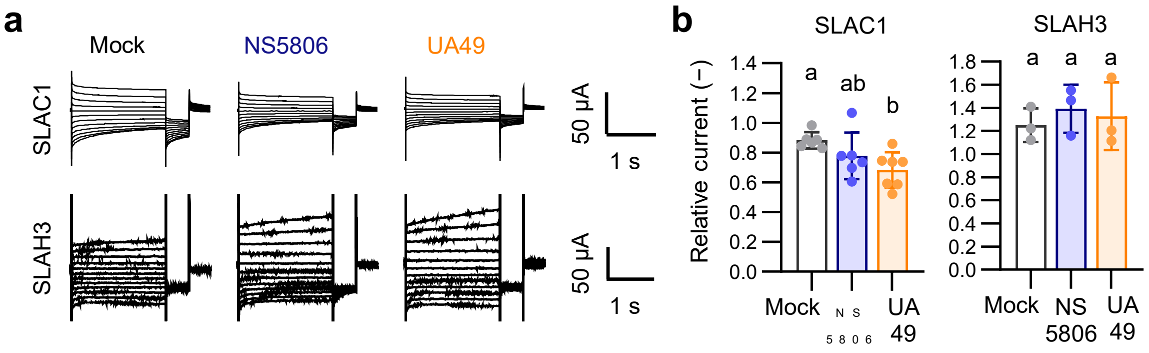

New Data 14: Selectivity of NS5806 and UA49 toward anion channels and orthologous K^+ channels.

Two-electrode voltage clamp was performed using *Xenopus laevis* oocytes expressing Arabidopsis anion channels SLAC1 or SLAH3. SLAC1 was co-expressed with OST1 fused to 10-amino-acid fragment derived from CBL1. Whole-cell currents were recorded during voltage pulses lasting 2 s and ranging from +40 to −180 mV in 20 mV decrements, followed by a 500 ms voltage pulse at −120 mV. The recording buffer was composed of 75 mM NaCl, 20 mM $CaCl_2$, 1 mM $MgCl_2$, 10 mM HEPES, with the osmolarity adjusted to 220-260 mM mOsm using D-mannitol. The current value was collected at 50 ms of the pulse at −180 mV. SLAH3 was co-expressed with CBL1 and CIPK23. Whole-cell currents were recorded by the same voltage pulse protocol as SLAC1. The recording buffer was composed of 100 mM $NaNO_3$, 1 mM $Ca(gluconate)_2$, 1 mM Mg $(gluconate)_2$, 1 mM $LaCl_3$, 10 mM Tris, with the osmolarity adjusted to 220-260 mM mOsm using D-sorbitol. The current value was collected at 5 ms of the pulse at −180 mV. **a**, Representative current traces. **b**, Calculated relative currents (n =3-7). The current value was collected at 50 ms of the pulse at −180 mV for SLAC1, or at 5 ms of the pulse at −180 mV for SLAH3. Bars marked with different letters are significantly different ($p < 0.05$) by one-way ANOVA with Tukey–Kramer test.

11. It appears puzzling that inhibitors do not affect guard cell Ca^{2+} channels but induce a sustained Ca^{2+} signal in guard cells. The causal relationship between Ca^{2+} signaling and inhibitor induced stomatal closure has not been provided, but needs…

Response: We agree with the reviewer that a direct causal link such as demonstrating the rescue of the closure phenotype by Ca^{2+} signaling, has not been explicitly shown in this study. However, our hypothesis, which positions Ca^{2+} signaling downstream of KAT1/KAT2 inhibition, is supported by several independent lines of evidence and established literature. It has been documented that the regulation of intracellular K^{+} levels can trigger Ca^{2+} influx (Allen *et al.*, *Nature* (2001)). Furthermore, previous studies have successfully induced stomatal closure by artificially generating intracellular Ca^{2+} signals (Huang *et al.*, *Sci. Adv.* (2021), Huang *et al.*, *Curr. Biol.* (2024)). These findings align with our hypothesis that KAT1/KAT2 inhibition disrupts the ionic/electrical balance, thereby recruiting Ca^{2+} as a secondary messenger. The fact that NS5806/UA49 failed to elicit Ca^{2+} increase in the *kincless* mutant (Fig 5h, i) suggests that the presence of functional inward K^{+} channels is a prerequisite for these Ca^{2+} signals. This suggests that KAT1/KAT2 serve as the initial "sensors" or "voltage regulators" required to drive the subsequent Ca^{2+} influx. Following the reviewer's insightful suggestion, we discovered that NS5806/UA49-induced closure is dependent on the JA signaling pathway (New Data 12, *coi1-1*). Since JA signaling is known to induce stomatal closure via Ca^{2+} transients during stress responses (Forster *et al.*, *Dev. Cell.* (2018)), our observation of inhibitor-induced Ca^{2+} increase is physiologically consistent with known plant defense mechanisms.

12. Do other K^+ channels inhibitors like TEA or Cs act in a comparable manner in mediating a Ca^{2+} response and induction of stomatal closure? If not, what distinguishes the new inh

Response: TEA and CsCl inhibited KAT1/KAT2 activity (New Data 15a, b) and induced stomatal closure (New Data 15c, e). However, unlike NS5806/UA49, these compounds did not cause the intracellular Ca^{2+} increase (New Data 15d, f). We attribute this striking difference to the degree of target specificity. TEA and CsCl are broad-spectrum, "non-specific" K^+ channel blockers, whereas NS5806 and UA49 appear to be highly specific to KAT1/KAT2 (Fig. 1c).

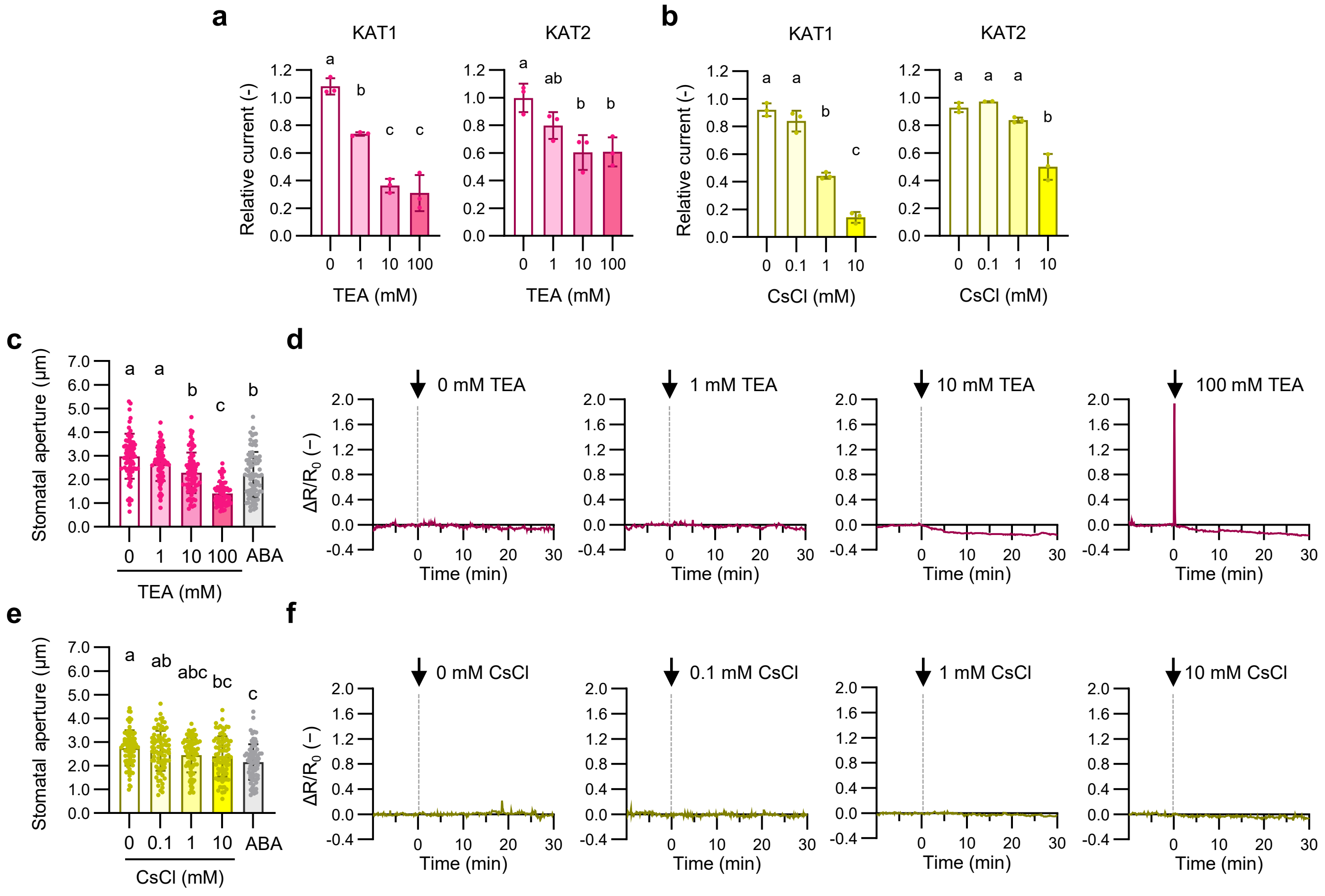

New Data 15: The effect of tetraethylammonium (TEA) and CsCl on KAT1/KAT2 and guard cells.

a and **b**, Two-electrode voltage clamp were performed for KAT1 and KAT2. Dose dependency was acquired for TEA (**a**) and CsCl (**b**) (n = 2-3). **c**, Stomatal aperture assay of wild-type *Arabidopsis thaliana* in response to TEA (n = 77-80). **d**, Representative time course of cytosolic Ca^{2+} imaging in response to TEA treatment using a transgenic Arabidopsis line expressing NES-YC3.6. Data were collected from 24 guard cells. **e**, Stomatal aperture assay of wild-type *Arabidopsis thaliana* in response to CsCl (n = 80-81). **f**, Representative time course of cytosolic Ca^{2+} imaging in response to CsCl treatment using a transgenic Arabidopsis line expressing NES-YC3.6. Data were collected from 24 guard cells. Bars marked with different letters are significantly different ($p < 0.05$) by one-way ANOVA with Tukey–Kramer test.

13. In addition, inhibitor induced Ca^{2+} signaling is impaired in the kincless mutant lacking functional K^+ channels due to the heteromerization potential of the underlying KAT1 mutant. Kout channels however do not assemble into heteromers with Kin channels, pointing to a possible important role of Kout channels. This issue has not been clarified (see above).

Response: Our stomatal aperture test using *kincless* and *gork* mutants indicates that both inward and outward K^+ channels are indispensable for the stomatal closure induced by NS5806 and UA49 (New Data 9b, 13). However, based on the potent inhibition of KAT1 observed in *Xenopus* oocytes (Fig. 1c) and the marked suppression of K_{in} currents in guard cell patch-clamp assays (Fig. 1d), KAT1/KAT2 would be the primary molecular targets. We hypothesize that NS5806 and UA49 primarily target KAT1/KAT2 to initiate the closure process—likely involving the observed Ca^{2+} transients—while GORK functions as the essential downstream executor for K^+ efflux and subsequent cell shrinkage.

14. What is the effect of the inhibitors on guard cell/mesophyll cell membrane potential? Do they really result in hyperpolarization as suggested? Please test.

Response: To investigate the impact of NS5806 and UA49 on the plasma membrane potential of guard cells, we utilized the voltage-sensitive dye $DiBAC_4(3)$. While ABA application induced an increase in fluorescence, indicating the typical depolarization, UA49 treatment led to a significant quenching of the fluorescence (New Data 16). This observation indicates that UA49 induces membrane hyperpolarization, which is consistent with our hypothesis that the compound prevents K^+ influx by inhibiting KAT1 and KAT2. Interestingly, NS5806 did not trigger a detectable change in this assay system, suggesting that UA49 exerts a more potent influence on the overall membrane potential. These findings differentiate the pharmacological action of our compounds from the ABA-mediated signaling pathway and underscore their specificity toward inward K^+ channels.

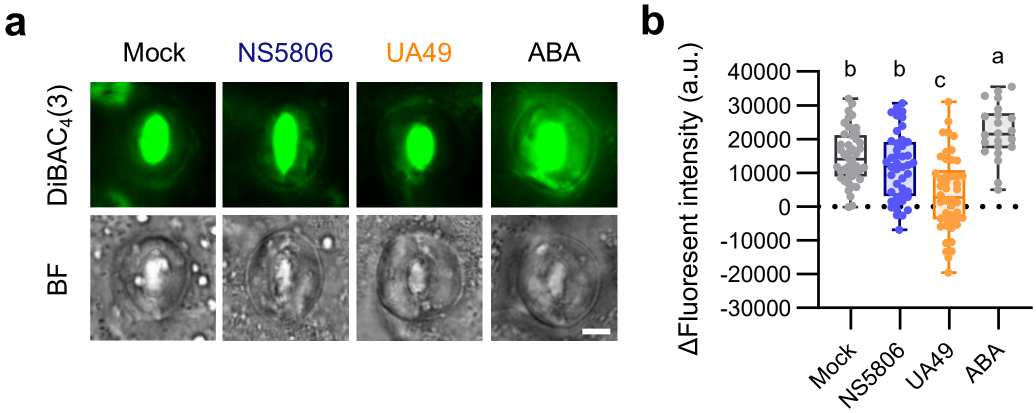

New Data 16: UA49 induces plasma membrane depolarization in guard cells.

Epidermal strips from wild-type *Arabidopsis thaliana* were pre-treated with 2 μM $DiBAC_4(3)$ for 20 min. The samples were excited at 488 nm, and fluorescence images were acquired using 525/50 nm band-pass filter. Images were captured prior to and 10 min after the application of the compounds to quantify the change in fluorescence intensity. **a**, Representative image of guard cells after the NS5806/UA49 treatment. Scale bar = 10 μm. **b**, Relative changes in fluorescence intensity (n = 22-46). Bars marked with different letters are significantly different ($p < 0.05$) by one-way ANOVA with Tukey–Kramer test.

15. Do these inhibitors really act in a guard cell specific manner and locally or do they act also in a systemic manner? Would the inhibitors affect K+ channels from other plant species or would even act cross species wide (e.g. insect K^+ channels)

Response: We thank the reviewer for pointing out the importance of tissue and target specificity. Our new data further strengthen the conclusion that NS5806 and UA49 are highly specific to guard cells within the plant body. As shown in New Data 10, these compounds did not elicit any detectable Ca^{2+} increase in pavement cells or root tips, confirming that their physiological action is restricted to guard cells expressing the target K^+ channels. Furthermore, to explore the broader applicability of these inhibitors, we tested their effects on the *Drosophila* Shaker B channel expressed in *Xenopus* oocytes. Remarkably, both NS5806 and UA49 significantly inhibited the Shaker B current (New Data 17). This cross-kingdom inhibition suggests that the compounds target a conserved structural feature within the Shaker-type K^+ channel superfamily, rather than a plant-specific regulatory site. These findings not only validate our model in Arabidopsis but also suggest that NS5806 and UA49 may serve as versatile tools for modulating Shaker-type channels across a wide range of species.

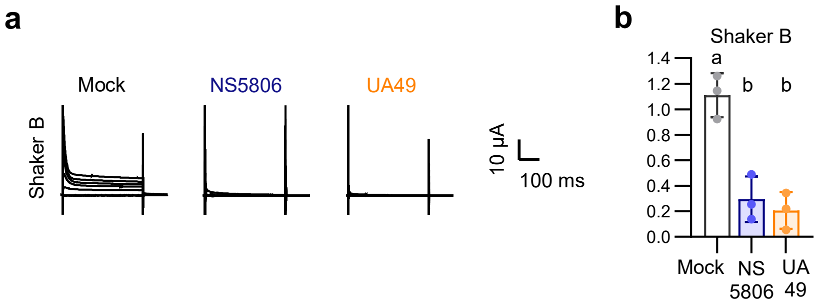

New Data 17: Selectivity of NS5806 and UA49 toward anion channels and orthologous K^+ channels.

Two-electrode voltage clamp was performed using *Xenopus laevis* oocytes expressing K^+ channel Shaker B from *Drosophila melanogaster*. Whole-cell currents were recorded from a holding potential of −80 mV. Test pulses (300 ms) were applied from −80 mV to +60 mV in 20 mV steps. The recording buffer was composed of 140 mM NaCl, 2 mM KCl, 2 mM MgCl2, and 10 mM HEPES-NaOH (pH 7.2). Oocytes were treated with 30 µM NS5806/UA49 for 1 min. **a**, Representative current traces. **b**, Calculated relative currents (n =3-7). The current value was collected as an instantaneous current value (5 ms after the initiation of voltage pulse) at −170 mV. Bars marked with different letters are significantly different ($p < 0.05$) by one-way ANOVA with Tukey–Kramer test.

16. In the Ca^{2+} imaging movies guard cells appear open and do not close, please comment.

Response: Thank you for pointing this out. We agree that the morphological change in stomatal aperture may not be readily apparent in the provided Ca^{2+} imaging movies. In this study, time-lapse imaging was performed for 30 minutes to capture the rapid dynamics of the Ca^{2+} signaling response for compounds. However, the NS5806-induced stomatal closure is a gradual process; at the 30 min mark, the reduction in stomatal aperture is only 17.8% (New Data 18). Because the primary purpose of these movies is to demonstrate the immediate Ca^{2+} transients following compound application, the 30 min window focuses on the signaling phase rather than the complete morphological closure, which typically requires a longer duration to be visually prominent in a time-lapse format.

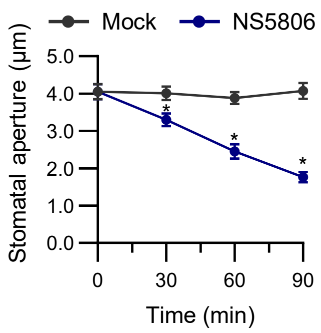

New Data 18: The time course data of stomatal responses by NS5806 treatments under light-to-light condition (test for inducement of stomatal closure).

The stomatal aperture was measured using *A. thaliana* wild type and that of mutants. Epidermal strips were pre-incubated for 2 h under light to induce stomatal opening and followed by a 2 h incubation under light with 0.2% DMSO (Mock) or 10 μM NS5806. Data are shown as mean ± SEM (n = 60). Statistical significance was determined using a two-tailed unpaired Student's *t*-test (*$p < 0.05$).

17. Fig. 4g, h is figure 5

Response: We apologize for the typographical error in the figure citation at line# 224. The reference to "(Fig. 4g, h)" has been corrected to "(Fig. 5h, i)" in the revised manuscript.

18. …effect on stomatal closure implies previously unrevealed cross-talk between K^+ channels, anion channels, and Ca^{2+} channels. Actually, this find is not new. The potassium/calcium interrelationship is well known and has been used by the authors to impose potassium dependent calcium changes to plant cells.

Response: Thank you for your comment. We agree with what the reviewer said, so we deleted the relevant sentence in the discussion section.

Deleted original sentence in Manuscript Line #247

"The effect of NS5806/UA49 on stomatal closure implies previously unrevealed cross-talk between K^+ channels, anion channels, and Ca^{2+} channels."

Other editions:

-Updated the affiliation for Takumi Higaki. Due to a recent organizational restructuring, his previous two affiliations have been consolidated into a single institution.

-Updated the affiliation for Yasuhiro Ishimaru. Since his current affiliation differs from the one at the time of the initial submission, we have added his current institution as a 'Present address' in the revised manuscript.

-Figure legends for Fig. 1. (Manuscript Line #605 and 608): We apologize for our spelling mistake. We corrected "*leaves*" to "*laevis*".

-Fig. 1e: We apologize for our mislabeling. We have corrected a statistical error in the central panel. In the previous version, an asterisk (*) was included to indicate a statistically significant difference between the Mock and UA49 treatments at −150 mV. The revision does not affect the conclusions of our study.

-We have added a description of the H_2O_2 data in the revised manuscript (Line #132). Although H_2O_2 was included in the original Fig. 1e as a positive control, it was not mentioned in the text.

-Fig. 5h: We apologize for our mislabeling. The term "Mock" has been replaced with "WT".

-To clarify the indicator used in Ca^{2+} imaging, we have added labels for the Ca^{2+} sensors "NES-YC3.6" and "ER-GCaMP6-210" directly above the relevant panels in Fig. 5b, f, and h.

-We have updated the Acknowledgments section to include "a grant from the Deutsche Forschungsgemeinschaft (468861065 to J.K.)" (Line#509-510).

-We have included a list of all Arabidopsis lines used in this study as a supplemental table within the Supplementary Information.

Response to Reviewers:

Thank you very much for assessing our manuscript entitled "Synthetic ion channel inhibitors enhance plant drought tolerance" (NCOMMS-25-17471A). Based on the helpful editor's and reviewers' comments, we are now submitting the revised version. The specific changes made are detailed in our enclosed "Point-by-point response to the reviewers' comments" where we have reproduced their feedback verbatim alongside our responses.

REVIEWER COMMENTS

Reviewer #3 (Remarks to the Author):

Review on the revised manuscript by Sato et al. entitled 'Synthetic ion channel inhibitors enhance plant drought tolerance'

First, I would like to express my satisfaction with the manner and extent to which the authors have answered the open questions. The revised manuscript in its present form has improved significantly.

Response: We sincerely appreciate the constructive feedback provided during the review process, which has greatly helped us improve the quality and clarity of our work.

The following issues have been addressed and were answered extensively based on new experimental data:

1. Inhibitor site of action

Assuming the inhibitors could act on the cytosolic site of Kin channels, domain swapping approaches between KAT1/KAT2 identified the C-terminal region of KAT1 to be involved in the inhibitory mechanism of NS5806. Unfortunately, the reciprocal chimera did not show increased sensitivity to NS5806. Likewise, UA49 exhibited consistent inhibition across all chimeric variants.

On the other hand, the authors could convincingly demonstrate the reversibility of inhibitor action in wash-out experiments, which would suggest that these compounds primarily influence channel activity from the extracellular side of the plasma membrane. These data fit well the observed inhibition of animal Shaker channels that exhibit structurally different C-termini compared to plant Kin channels.

This important finding should be included in the manuscript (discussion)

Response: Thank you very much for your valuable suggestions. As you noted, our additional data in the "Point-by-point response to the reviewers' comments" suggest that NS5806 and UA49 primarily influence channel activity from the extracellular side of the plasma membrane. However, the KAT1–KAT2 domain-swapping experiments focusing on the C-terminal regions did not yield fully consistent results. In addition, the membrane permeability of these inhibitors remains unclear, and the current data do not allow us to draw firm conclusions at this stage. Given these limitations, we are concerned that including these preliminary findings might introduce some confusion rather than provide clear insight for readers. We feel that a more definitive interpretation will likely require future structural studies. Therefore, we would like to opt to exclude these data from the manuscript.

2. Inhibitor mode of action

Re-evaluation of the electrophysiological data strongly suggest that the inhibitors act as gating modifiers. This is an important observation adding to the understanding of stomatal physiological responses is not found in the revision. Since the authors have also addressed the dose dependency it would be helpful getting the IC50 values.

Response: We appreciate your suggestions and have revised the manuscript accordingly. Given the complexity of the inhibitory mechanisms, a detailed explanation in the main text might disrupt the narrative flow. Therefore, we have included relatively brief descriptions in the results section (Line #126-128) and moved the New Data 3 and New Data 6 in the "Point-by-point response to the reviewers' comments" to Extended Data Fig. 2 and Extended Data Fig. 3. The poor solubility of these compounds prevented the precise calculation of IC50 values.

Added sentence

"NS5806 and UA49 inhibited KAT1 in a dose-dependent manner (Extended Data Fig. 2), and the shifts in the tail current I-V relationships suggest a potential effect on channel gating (Extended Data Fig. 3). "

3. Specificity

In new experiments the authors now show that the inhibitors inhibit guard cells specifically and this information is now found in the manuscript.

Response: We are grateful for the reviewer's insightful comments, which helped us enhance the quality of our work.

4. Physiological context

a) In new experiments the authors observed that following a 2-hour pre-treatment with FC, the stomatal closure normally induced by NS5806 was completely abolished. Thus, fusicoccin could overcome inhibitor mediated inhibition of blue light dependent H+-ATPase phosphorylation (Fig. 3). On the other hand, and based on DiBAC data, UA49 seems to induce membrane hyperpolarization, which would require pronounced H+ ATPase activity. Still, this finding adds to the understanding of mode of inhibitor action and would fit to the observed shift in voltage dependent gating triggered by the inhibitors – an information not found in the revised version.

Response: Thank you very much for your comments. We think that the current data on FC-induced stomatal opening and membrane potential are insufficient to draw a definitive conclusion regarding the extent and timing of how NS5806 or UA49 affects FC signaling. To avoid overly speculative discussion, we would like to refrain from including these points in the revised manuscript.

b) In another set of new experiments, the authors show that when using kincless and gork mutants, stomatal closure typically induced by NS5806/UA49 was completely abolished. This strongly supports the idea that suggests that both K+ inward channels KAT1/KAT2 and the outward K+ channel GORK are indispensable for the full physiological response – an information that is not found in the revised version.

Response: Thank you very much for your kind suggestions. We have added the New Data 9b and New Data 13 in the "Point-by-point response to the reviewers' comments" as Extended Data Fig. 12 and the explanation in the Manuscript (Line #226-229).

Added sentence

"Accordingly, stomatal closure induced by NS5806/UA49 was impaired in the *kincless* mutant (Extended Data Fig. 12a). Interestingly, NS5806/UA49-induced stomatal closure did not occur in the *gork* mutant, suggesting that GORK is also involved in the stomatal closure mediated by these compounds (Extended Data Fig. 12b)."

c) Finally, new experiments demonstrate that NS5806-induced stomatal closure was significantly impaired in coi1-1, indicating that this inhibitor requires a functional JA signaling pathway to exert its full effect. However, UA49-induced stomatal closure seems JA independent and remained unaffected in the coi1-1 mutant. This is an important physiological context and thus the inhibitors may help to dissect the hormone signaling pathway in guard cells. Please discuss.

Response: Thank you very much for your comments. Based on our RNA-seq data suggesting the involvement of the JA pathway in NS5806/UA49-induced stomatal closure, we performed additional experiments using the *coi1-1* mutant. The results indicated that NS5806 is involved in the JA pathway, while UA49 appears to be only partially involved. These findings suggest that NS5806 and UA49 are involved in phytohormone signaling pathways, and the potential impact on other hormones beyond JA and ABA warrants further investigation. However, we feel that the current data are not yet sufficient to provide a comprehensive discussion on the relationship between JA (*coi1-1*) and NS5806/UA49. To maintain the focus and scientific rigor of the paper, we would like to refrain from including these specific results and detailed comments in the current manuscript.

In summary, the authors have addressed the open questions and points of criticism with great care, considerable effort and a significant number of new experiments. Like always, several questions remain. For example it is not sufficient to block Kin channels (see Cs+ and TEA data) to induce Ca+ signals. The pharmacological data employing animal Shaker K channels also may point towards a neurotoxic function of these inhibitors, requiring caution in the context of using the inhibitors in their current states for agricultural purposes.

Response: We appreciate your constructive feedback and will use your insights to guide our future experiments.

Other editions:

-We added the information of *kincless* mutant and *gork* mutant in “Supplementally Information”.

We have refined the wording throughout the manuscript as follows. These changes do not affect the original meaning or the scientific conclusions.

-Line #176: addition “whose expression was”

-Line #202: from “cytoplasmic” to “cytosolic”

-Line #257: addition of “cytosolic”

-Line #258: from “induced” to “induces”

-Line #258: from “a transient increase” to “transient increases”

-Extended Data Fig. 4. Line #734-735: addition “of stomata guard cells”

-Supplemental Table: from “*Arabidopsis* strains” to “*Arabidopsis thaliana* backgrounds”
